# Detection of post-vaccination enhanced dengue virus infection in macaques: An improved model for early assessment of dengue vaccines

**DOI:** 10.1371/journal.ppat.1007721

**Published:** 2019-04-22

**Authors:** Maria Beatriz Borges, Renato Sergio Marchevsky, Renata Carvalho Pereira, Ygara da Silva Mendes, Luiz Gustavo Almeida Mendes, Leonardo Diniz-Mendes, Michael A. Cruz, Ouafaâ Tahmaoui, Sébastien Baudart, Marcos Freire, Akira Homma, Kirsten Schneider-Ohrum, David W. Vaughn, Yannick Vanloubbeeck, Clarisse Lorin, Marie-Pierre Malice, Elena Caride, Lucile Warter

**Affiliations:** 1 Bio-Manguinhos, Fiocruz, Rio de Janeiro, Brazil; 2 Research & Development, GSK Vaccines, Rockville, Maryland, United States of America; 3 Research & Development, GSK Vaccines, Rixensart, Belgium; National Institute of Allergy and Infectious Diseases, UNITED STATES

## Abstract

The need for improved dengue vaccines remains since the only licensed vaccine, Dengvaxia, shows variable efficacy depending on the infecting dengue virus (DENV) type, and increases the risk of hospitalization for severe dengue in children not exposed to DENV before vaccination. Here, we developed a tetravalent dengue purified and inactivated vaccine (DPIV) candidate and characterized, in rhesus macaques, its immunogenicity and efficacy to control DENV infection by analyzing, after challenge, both viral replication and changes in biological markers associated with dengue in humans. Although DPIV elicited cross-type and long-lasting DENV-neutralizing antibody responses, it failed to control DENV infection. Increased levels of viremia/RNAemia (correlating with serum capacity at enhancing DENV infection *in vitro*), AST, IL-10, IL-18 and IFN-γ, and decreased levels of IL-12 were detected in some vaccinated compared to non-vaccinated monkeys, indicating the vaccination may have triggered antibody-dependent enhancement of DENV infection. The dengue macaque model has been considered imperfect due to the lack of DENV-associated clinical signs. However, here we show that post-vaccination enhanced DENV infection can be detected in this model when integrating several parameters, including characterization of DENV-enhancing antibodies, viremia/RNAemia, and biomarkers relevant to dengue in humans. This improved dengue macaque model may be crucial for early assessment of efficacy and safety of future dengue vaccines.

## Author summary

Dengue virus (DENV) is responsible for the most widespread arboviral disease affecting humans. A pre-existing suboptimal immunity to DENV is accepted as being the major risk factor for severe dengue. Thus, if vaccination does not elicit optimal DENV-specific immunity, a vaccine might, instead, increase the risk of severe dengue in vaccinated individuals, as seen with the only licensed vaccine (Dengvaxia) in children naïve to DENV at vaccination. It is thus crucial to assess dengue vaccine safety at the earliest development stages, ideally in the preclinical stage. The dengue macaque model has been used to assess preclinical efficacy of dengue vaccines, with post-challenge DENV replication as the sole efficacy endpoint. However, this model had not predicted the Dengvaxia-associated safety signals. Here we characterized, in macaques, a dengue purified and inactivated vaccine (DPIV) candidate for its immunogenicity and efficacy/safety. Using a multiparameter approach, including characterization of viral replication and biomarkers relevant to dengue/severe dengue in humans, we were able to detect vaccine-associated safety signals in this model. While these results enabled us to discontinue at an early stage the DPIV development, this improved dengue macaque model may also be instrumental for early assessment of efficacy/safety of future dengue vaccines.

## Introduction

Dengue viruses 1–4 (DENV-1-4) are mosquito-borne flaviviruses annually responsible for 50–100 million dengue cases in humans, that have been classified as dengue fever (DF), dengue hemorrhagic fever (DHF) and dengue shock syndrome (DSS), and further re-classified as dengue (with or without warning signs) and severe dengue [1,2]. Pre-existing sub-optimal immunity to DENV is thought to be the strongest risk factor for DHF/DSS, for which antibody-dependent enhancement (ADE) of DENV infection is proposed to be the early underlying mechanism. ADE of DENV infection is posited to occur when pre-existing antibodies bind but do not efficiently neutralize DENV virions, resulting in DENV immune complexes that interact with FcγRs, thus facilitating not only entry but also viral replication (due to FcγR-associated suppression of intra-cellular innate immunity) in FcγR-bearing cells [3,4]. ADE of DENV infection has been observed in infants born to DENV-immune mothers, with a peak of incidence of DHF/DSS correlating with the waning of maternally-acquired antibodies [5,6]. The reported association between specific pre-existing DENV-antibody titers and the risk of DHF/DSS further confirmed the role of ADE in severe dengue [7].

The pre-existing immunity risk factor for DHF/DSS has complicated dengue vaccine development. It is believed that a dengue vaccine needs to elicit protective immune responses against all 4 DENV types while not inducing DENV-enhancing antibodies that could increase the risk of severe disease in vaccinated individuals [8,9]. However, the only licensed dengue vaccine, Dengvaxia, was shown to increase the risk of hospitalization for severe dengue in children naïve to DENV before vaccination, thus further emphasizing the need to assess dengue vaccine safety at the earliest development stages prior to human vaccination [10].

DENV vaccine development has also been impaired by the lack of an optimal animal model reproducing human dengue disease. Although several non-human primate species sustain DENV replication after experimental infection, they rarely develop clinical signs [11–13]. Despite this, the macaque is widely accepted as the most suitable model for preclinical characterization of dengue vaccines. Prior to clinical development, all vaccine candidates to date were tested for efficacy in this model using post-challenge viremia as the sole surrogate for disease [14–19]. Nevertheless, the only dengue vaccine for which both preclinical and clinical efficacy results were reported (Dengvaxia) showed almost 100% efficacy at preventing post-challenge viremia in macaques whereas its efficacy in humans was substantially lower. Although the vaccine was further reported not to prevent post-challenge DENV-2 RNAemia in macaques, no signs of enhanced DENV infection were detected, thus not reflecting the vaccine-associated increased risk for severe dengue reported in children naïve to DENV at vaccination [10,17,20–22]. This suggests that, when determined using the sole post-challenge viral replication, dengue vaccine efficacy in macaques may not predict efficacy and safety in humans.

We hypothesized that the limited predictability of the dengue macaque model might be related to the use of DENV challenge strains isolated long ago and subjected to serial cell passages (thus likely to differ from circulating strains) and/or to the viremia levels that are substantially lower in macaques compared to humans (which could result in an over-estimation of vaccine efficacy in macaques). Another limitation may be the fact that the biomarkers modified in dengue patients are not evaluated, as surrogates of dengue clinical signs, when assessing vaccine efficacy in macaques. Therefore, we previously selected recent and minimally passaged Brazilian DENV clinical isolates (including DENV-1 0111/2011 and DENV-2 0126/2010) which induce robust viremia in macaques, and are associated with changes in cytokine/chemokine profiles sharing some similarities with those reported in DF patients [23]. Finally, the limited predictability of the dengue macaque model may also be explained by the short intervals usually allowed between vaccination and DENV challenge, ranging from 1 (in most studies) to 5–6 months post-vaccination [14,15,17–19]. Indeed, to more accurately predict long-term vaccine efficacy, the challenge should be performed at a sufficiently late time-point post-vaccination, when the dengue-associated short-lasting heterotypic immunity has waned to low/undetectable level, and the vaccine-elicited immunity has reached its low plateau level.

Here we evaluated, in rhesus macaques, a tetravalent dengue purified and inactivated vaccine (DPIV) candidate for its immunogenicity and efficacy to control infection following DENV challenge. We compared between vaccinated and non-vaccinated macaques, not only post-challenge DENV replication but also the changes in soluble immune mediators and hematological/biochemical parameters that are typically associated with dengue in humans.

## Results

### DPIV elicited long-lasting DENV-neutralizing antibody responses against the four DENV types

**Fig 1:**
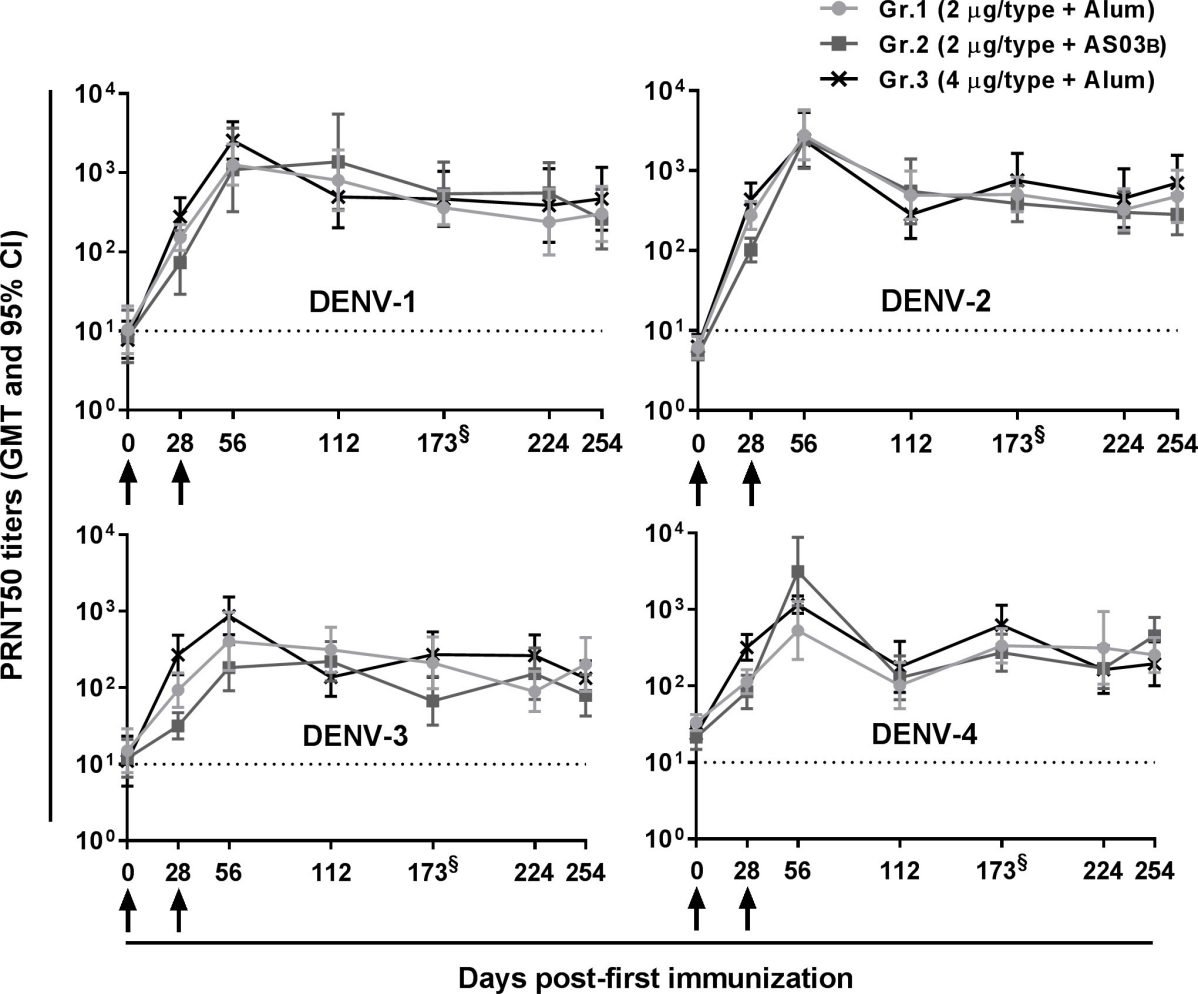
DPIV elicited broad and long-lasting DENV-nAb responses. Three groups of rhesus macaques received two intra-muscular administrations, 28 days apart, of the indicated formulations. Sera collected before and after immunization were tested, in duplicate, using a plaque reduction neutralization test (PRNT) for their neutralizing activity against each of the four DENV types. The individual reciprocal serum dilutions associated with 50% reduction in plaque counts (PRNT50 titers) were determined. Shown are the geometric mean titers (GMT) and 95% confidence intervals (CI) (n = 10/group except for Gr.3 at day 254, n = 9). Dotted lines indicate the limit of detection. ^§^Sera were collected in Gr.3 at day 168 instead of 173 in Gr.1-2.

Three groups of monkeys (Gr.1-3) received two doses four weeks apart of DPIV (2 or 4 μg/DENV type) adjuvanted with aluminum hydroxide (Alum) or the adjuvant system AS03_B_ (Table 1). The DENV-neutralizing antibody (DENV-nAb) titers, as measured in sera collected throughout the whole study, are shown in Fig 1. All tested vaccine formulations elicited DENV-nAb responses against the four DENV types that, at month 8 post-second immunization, were still detectable and did not significantly differ between groups (Fig 1 and S1–S3 Tables). As the measured DENV-nAb titers did mostly not differ between months 5 (day 168/173) and 8 (day 254) post-second immunization (S1 Table), we assumed the DENV-nAb response detected eight months following vaccination was representative of long-term immunity.

**Table 1:** Vaccine formulations and treatment groups.

Group	Number of animals	First vaccine dose (Day 0)^d^	Second vaccine dose (Day 28)^d^	DENV challenge	
				Time-point^e^	DENV strains
1	10	DPIV-2 μg+ Alum	DPIV-2 μg+ Alum	M8 pII	DENV-1 0111/2011 and DENV-2 0126/2010
2	10	DPIV-2 μg+ AS03_B_	DPIV-2 μg+ AS03_B_	M8 pII	DENV-1 0111/2011 and DENV-2 0126/2010
3^a^	9^c^	DPIV-4 μg+ Alum	DPIV-4 μg+ Alum	M8.5 pII	DENV-2 0126/2010 and DENV-2 S16803
4^b^	10	NA	NA	M8 pII	DENV-1 0111/2011 and DENV-2 0126/2010
5^b^	10	NA	NA	M8.5 pII	DENV-2 0126/2010 and DENV-2 S16803

^a^Group 3 (Gr.3) was included after Gr.1-2;

^b^Gr.4 and 5 were included, as non-vaccinated control groups, at the time of challenge of Gr.1-2 and Gr.3, respectively;

^c^One animal from Gr.3 died during the course of the experiment;

^d^Vaccine was administrated intra-muscularly;

^e^Gr.1, 2 and 4 and Gr.3 and 5 were subcutaneously challenged with the indicated DENV challenge strains at month 8 post-second immunization (M8 pII) and month 8.5 post-second immunization (M8.5 pII), respectively (n = 5/challenge subgroup but Gr.3 challenged with DENV-2 S16803, n = 4), NA, non applicable.

### DPIV failed at preventing DENV infection while signals of enhanced DENV replication were detected in a few vaccinated macaques

We next aimed at assessing DPIV efficacy to prevent post-challenge DENV replication. Characterization of dengue vaccine efficacy in macaques was previously performed by measuring, after DENV challenge, viremia [14,15,17] or both viremia and RNAemia, with viremia being the primary measure to conclude on vaccine efficacy [16,19]. However, we and others previously reported low or no detectable post-challenge DENV viremia in vaccinated macaques whereas RNAemia was detected at levels similar or higher than those detected in non-vaccinated monkeys. Although this discrepancy was explained by possible detection of viral RNA derived from degraded or neutralized DENV particles, it was not elucidated [16,19]. In our previous study, viremia had been measured using frozen-thawed sera [16]. As freeze-thawing of sera may reduce infectious titer of enveloped viruses-containing samples, here we assessed whether DENV viremia quantification was impacted by freeze-thawing of sera, while hypothesizing that RNAemia quantification might not be impacted.

**Fig 2:**
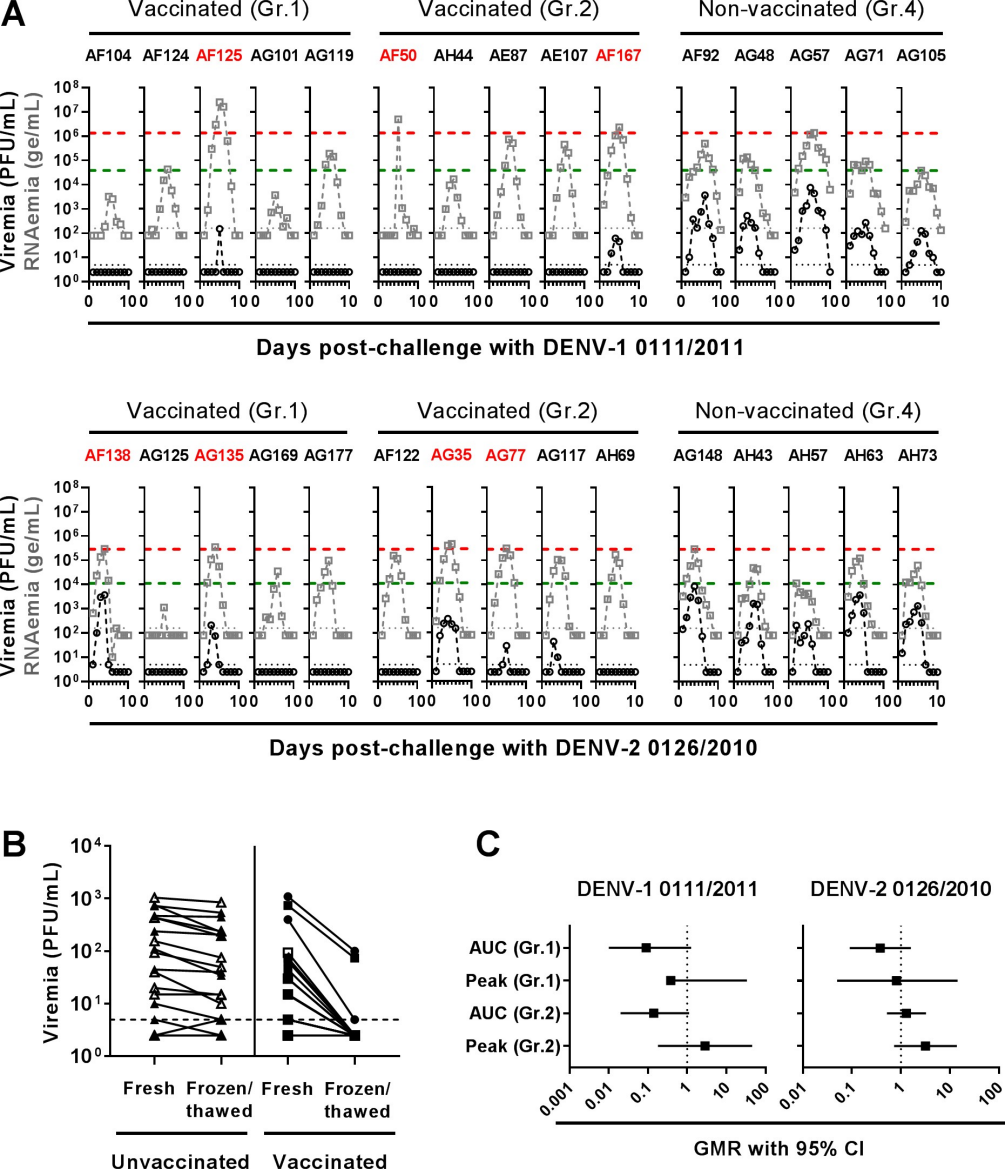
Viremia and RNAemia detected after challenge of Gr.1, 2 and 4 with either DENV-1 0111/2011 or DENV-2 0126/2010. At month 8 post-second immunization, Gr.1, 2 and 4 were divided into two subgroups each (n = 5) which were subcutaneously inoculated with approximately 10^5^ plaque-forming units (PFU) of either DENV-1 0111/2011 or DENV-2 0126/2010. (A) Shown are the individual viremia (expressed as plaque-forming units (PFU)/mL) and RNAemia (expressed as genome equivalent (ge)/mL) determined, using frozen-thawed sera, after inoculation with DENV-1 0111/2011 or DENV-2 0126/2010. Horizontal black and grey dotted lines indicate the threshold of detection for viremia and RNAemia, respectively. Horizontal green and red dashed lines indicate the lowest and highest RNAemia peaks detected in the corresponding non-vaccinated subgroup. Animals with RNAemia peaks higher than the highest peaks detected in non-vaccinated groups are indicated in red font. (B) Fresh sera collected at days 2 and 7 post-challenge were also tested for their viremia content, in parallel. Shown are the individual viremia titers determined using either fresh or frozen-thawed sera from days 2 and 7 post-challenge. Circle, square and triangle symbols correspond to values obtained with Gr.1, 2 and 4, respectively. Open and black symbols correspond to values obtained after challenge with DENV-1 0111/2011 and DENV-2 0126/2010, respectively. The horizontal dashed line indicates the threshold of detection for the plaque assay. (C) Shown are geometric mean ratio (GMR) and 95% confidence intervals (CI) for RNAemia area under the curves (AUC) and peak levels between each of the vaccinated groups and the non-vaccinated Gr.4. RNAemia AUC and peak levels were compared between vaccinated and non-vaccinated groups using an ANOVA model and a non-parametric analysis (ANOVA on ranks), respectively.

At month 8 post-second immunization, Gr.1, 2 and 4 (non-vaccinated control group) were each divided into two subgroups (n = 5) and challenged with either DENV-1 0111/2011 or DENV-2 0126/2010 (Table 1). These groups are further referred to as Gr.1, 2 or 4/DENV-1 0111/2011 and Gr.1, 2 or 4/DENV-2 0126/2010. When measured using frozen-thawed sera, all non-vaccinated macaques had detectable viremia whereas only 2 out of 10 and 5 out of 10 vaccinated monkeys had detectable viremia after challenge with DENV-1 0111/2011 and DENV-2 0126/2010, respectively (Fig 2A). However, viremia had also been measured, for two selected days, using fresh sera, and the titers determined on fresh *versus* frozen-thawed sera were compared (Fig 2B). Unexpectedly, freeze-thawing of sera did reduce viremia titration only in sera derived from vaccinated, but not non-vaccinated macaques, suggesting that comparing viremia titers between vaccinated and non-vaccinated animals could be biased when using frozen-thawed sera. We then focused on the RNAemia to further compare Gr.1-2 *versus* Gr.4. RNAemia was detected in all animals and, although the area under the curves (AUC) tended to be reduced in most vaccinated subgroups, the mean RNAemia peaks were 2.86- and 3.19-fold higher in Gr.2 compared to non-vaccinated Gr.4 after challenge with DENV-1 0111/2011 and DENV-2 0126/2010, respectively. Furthermore, 7 out of 20 vaccinated macaques showed higher RNAemia peaks (1.02- to 22-fold) compared to the highest peaks detected in the corresponding non-vaccinated subgroups (Fig 2A and 2C and S4 Table).

**Fig 3:**
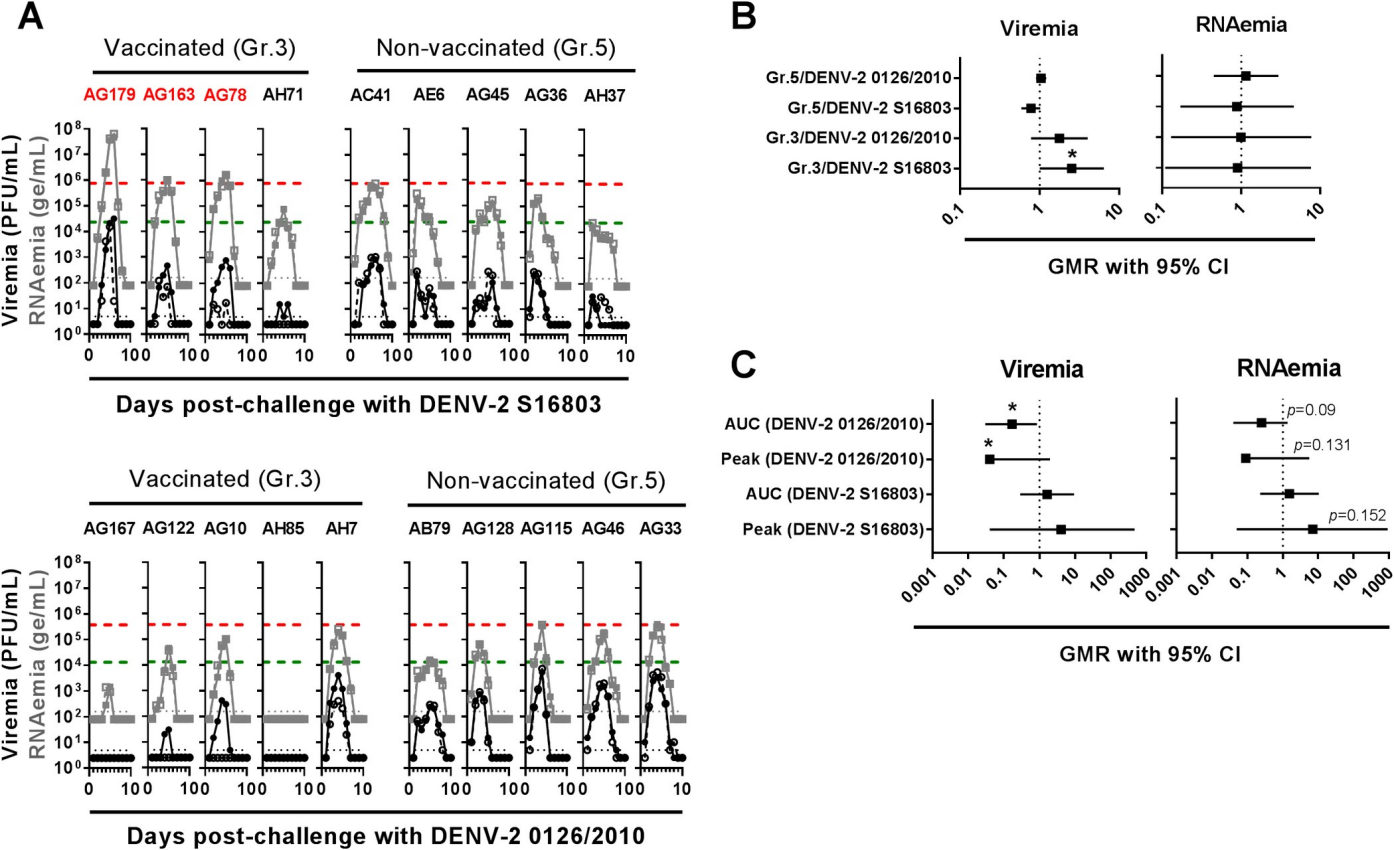
Viremia and RNAemia detected after challenge of Gr.3 and 5 with either DENV-2 0126/2010 or DENV-2 S16803. At month 8.5 post-second immunization, Gr.3 and 5 were divided into two subgroups each (n = 5 but Gr.3/DENV-2 S16803, n = 4) which were subcutaneously inoculated with approximately 4x10^4^ plaque-forming units (PFU) of DENV-2 S16803 or approximately 10^5^ PFU of DENV-2 0126/2010. (A) Shown are the individual viremia (expressed as plaque-forming units (PFU)/mL) and RNAemia (expressed as genome equivalent (ge)/mL) curves determined using either fresh (solid symbols) or frozen-thawed (open symbols) sera. Horizontal black and grey dotted lines indicate the threshold of detection for viremia and RNAemia, respectively. Horizontal green and red dashed lines indicate the lowest and highest RNAemia peaks detected in the corresponding non-vaccinated subgroup. Animals with RNAemia peaks higher than the highest peaks detected in non-vaccinated groups are indicated in red font. (B) Shown are the geometric mean ratio (GMR) and 95% confidence intervals (CI) for viremia and RNAemia area under the curves (AUC) determined using fresh *versus* frozen-thawed sera. Statistical comparisons were based on a paired t-test (*, *p*<0.05). (C) Shown are the GMR and 95% CI for viremia (determined using fresh sera) and RNAemia (determined using frozen-thawed sera) AUC and peak levels between the vaccinated Gr.3 and the non-vaccinated Gr.5. RNAemia AUC and peak levels were compared between vaccinated and non-vaccinated groups using an ANOVA model and a non-parametric analysis (ANOVA on ranks), respectively (both without adjusting for multiplicity; *, *p*<0.05).

To investigate whether the high-level RNAemia detected after challenge of Gr.1-2 was restricted to the newly selected isolates DENV-1 0111/2011 and DENV-2 0126/2010, Gr.3 and 5 were next divided into two subgroups and challenged, at month 8.5 post-second immunization, with either DENV-2 0126/2010 or the WHO reference DENV-2 strain S16803, which has frequently been used in rhesus macaques to assess efficacy of dengue vaccine candidates [15–17] (Table 1). These subgroups are further referred to as Gr.3 or 5/DENV-2 0126/2010 (n = 5) and Gr.3 or 5/DENV-2 S16803 (n = 4 and 5, respectively). To ensure accurate characterization of post-challenge DENV replication, viremia and RNAemia were measured using both fresh and frozen-thawed sera. Freeze-thawing of sera did not impact RNAemia quantification but, as previously observed (Fig 2B), did reduce viremia titration only in sera derived from vaccinated macaques (Fig 3A and 3B). For further analysis and after having confirmed the positive correlation between viremia and RNAemia values (S1 Fig), we focused on viremia and RNAemia titers in fresh and frozen-thawed sera, respectively. As shown in Fig 3A and 3C and in S5 and S6 Tables, after DENV-2 0126/2010 challenge, reduced viremia/RNAemia were detected in vaccinated compared to non-vaccinated macaques, with one animal (AH85) protected from viremia/RNAemia. In contrast, after DENV-2 S16803 challenge, viremia/RNAemia were not reduced, and the mean viremia and RNAemia peaks were 4.06- and 6.99-fold higher in Gr.3 compared to Gr.5, respectively. Importantly, 3 out of 4 vaccinated macaques showed higher RNAemia peaks (1.3- to 83.6-fold) compared to the highest peak detected in the non-vaccinated subgroup.

Altogether, DPIV vaccination failed to prevent post-challenge DENV replication, and was associated with increased RNAemia/viremia peaks in 10 out of 29 vaccinated macaques. While the vaccine failure to prevent DENV replication was further confirmed by the anamnestic responses detected approximately one month after challenge (S2 and S3 Figs), it could not be explained by a possible mismatch between the vaccine and challenge DENV strains as DPIV-elicited immunity neutralized all DENV challenge strains *in vitro* (S3 and S4 Figs).

### DPIV may have triggered ADE of DENV infection in some vaccinated macaques

**Fig 4:**
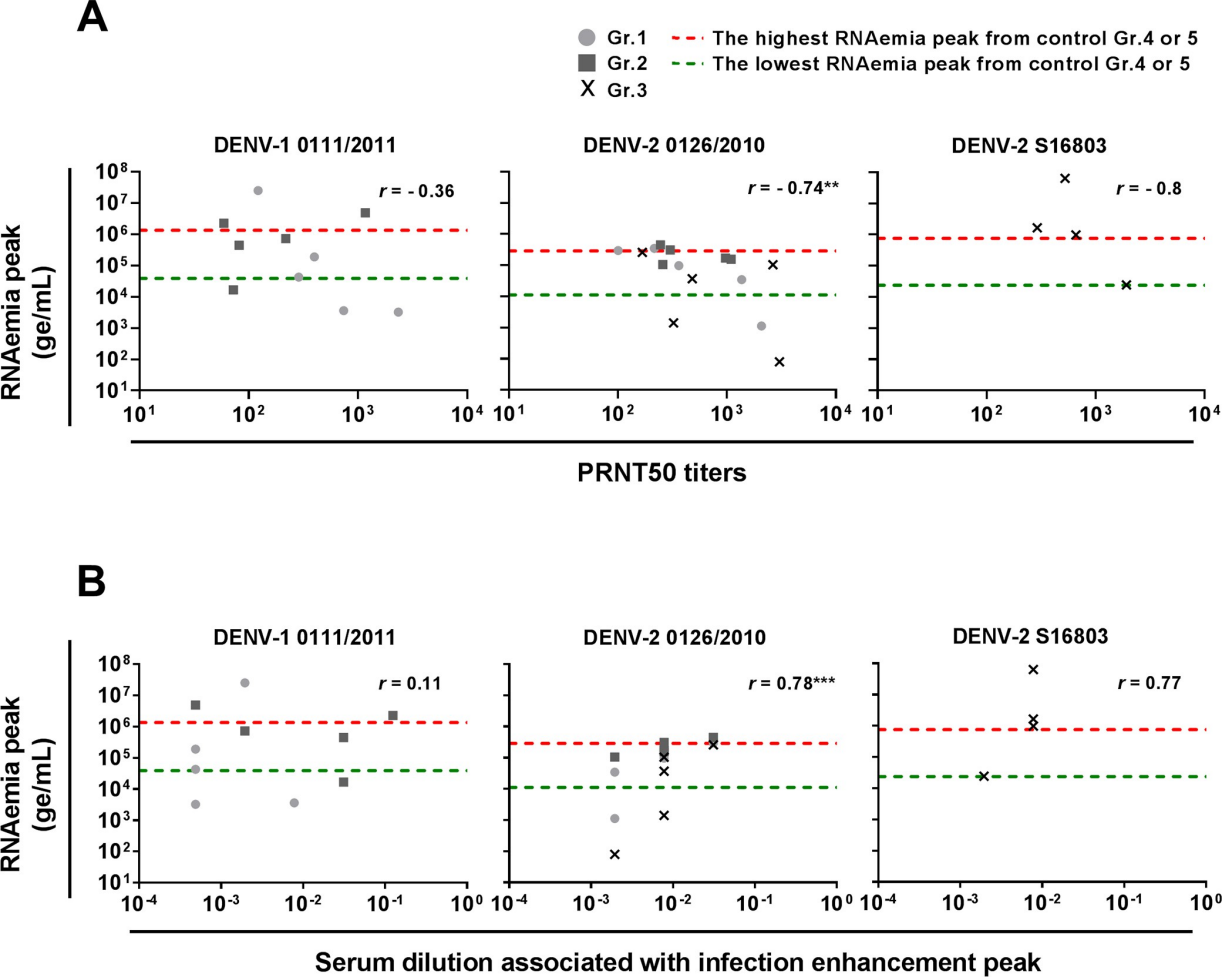
Correlation between pre-challenge DENV-nAb titers or serum DENV-enhancing capacities and post-challenge RNAemia peaks. Spearman correlations were performed to compare individual RNAemia peaks with pre-challenge (day 254) PRNT50 titers (A) or serum DENV-enhancing capacities (B), as measured, in a U937 cells-based antibody-dependent enhancement assay, by the serum dilution associated with infection enhancement peak, with the lowest and highest dilutions corresponding to the highest and lowest enhancing capacities, respectively (**, *p*<0.01; ***, *p*<0.001).

NAb responses are a major component of the DENV-specific immunity, with low and high pre-existing DENV-Ab/nAb titers correlating with severe dengue and, in contrast, with protection from dengue in humans, respectively [7,24,25]. Interestingly, negative correlations were observed between the pre-challenge DENV-nAb titers and the post-challenge RNAemia peaks (Fig 4A). This indicated an impact of the DENV-nAb response on the post-challenge RNAemia levels in DPIV-vaccinated macaques. Furthermore, the positive correlations detected, for the DENV-2 type, between the pre-challenge sera capacities at enhancing, *in vitro*, DENV infection and RNAemia peaks (Fig 4B) indicated that ADE of DENV infection may have occurred in some vaccinated macaques.

### Higher IFN-γ, IL-10 and IL-18, and lower IL-12 responses were detected after DENV challenge in vaccinated compared to non-vaccinated macaques

**Fig 5:**
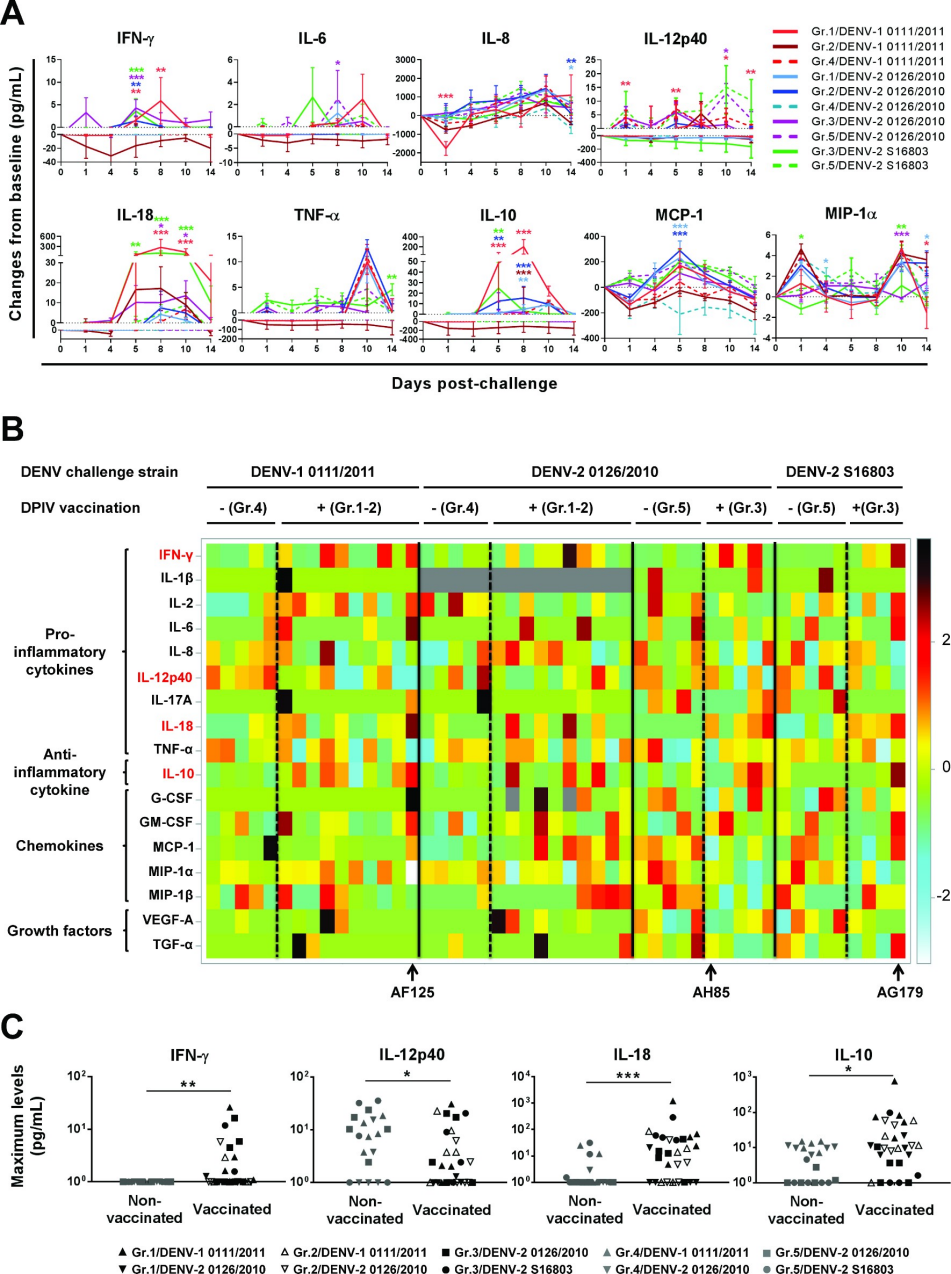
Post-challenge immune mediator profiles among vaccinated *versus* non-vaccinated macaques. Sera collected before and after DENV challenge were tested for their concentration in the indicated immune mediators. (A) Shown are the mean changes from baseline and SEM (n = 5 but Gr.3/DENV-2 S16803, n = 4). The log_10_-transformed changes from baseline were analyzed using an ANCOVA model. The *p*-values compare vaccinated to their corresponding non-vaccinated control subgroups with color codes referring to the vaccinated groups (*, *p*<0.05; **, *p*<0.01; ***, *p*<0.001). (B) Heat map representation of normalized scores of individual maximum changes from baseline in immune mediator levels. Monkeys were grouped by DENV challenge strain/wave, further divided based on their vaccination status, and ranked, within each subgroup, based on their maximum RNAemia level, monkeys with the lowest and the highest RNAemia peaks being on the left and the right sides, respectively. The immune mediators for which the maximum levels were further shown to significantly differ between vaccinated and non-vaccinated macaques are shown in red font. Also indicated are the macaques having shown more than 1-log higher RNAemia peaks compared to the highest RNAemia peaks detected in the corresponding non-vaccinated control groups (AF125 and AG179) as well as the only vaccinated macaque who was protected from post-challenge DENV replication (AH85). (C) A stratified non-parametric test was used to compare, across the DENV challenge strains/waves and between vaccinated and non-vaccinated macaques, the maximum immune mediator levels detected after DENV challenge. Shown are the individual values for the immune mediators for which the maximum levels significantly differed between vaccinated and non-vaccinated macaques (*, *p*<0.05; **, *p*<0.01; ***, *p*<0.001).

While ADE of DENV infection is proposed to be the early mechanism underlying DHF/DSS, one of the late mechanisms triggering the short-lived plasma leakage and coagulopathy, the hallmarks of DHF/DSS in humans, has been proposed to be the DENV infection-associated cytokine storm, including the pro-inflammatory cytokines interleukin (IL)-2, IL-6, IL-8, IL-12, IL-18, interferon (IFN)-γ and tumor necrosis factor (TNF)-α, the anti-inflammatory cytokine IL-10, the chemokines macrophage inflammatory protein (MIP)-1α, MIP-1β, and monocyte chemoattractant protein (MCP)-1, and the vascular endothelial growth factor (VEGF)-A [1,26–29]. In addition, we recently showed that infection of rhesus macaques with DENV-1 0111/2011 or DENV-2 0126/2010 was associated with modifications of the serum cytokine profile sharing similarities with those associated with dengue in humans, including post-infection increases in MCP-1 and IFN-γ levels [23]. Therefore, DPIV efficacy was further assessed here by characterizing the immune mediator profiles after DENV challenge. Sera collected before and after DENV challenge were tested for their concentrations in IFN-γ, IL-1β, IL-2, IL-6, IL-8, IL-10, IL-12/23, IL-17A, IL-18, MIP-1α, MIP-1β, TNF-α, TGF-α, VEGF, MCP-1, GM-CSF and G-CSF. The results are shown in Figs 5, S5 and S6. In all groups, levels of IL-1β, IL-2, IL-17A, GM-CSF, MIP-1β, TGF-α and VEGF-A were not or only slightly modified after challenge (S5 Fig). Increased G-CSF levels were detected only in Gr.3 and 5, without statistically significant between-group difference (S5 Fig). Increased MIP-1α levels were detected in all groups, with higher increases detected in non-vaccinated than in vaccinated macaques except for Gr.2/DENV-1 0111/2011 (Fig 5A). Consistent with dengue-associated cytokine profiles reported in humans, increased IL-6, IL-8, TNF-α and MCP-1 levels were detected in most groups after challenge. However, statistically significant were only the lower increase in IL-6 levels detected at day 8 in Gr.3/DENV-2 0126/2010, and the higher increases in MCP-1 levels detected at day 5 in Gr.1 and 2/DENV-2 0126/2010, compared to the control groups (Fig 5A). The most notable changes detected after challenge were related to IFN-γ, IL-10, IL-12 and IL-18. IL-10 and IL-18 levels were increased in all vaccinated groups and INF-γ levels were increased in all vaccinated groups but Gr.2/DENV-1 0111/2011, whereas these cytokines were not or minimally increased in non-vaccinated control groups (Fig 5A). In contrast, increased IL-12 levels were detected in all non-vaccinated groups but not, or only slightly, in vaccinated groups (Fig 5A). Furthermore, when comparing the maximum cytokine levels, irrespective of the vaccine formulation and the challenge strain, the peak levels of IFN-γ, IL-18 and IL-10 detected after challenge were significantly higher in vaccinated compared to non-vaccinated monkeys, whereas the peak levels of IL-12 were significantly lower in vaccinated compared to non-vaccinated groups (Fig 5B and 5C).

When further assessing the relationship between the maximum levels of IFN-γ, IL-12, IL-18 and IL-10 and the RNAemia peak levels, as shown in S6 Fig, both IL-10 and IL-18 levels were found to positively correlate with RNAemia peak levels (statistically significant for IL-10 and borderline significant for IL-18). Furthermore, the two vaccinated macaques that had shown the highest RNAemia peak increases compared to controls (AF125, 22-fold and AG179, 86.3-fold) also showed the highest increases in IFN-γ, IL-18, IL-10 levels, and the strongest down-regulation in IL-12 levels among all macaques. In contrast, the only vaccinated macaque that was fully protected from DENV replication (AH85) did not show any increase in IFN-γ and IL-10 levels but showed slightly increased IL-12 and IL-18 levels (Fig 5B). Altogether, these results showed that a lack of protection from DENV replication was associated with increased IFN-γ, IL-10 and IL-18 levels together with the absence/down-regulation of IL-12, all exacerbated in the case of increased RNAemia/viremia. In contrast, the combination of increased IL-12 and IL-18 levels, without increased IL-10 levels, might be associated with protection from DENV infection.

### Higher AST levels were detected after DENV challenge in vaccinated compared to non-vaccinated macaques

The main hematological and biochemical parameters monitored in clinical practice to predict dengue disease evolution are platelet and white blood cell (WBC) counts, hematocrit (HCT) and serum liver aminotransferases, both alanine and aspartate aminotransferases (ALT and AST), with thrombocytopenia, leukopenia, hematocrit increase >20% and elevated serum levels of AST/ALT being typical biological features of severe dengue in humans [30]. Therefore, hematological and biochemical parameters were characterized using blood samples collected before and at day 7 post-DENV challenge. The results are shown in S7 Fig. When comparing the changes from baseline, irrespective of the vaccine formulation and the DENV challenge strain, serum levels of AST were significantly higher in vaccinated compared to non-vaccinated macaques (S7B Fig). While no other statistically significant difference was detected, we cannot exclude that some differences might have been present at other time-points post-challenge.

## Discussion

To inform further clinical development, the DPIV vaccine candidate was assessed in rhesus macaques for its immunogenicity and efficacy to control DENV infection after a late challenge performed approximately eight months post-second immunization. While all tested vaccine formulations elicited long-lasting and cross-type DENV-nAb responses, they failed to prevent, not only post-challenge DENV replication, but also the biological changes that have been consistently associated with dengue severity in humans. In addition, increased DENV RNAemia/viremia, correlating with serum capacity at enhancing DENV infection *in vitro*, were observed in some vaccinated compared to non-vaccinated macaques, indicating the vaccine may have triggered ADE of DENV infection.

Serum/plasma DENV loads have been reported as positively correlating with dengue disease severity in humans [31–33], and so were DENV RNA levels [34,35]. Therefore, the non-reduced viremia/RNAemia observed after DPIV vaccination (except in Gr.3/DENV-2 0126/2010), and increased RNAemia peaks in some animals, might translate into lack of protection from disease and possibly enhanced disease in humans.

The post-challenge DENV replication was determined by measuring both viremia and RNAemia using either fresh or frozen-thawed sera. RNAemia quantification was not impacted by freeze-thawing of sera. In contrast and unexpectedly, the measured viremia titers were drastically lower when using frozen-thawed compared to fresh sera only for vaccinated but not non-vaccinated macaques. Although the limited remaining sera samples, together with the low viremia titers, prevented us from investigating the mechanism(s) underlying the lower detection of viremia in DPIV-immune frozen-thawed sera, we hypothesize that the viral particles may be captured within immune complexes that might be more prone to freeze-thawing-driven degradation, when compared to naked viral particles in sera from non-immunized animals. Importantly, if in the present study viremia had only been determined using frozen-thawed sera, and RNAemia had not been assessed, the wrong conclusion that DPIV immunization confers protection would have been made, and the enhanced DENV replication would have remained undetected. If, in previous dengue vaccine preclinical studies, viremia was assessed using frozen-thawed sera, post-challenge DENV replication was likely underestimated in vaccinated animals, and vaccine efficacy may have been overestimated. In addition, possible signals of vaccine-associated enhanced DENV replication would have been less likely detected. This might contribute to explain the discrepancy between the preclinical and clinical efficacy results reported for Dengvaxia [10,17,21,22]. Furthermore, beyond dengue vaccine studies in macaques and to avoid possible major biases in viral load quantification in preclinical/clinical/epidemiological studies, investigations should urgently be performed to determine whether such a phenomenon also occurs in humans (and in other animal models), and may be extended to other viruses/pathogens. In the meantime, our results strongly suggest that if measurement of viremia using fresh sera is not possible, measurement of viral genome should be favored.

IFN-γ, IL-10 and IL-18 have been frequently reported to be higher in the serum of DHF/DSS patients compared to DF patients and may thus be considered as immune markers of severe dengue in humans [27,29,32,36–45]. In contrast, IL-12 has been reported to be lower or not detectable in serum of DHF/DSS patients compared to DF patients [46,47], and its absence/reduced level may therefore be also considered as a marker of severe dengue.

IL-12 is a pro-inflammatory cytokine, mainly produced by phagocytic and antigen-presenting cells following exposure to intra-cellular pathogens including viruses. IL-12 plays a major role in early antiviral immunity through promoting IFN-γ production and thus Th1-type responses. In contrast, the absence/reduced levels of IL-12, driven by IL-10 that acts as the main inhibitor of IL-12, greatly contribute to skewing of the cytokine balance towards a Th2-biased response [48].

IL-18 is a pro-inflammatory cytokine, an important function of which is the induction, in concert with IL-12, of IFN-γ production [49,50]. However, in the absence of IL-12, IL-18 induces Th2-type cytokines and may therefore, depending upon the context, either promote Th1 or Th2 responses [49–51]. Interestingly, increased IL-18 levels together with decreased IL-12 levels were previously reported in human immunodeficiency virus (HIV)-infected patients to result in decreased IFN-γ production and Th2-biased immune responses, both being associated with increased HIV replication and development of acquired immunodeficiency syndrome [52]. Hence, it is likely that the combination of increased IL-18 levels and decreased IL-12 levels may also play an important role in dengue immunopathogenesis.

IFN-γ is mainly produced by activated T lymphocytes and NK cells and plays a crucial role in antiviral immunity, both through induction of effector molecules including nitric oxide and enhancement of antigen presentation and apoptosis [53]. The role of IFN-γ in DENV infection is controversial with several studies showing an association of elevated IFN-γ levels with DHF/DSS [36,39,42,54] whereas others showed that sustained IFN-γ production was associated with protection from dengue-associated clinical manifestations [55]. Although the peak levels of IFN-γ were significantly increased in vaccinated compared to non-vaccinated groups, only one group, *i*.*e*. Gr.3/DENV-2 0126/2010, showed sustained IFN-γ production after challenge (Fig 5A). As this group was also the one with better controlled DENV-replication (Fig 3A and 3C), our results corroborate the report by Gunther et al [55]. Given the non-reduced/enhanced DENV replication in the other vaccinated groups, we speculate that unlike sustained IFN-γ production, short-lasting IFN-γ production, albeit high, might fail to control DENV replication.

IL-10 is a Th2-type anti-inflammatory cytokine, mainly produced by monocytes/macrophages, type 2 T-helper and regulatory T cells, which acts as a major suppressor of antiviral immunity. Serum IL-10 level has been consistently reported as being drastically elevated in DHF/DSS patients, compared to DF patients, and is accepted as the strongest predictive immune marker of DHF/DSS [27,29,32,37,42,56,57]. In summary, an exacerbated IL-10 production may down-regulate IL-12, resulting, in the presence of increased IL-18 levels, in suppression of IFN-γ and thus in impaired antiviral immunity.

The positive correlations detected between the capacities of pre-challenge sera at enhancing DENV infection *in vitro* and the post-challenge RNAemia peaks indicated that ADE of DENV infection may have occurred in some vaccinated macaques. ADE of DENV production has been postulated to be mediated through promotion of virus entry into FcγR-bearing cells, resulting in an increased infected cell mass [3,4,58]. In addition, DENV infection via the FcγR-mediated pathway was shown, *in vitro*, to be associated with up-regulation of IL-10 expression and, in contrast, down-regulation of IL-12 expression, resulting in suppression of innate intra-cellular antiviral responses, and enhanced DENV replication. [3,59–63]. The combination of increased IL-10 and decreased IL-12 levels observed in some vaccinated macaques thus further supports that the vaccination may have triggered ADE of DENV infection.

Varying degrees of liver involvement have been reported in dengue patients and are believed to result from DENV-triggered hepatocyte apoptosis, hypoxic damage due to impaired liver perfusion associated with fluid leakage, oxidative stress and/or immune-mediated injury [64,65]. Elevation of liver aminotransferases is commonly reported during dengue in humans and serum ALT and AST levels are established biomarkers used to monitor dengue-related liver injury, with more frequent and higher AST level raise, when compared to ALT, making AST a more robust biomarker to monitor liver injury in dengue patients [30,66–68]. Furthermore, AST and ALT levels were consistently reported as being significantly higher in DHF/DSS patients compared to DF patients and are thus accepted as markers of disease severity [67,69,70].

Altogether, the higher AST, IL-10, IFN-γ, IL-18 levels and, in contrast, lower IL-12 levels detected after DENV challenge, in vaccinated compared to non-vaccinated macaques (Figs 5 and S7), reflect the overall biochemical/hematological and immune mediator profiles observed in humans experiencing severe dengue while further indicating that FcγR-mediated DENV infection may have occurred in vaccinated macaques.

In summary, although DPIV was immunogenic given as two doses four weeks apart, it failed to confer protection from DENV infection and may have triggered ADE of DENV infection. These results do not support further development of DPIV in humans. Despite the limitations of the dengue macaque model, we demonstrate here that, when assessing vaccine efficacy by performing a late DENV challenge, by accurately measuring post-challenge viral replication (both viremia and RNAemia, and viremia on fresh sera) and using a multiparameter approach, safety signals associated with a dengue vaccine candidate can be detected in this model. While there is room for further improvement of the dengue macaque model (for example by measuring the DENV infection-associated soluble nonstructural protein 1 which has been shown to directly trigger endothelial hyperpermeability and vascular leakage [71–73]), we believe the improved model described here may already be crucial to assess preclinical efficacy and safety of next-generation dengue vaccine candidates, and therefore de-risk large-scale human vaccination.

## Materials and methods

### Cell culture and viruses

Vero cells (ATCC No CCL-81) were grown at 37°C in a humidified 5%-CO_2_ incubator in medium 199 with Earle's salts supplemented with 5%-FBS (Gibco). U937 cells (ATCC No CRL-1593.2) were grown at 37°C in a humidified 5%-CO_2_ incubator in RPMI 1640 medium supplemented with 10%-FBS (Gibco). The DENV-1 0111/2011 and DENV-2 0126/2010 strains [23] were kindly provided by Dr Ana Bispo de Filippis (Instituto Oswaldo Cruz, Rio de Janeiro, Brazil), and used both for *in vivo* viral challenge and plaque-reduction neutralization test (PRNT). The DENV-1 WestPac-74 and DENV-2 S16803 strains were kindly provided by Dr Kenneth Eckels (Walter Reed Army Institute of Research, Silver Spring, USA), and used for *in vivo* viral challenge, PRNT, and ADE assay (DENV-2 S16803), and for ADE assay (DENV-1 WestPac-74). The DENV-1 60305 [74], DENV-2 44/2 [75], DENV-3 16562 [74] and DENV-4 TVP360 strains were used in PRNT.

### Vaccine formulations

DPIV antigen is based on a combination of attenuated viruses from all four DENV types amplified in Vero cells, purified by size-exclusion chromatography, and inactivated using both UV-treatment and formaldehyde. The four attenuated DENV strains used to produce DPIV have been previously described [76–79]. The same amount of antigen, either 2 or 4 μg, was used for each DENV type. The tetravalent DPIV antigen was then formulated with either Al(OH)_3_ or the GSK proprietary Adjuvant System AS03_B_ containing α-tocopherol and squalene as an oil in water emulsion.

### Ethics statement

The study protocol (protocol P-58/14-2 with license LW-49/14) was approved by the Institutional Ethical Committee for Use of Animals (CEUA-Fiocruz) and conducted in strict accordance with the recommendations from the Guide for Care and Use of Laboratory Animals of the Brazilian Society of Science in Laboratory Animals and the National Council for the Control of Animal Experimentation. The experiment was performed in a biohazard level 2 animal facility (temperature 20–22°C; humidity 50–60%; light/dark cycle 12 h/12 h). Monkeys were acclimated for 14 days before study start. Monkeys were housed individually but retained in a social environment through visual contact with other monkeys. Polished stainless-steel mirrors, PVC and wooden teethers, as well as foraging tray containing food such as pieces of cereal bars, raisins, rice grains or sunflower seeds were given as environmental enrichment. Monkeys had free access to water and received a commercial diet (Nuvilab Primates 6030 Nuvital) supplemented with fresh fruits and vegetables. Throughout the study, monkeys were observed twice a day by animal care and veterinary staff for health and well-being assessment. Monkeys were anesthetized with ketamine (8–10 mg/kg) prior to immunization, virus inoculation and blood drawing. At the end of the study, monkeys were anesthetized by intra-muscular injection of ketamine (20 mg/kg) prior to being euthanized by intra-peritoneal injection of thiopental sodium (50 mg/kg).

### Animals and experimental procedures

Fifty male or female adult rhesus macaques (*Macaca mulatta*) of Indian origin, 3-10-year-old and weighing 4.9–9.5 kg at the study start, flavivirus-naïve and colony-born in captivity in the Non-human Primates Breeding Service from the Institute of Science and Technology in Biomodels of the Oswaldo Cruz Foundation (Rio de Janeiro, Brazil), were used in this study. The 50 rhesus macaques were randomly allocated into five groups (n = 10/group) using the Sas Proc Optex software and taking into account the sex, the weight and the age. The male (M)/female (F) sex ratio was as follows: 7 M/3 F in Gr.1, 2 and 4, and 6 M/4 F in Gr.3 and 5. The sample size was determined based on 80% power analysis to detect a geometric mean ratio (GMR) of 3 for between-group geometric mean titer (GMT) comparisons, as well as to detect a GMR of 30 for between-group RNAemia area under the curves (AUC) comparisons while estimating, based on our previous similar experiment [16], at 0.38 and 0.75 the standard deviations for PRNT and qRT-PCR assays, respectively. Monkeys were anesthetized with ketamine (8–10 mg/kg) prior to immunization, virus inoculation and blood drawing. For vaccination, Gr.1, 2 and 3 received two intra-muscular injections of 0.5 mL of adjuvanted DPIV 28 days apart, as described in Table 1. Gr.4 and 5 were included as non-vaccinated control groups at the time of the DENV challenge of Gr.1-2 and 3, respectively. Blood samples were collected before each immunization, at days 56, 112, 168 or 173, 224 and 254 post-first immunization, as well as before DENV challenge, on a daily basis until at least day 12 post-challenge, and at day 39 post-challenge. Prior to DENV challenge, each group was subdivided into two groups into which macaques were randomly allocated using the same methodology than that described above. At month 8 post-second immunization, monkeys from Gr.1, 2 and 4 were subcutaneously challenged with 0.5 mL of sterile culture medium containing approximately 10^5^ plaque-forming units (PFU) of either DENV-1 0111/2011 (further referred to as Gr.1, 2 or 4/DENV-1 0111/2011 subgroups) or DENV-2 0126/2010 (further referred to as Gr.1, 2 or 4/DENV-2 0126/2010 subgroups). At month 8.5 post-second immunization, Gr.3 and 5 were subcutaneously challenged with 0.5 mL of sterile culture medium containing either approximately 10^5^ PFU of DENV-2 0126/2010 (further referred to as Gr.3 and 5/DENV-2 0126/2010 subgroups) or 4x10^4^ PFU of DENV-2 S16803 (further referred to as Gr.3 and 5/DENV-2 S16803 subgroups). The M/F sex ratio and sample size/subgroup were as follows: 3 M/2 F and n = 5 in Gr.1, 2 or 4/DENV-1 0111/2011, in Gr.3 or 5/DENV-2 0126/2010 and in Gr.5/DENV-2 S16803, 4 M/1 F and n = 5 in Gr.1, 2 or 4/DENV-2 0126/2010, and 3 M/1 F and n = 4 in Gr.3/DENV-2 S16803. After inoculation, titer of the residual viral inoculum was confirmed by plaque assay. One monkey from Gr.3 became ill and died before the end of the study (at month 5 post-second immunization). When performing post-mortem histological analysis, this macaque was diagnosed with hydrocephalic brain. No additional microscopic lesion was observed in both the central nervous system and the extraneural organs. Although the hydrocephalus etiology was not determined, we hypothesized it to be congenital and most likely not related to the DPIV vaccination.

### Plaque-reduction neutralization test (PRNT)

The PRNTs were performed using different DENV strains when compared to those DPIV is derived from. The following strains were used: DENV-1 60305, DENV-2 44/2, DENV-3 16562 and DENV-4 TVP360 or the DENV challenge strains (DENV-1 0111/2011, DENV-2 0126/2010 and DENV-2 S16803). One hundred DENV PFU were mixed with equal volumes of serially diluted sera and incubated for 1 h at 37°C. The mixtures were added for 1 h at 37°C onto Vero cells previously seeded into 6-well plates, and subsequently replaced by maintenance medium supplemented with 2%-carboxyl-methyl-cellulose (CMC). Seven (DENV-2) or eight (DENV-1, DENV-3 and DENV-4) days later, cells were fixed overnight with 5%-formaldehyde solution prior to crystal violet staining. PFU were counted by the naked eye and the percent neutralization was determined relative to the number of PFU counted with the virus control (corresponding to 0% neutralization). PRNT50 titers, corresponding to the reciprocal serum dilution associated with 50% reduction in plaque counts, were determined using a linear model. All serum samples were tested in duplicate. In the absence of detection of neutralizing activity, the corresponding sample was assigned an arbitrary titer corresponding to half the limit of detection, namely 5.

### Antibody-dependent enhancement (ADE) assay

Heat-inactivated macaque sera, collected on day 254 (before challenge), were diluted 4-fold starting from 1:2 prior to being incubated for 2 h at 37°C with either DENV-1 WestPac-74 (MOI = 1) or DENV-2 S16803 (MOI = 10) in maintenance medium without FBS. As negative controls for DENV infection enhancement, viruses were mixed with medium only or with sera derived from DENV/DPIV-naïve macaques (from Gr.4 and 5). A total of 5x10^4^ U937 cells were then added to each well containing the sera/virus mixtures, and incubated for 1 h at 37°C. The medium was next removed and replaced by maintenance medium with 10%-FBS, and the cells were incubated for 48 h at 37°C. The cells were then harvested, washed and fixed/permeabilized (BD Cytofix/Cytoperm, BD Biosciences) prior to intracellular staining for the DENV envelop using the pan-flavivirus 4G2 mouse monoclonal antibody (8 μg/mL) followed by 0.7 μg/mL phycoerythrin-conjugated rat anti-mouse IgG2a (Biolegend). The proportion of infected cells was then determined by flow-cytometry (the data were acquired using either FACS LSRII or Fortessa, and analyzed using the FlowJo 9.9.6 software, all from BD Biosciences). DENV infection enhancement was determined relative to the infection percent detected with virus alone (without serum). The cut-off for DENV infection enhancement was set at 2.5-fold infection increase compared to virus alone.

### Virus titration by plaque assay

Serial dilutions of macaque sera were added onto Vero cells previously seeded into 6-well plates. After 1 h at 37°C, the diluted samples were replaced by maintenance medium supplemented with 2%-CMC. Seven days later cells were fixed overnight with a 5%-formaldehyde solution prior to crystal violet staining. PFUs were counted by the naked eye and infectious virus titers were expressed as PFU/mL. All serum samples were tested in duplicate. In the absence of PFU detection, the corresponding sample was assigned an arbitrary titer corresponding to half the limit of detection, namely 2.5 PFU/mL.

### DENV genome equivalents quantification by real-time RT-PCR

Viral RNA was extracted from 200 μL of monkey sera using the LSI MagVet Universal Isolation Kit (YSI-Thermo Fisher Scientific). DENV genome equivalents were quantitated by real-time RT-PCR using the AgPath-ID One-Step RT-PCR kit (Ambion). Each RT-PCR reaction mixture contained 2.5 μL of RNA, 1.67 μL of Detection Enhancer, 2X RT-PCR Buffer, 25X RT-PCR Enzyme Mix (all from the kit), 20 U of RNAsin (Ambion), as well as 10 and 5 pmol of DENV type-specific forward/reverse primers and probes, respectively. Primers and probes used were: DENV-1, forward 5’-GCA-TTY-CTA-AGA-TTT-CTA-GCC-ATA-CC-3’, reverse 5’-TCG-CTC-CAT-TCT-TCT-TGA-ATG-AG-3’, probe 5’-AAC-AGC-AGG-AAT-TTT-3’, and DENV-2, forward 5’-CTG-CAR-GGA-CGA-GGA-CCA-TT-3’, reverse 5’-GGG-ATT-GTT-AGG-AAA-CGA-AGG-A-3’, probe 5’-AAA-CTG-TTC-ATG-GCC-CTG-GTG-GCR-3’. RT was performed using an ABI 7500 Real-Time PCR system (Applied Biosystems) at 45°C for 10 min, followed by an incubation step at 95°C for 10 min and 40 cycles of 15 s at 95°C and 1 min at 60°C. All serum samples were tested in duplicate. In the absence of RNAemia detection, the corresponding sample was assigned an arbitrary titer corresponding to half the limit of detection, namely 80 ge/mL.

### Cytokine quantification

The seventeen following cytokines were assessed: IFN-γ, IL-1β, IL-2, IL-6, IL-8, IL-12/23, IL-17A, IL-18, TNF-α, IL-10, G-CSF, GM-CSF, MCP-1, MIP-1α, MIP-1β, TGF-α, and VEGF-A. Undiluted sera were tested in duplicate using the MILLIPLEX MAP Non-Human Primate Cytokines Magnetic Bead Panel Kit (Merck). Data were acquired and analyzed using the Luminex 200 reader and the BioPlex Manager software (BioRad). Results were expressed as pg/mL and log_10_-transformed for statistical analysis. In the case of a cytokine level ≤ 1, the level was arbitrary set at 1 prior to log_10_-transformation for statistical analysis. When no signal was detected, the corresponding sample was assigned the arbitrary value of half the limit of detection for the corresponding cytokine. The changes from baseline in cytokine concentrations were analyzed in an ANCOVA model with group, time and group-by-time interaction as factors and baseline value as covariate, the structure of correlation between time-points being of the Toeplitz type. For each of the four DENV challenge waves (Gr.1, 2 and 4 challenged with DENV-1 0111/2011, Gr. 1, 2 and 4 challenged with DENV-2 0126/2010, Gr.3 and 5 challenged with DENV-2 0126/2010 and Gr.3 and 5 challenged with DENV-2 S16803), a non-parametric test was used to compare the maximum change from baseline in cytokine levels between vaccinated *versus* non-vaccinated macaques. For heat map representation, normalized scores for maximum change from baseline in cytokine levels were determined by challenge wave.

### Laboratory testing for hematological and biochemical parameters

Hematological analyses were performed using the automatic counter Sysmex pocH Diff (Roche Diagnóstica Brasil Ltda, São Paulo, Brazil). Biochemical analyses were performed using the auto-analyses system Vitros 250 XRC (Johnson–Johnson Clinical Diagnostics, Rochester, NY). The following parameters were evaluated: red and white blood cell counts (RBC and WBC), alanine aminotransferase (ALT), aspartate aminotransferase (AST) and creatinine (CREA). Baseline levels were determined from blood samples collected before DENV challenge.

### Statistical analysis

All statistical analysis methods are described in the corresponding method sections and/or figure and table legends.

## Supporting information

S1 Fig Correlations between viremia and RNAemia. Black square and circle symbols correspond to values obtained from Gr.3 after challenge with DENV-2 S16803 and DENV-2 0126/2010, respectively. Grey square and circle symbols correspond to values obtained from Gr.5 after challenge with DENV-2 S16803 and DENV-2 0126/2010, respectively. Pearson and Spearman correlations were performed to compare AUC and peak levels, respectively (***, *p*<0.001).
(TIF)

S2 Fig DENV-neutralizing antibody responses as determined before and after DENV challenge. Sera collected before challenge (on day 254 post-first immunization) and 39 days after challenge (day 309 and 321 for Gr.1, 2 and 4 and Gr.3 and 5, respectively) were tested, in duplicate, in plaque reduction neutralization test (PRNT) for their neutralizing activity against each of the four DENV serotypes. The DENV challenge strains are indicated above graphs. The individual reciprocal serum dilutions associated with 50% reduction in plaque counts (PRNT50 titers) were determined. The geometric mean titers (GMT) and 95% confidence intervals (CI) are shown for each of the three vaccinated groups (n = 5/group except for Gr.3/DENV-2 S16803 for which n = 4).
(TIF)

S3 Fig Neutralizing antibody responses against DENV challenge strains as determined before and after challenge. Sera collected before challenge (on day 254 post-first immunization) and 39 days after challenge (day 309 and 321 for Gr.1, 2 and 4 and Gr.3 and 5, respectively) were tested, in duplicate, in plaque reduction neutralization test (PRNT) for their neutralizing activity against each of the DENV challenge strains. The DENV strains used to challenge the different subgroups are indicated above graphs. The individual reciprocal serum dilutions associated with 50% reduction in plaque counts (PRNT50 titers) were determined. The geometric mean titers (GMT) and 95% confidence intervals (CI) are shown (n = 5/subgroup except for Gr.3/DENV-2 S16803 for which n = 4).
(TIF)

S4 Fig DPIV-elicited antibody responses potently neutralized *in vitro* the three DENV challenge strains. Sera collected before immunization and at days 56 and 254 post-first immunization were tested, in duplicate, in plaque reduction neutralization test (PRNT) for their neutralizing activity against the DENV challenge strains, which are indicated above graphs. The individual reciprocal serum dilutions associated with 50% reduction in plaque counts (PRNT50 titers) were determined. The geometric mean titers (GMT) and 95% confidence intervals (CI) are shown for each of the three vaccinated groups (n = 5/group except for Gr.3/DENV-2 S16803 at day 254 for which n = 4).
(TIF)

S5 Fig Serum levels in IL-1β, IL-2, IL-17, MIP-1β, G-CSF, GM-CSF, TGF-α and VEGF-A were not or slightly modified after challenge with DENV-1 0111/2011, DENV-2 0126/2010 or DENV-2 S16803. Sera collected before (baseline) and at days 1, 4, 6, 8, 10 and 14 after challenge were tested, in duplicate, for their concentration in the indicated soluble mediators. Results were expressed as pg/mL. When no signal was detected, the corresponding sample was assigned the arbitrary value of half the limit of detection for the corresponding mediator. Shown are the mean changes from baseline and SEM from 5 (Gr.1, 2, 4/DENV-1 0111/2011, Gr.1-5/DENV-2 0126/2010, Gr.5/DENV-2 S16803) and 4 (Gr.3/DENV-2 S16803) animals. For statistical analysis, the log_10_-transformed changes from baseline were analyzed using an ANCOVA model with group, time and group-by-time interaction as factors and baseline values as covariates. The calculated *p*-values compare, by DENV challenge strain, vaccinated groups to their corresponding non-vaccinated control groups with color codes referring to the vaccinated groups (*, *p*<0.05; **, *p*<0.01; ***, *p*<0.001).
(TIF)

S6 Fig Relationship between RNAemia peaks and maximum changes from baseline in IFN-γ, IL-10, IL-12 and IL-18 levels. The relationship between the RNAemia peaks and the maximum changes from baseline in IFN-γ, IL-10, IL-12 and IL-18 levels was assessed using a linear regression model performed on log_10_-transformed values. Shown are all individual values together with, for the vaccinated groups, the linear regression lines. The statistical significance of the linear regression slopes to be different from 0 was assessed across the different vaccinated groups/challenge waves. The measured *p* values are indicated. No *p* value could be calculated for IFN-γ due to inter-group interference.
(TIF)

S7 Fig Post-challenge changes from baseline in hematological and biochemical parameters among vaccinated *versus* non-vaccinated macaques. Whole anticoagulated venous blood samples, collected before (baseline) and at day 7 post-DENV challenge, were tested for the indicated hematological and biochemical parameters (ALT, alanine aminotransferase; AST, aspartate aminotransferase; GGT, gamma glutamyl transferase; HCT, hematocrit; WBC, white blood cells; MCH, mean corpuscular hemoglobin; MCHC, mean corpuscular hemoglobin concentration; MCV, mean corpuscular volume). (A) Heat map representation of normalized scores of individual changes from baseline. Monkeys were grouped by DENV challenge strain/wave, further divided based on their vaccination status, and ranked, within each subgroup, based on their maximum RNAemia level, monkeys with the lowest and the highest RNAemia peaks being on the left and the right sides, respectively. Score normalization was performed by DENV challenge strain/wave so that normalized scores can only be compared between vaccinated and non-vaccinated macaques within each DENV challenge strain/wave. The only parameter for which the change from baseline was further shown to significantly differ between vaccinated and non-vaccinated macaques is shown in red font. (B) An ANOVA model was used to compare, across the DENV challenge strains/waves, the changes from baseline in hematological/biochemical parameters between vaccinated and non-vaccinated macaques. Shown are the individual values for AST (*, *p*<0.05).
(TIF)

S1 Table Between-time-point PRNT50 comparisons. (DOCX)

S2 Table Between-DENV type PRNT50 comparisons. (DOCX)

S3 Table Between-group PRNT50 comparisons. (DOCX)

S4 Table RNAemia area under the curves, peaks and durations after challenge of Gr.1-2 and Gr.4 with either DENV-1 0111/2011 or DENV-2 0126/2010 (frozen-thawed sera). (DOCX)

S5 Table Viremia area under the curves, peaks and durations after challenge of Gr.3 and Gr.5 with either DENV-2 0126/2010 or DENV-2 S16803 (fresh sera). (DOCX)

S6 Table RNAemia area under the curves, peaks and durations after challenge of Gr.3 and Gr.5 with either DENV-2 0126/2010 or DENV-2 S16803 (frozen-thawed sera). (DOCX)

S1 Data Hematology. (DOCX)

S2 Data Immune mediators. (XLSX)

S3 Data Individual PRNT50. (XLSX)

S4 Data Individual PRNT50 against challenge DENV strains. (XLSX)

S5 Data Viremia_Gr1_Gr2_Gr4. (XLSX)

S6 Data Viremia_Gr3_Gr5. (XLSX)

S7 Data RNAemia_Gr1_Gr2_Gr4. (XLSX)

S8 Data RNaemia_Gr3_Gr5. (XLSX)

S9 Data ADE. (XLSX)
