# Supplementary figures and images for: Detection of post-vaccination enhanced dengue virus infection in macaques: An improved model for early assessment of dengue vaccines
Source: PLoS Pathog. 2019 Apr 22;15(4):e1007721. doi: 10.1371/journal.ppat.1007721 (PMC6497418; doi:10.1371/journal.ppat.1007721)

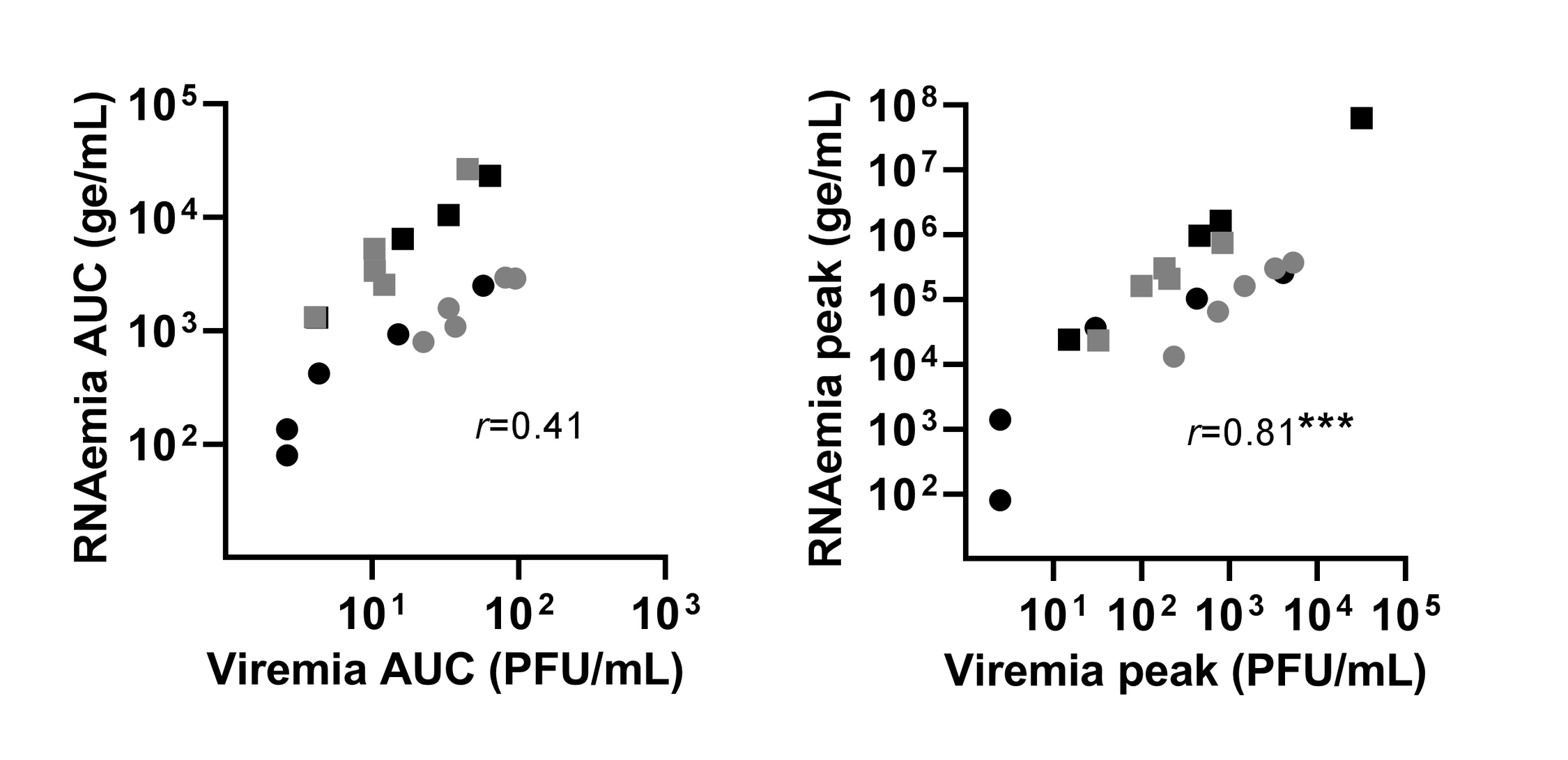

Supplement: S1 Fig — Black square and circle symbols correspond to values obtained from Gr.3 after challenge with DENV-2 S16803 and DENV-2 0126/2010, respectively. Grey square and circle symbols correspond to values obtained from Gr.5 after challenge with DENV-2 S16803 and DENV-2 0126/2010, respectively. Pearson and Spearman correlations were performed to compare AUC and peak levels, respectively (***, p<0.001). (TIF) [file ppat.1007721.s001.tif]

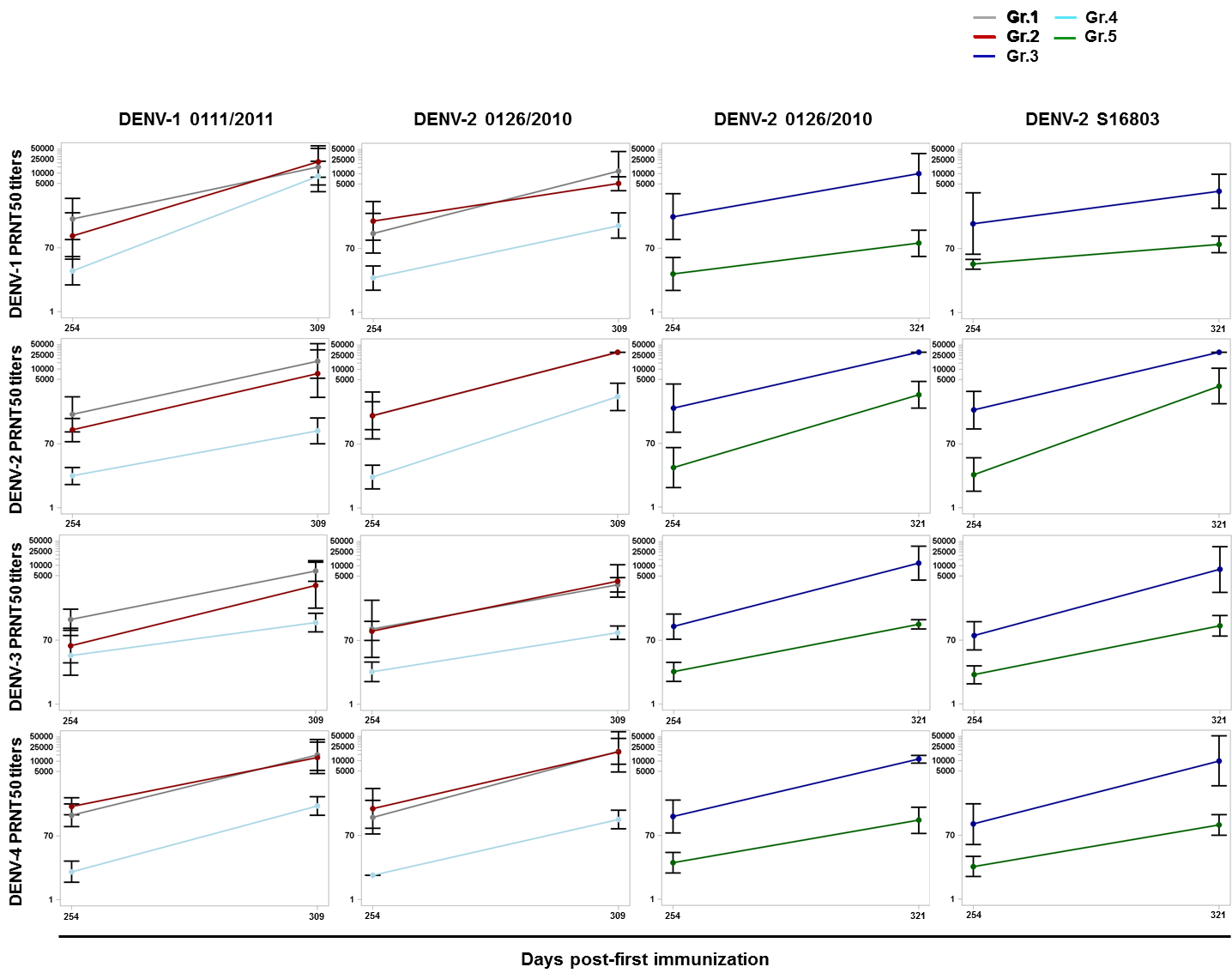

Supplement: S2 Fig — Sera collected before challenge (on day 254 post-first immunization) and 39 days after challenge (day 309 and 321 for Gr.1, 2 and 4 and Gr.3 and 5, respectively) were tested, in duplicate, in plaque reduction neutralization test (PRNT) for their neutralizing activity against each of the four DENV serotypes. The DENV challenge strains are indicated above graphs. The individual reciprocal serum dilutions associated with 50% reduction in plaque counts (PRNT50 titers) were determined. The geometric mean titers (GMT) and 95% confidence intervals (CI) are shown for each of the three vaccinated groups (n = 5/group except for Gr.3/DENV-2 S16803 for which n = 4). (TIF) [file ppat.1007721.s002.tif]

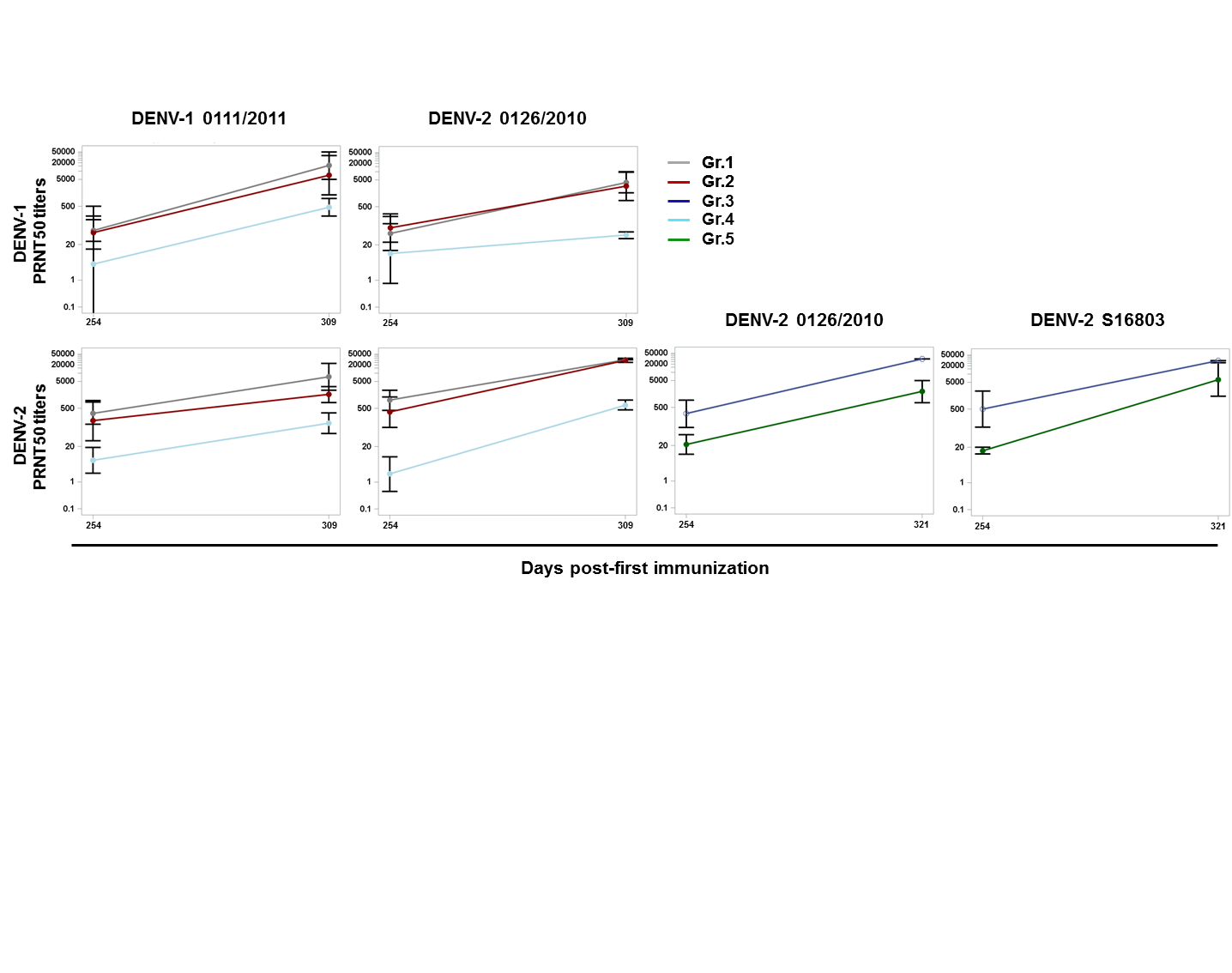

Supplement: S3 Fig — Sera collected before challenge (on day 254 post-first immunization) and 39 days after challenge (day 309 and 321 for Gr.1, 2 and 4 and Gr.3 and 5, respectively) were tested, in duplicate, in plaque reduction neutralization test (PRNT) for their neutralizing activity against each of the DENV challenge strains. The DENV strains used to challenge the different subgroups are indicated above graphs. The individual reciprocal serum dilutions associated with 50% reduction in plaque counts (PRNT50 titers) were determined. The geometric mean titers (GMT) and 95% confidence intervals (CI) are shown (n = 5/subgroup except for Gr.3/DENV-2 S16803 for which n = 4). (TIF) [file ppat.1007721.s003.tif]

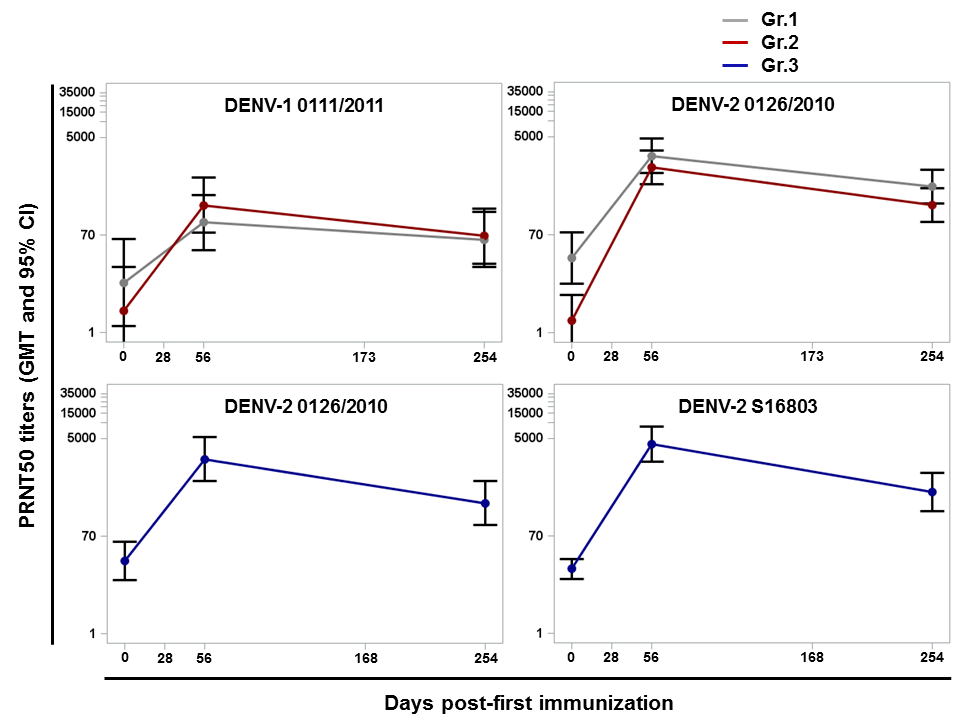

Supplement: S4 Fig — Sera collected before immunization and at days 56 and 254 post-first immunization were tested, in duplicate, in plaque reduction neutralization test (PRNT) for their neutralizing activity against the DENV challenge strains, which are indicated above graphs. The individual reciprocal serum dilutions associated with 50% reduction in plaque counts (PRNT50 titers) were determined. The geometric mean titers (GMT) and 95% confidence intervals (CI) are shown for each of the three vaccinated groups (n = 5/group except for Gr.3/DENV-2 S16803 at day 254 for which n = 4). (TIF) [file ppat.1007721.s004.tif]

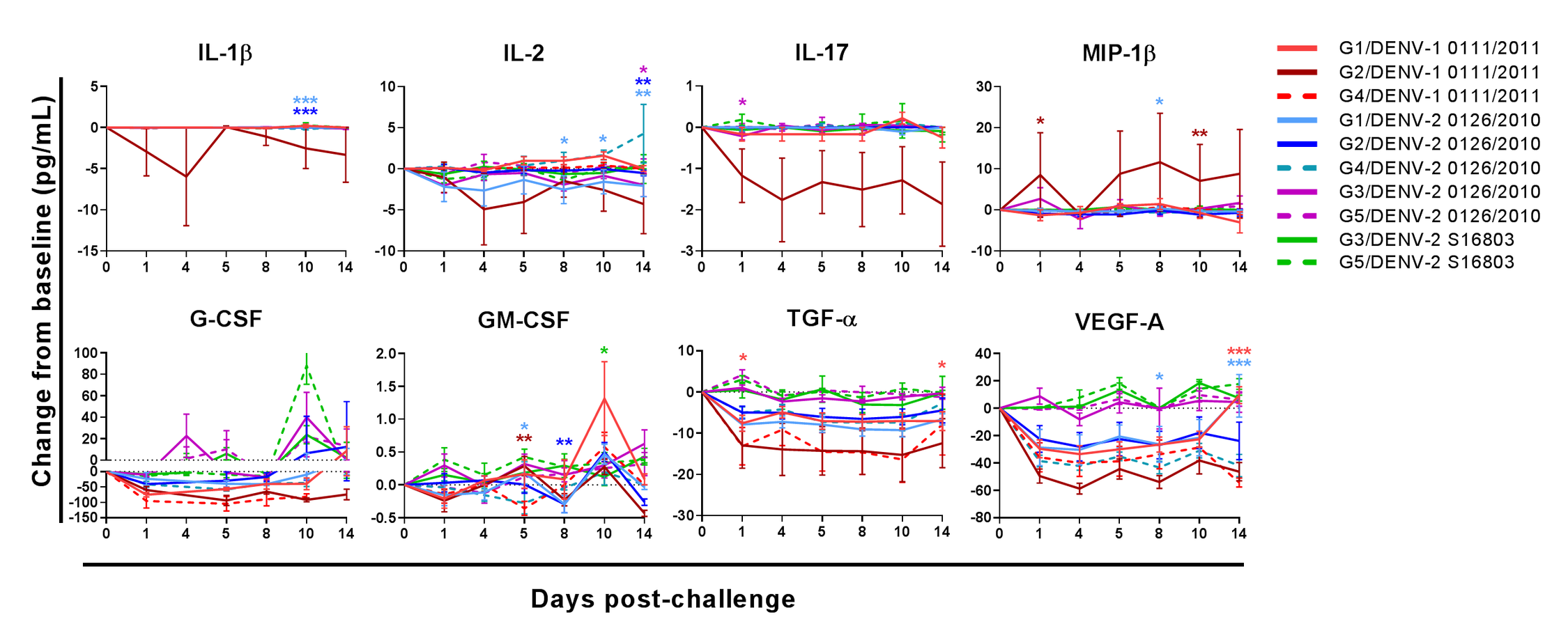

Supplement: S5 Fig — Sera collected before (baseline) and at days 1, 4, 6, 8, 10 and 14 after challenge were tested, in duplicate, for their concentration in the indicated soluble mediators. Results were expressed as pg/mL. When no signal was detected, the corresponding sample was assigned the arbitrary value of half the limit of detection for the corresponding mediator. Shown are the mean changes from baseline and SEM from 5 (Gr.1, 2, 4/DENV-1 0111/2011, Gr.1-5/DENV-2 0126/2010, Gr.5/DENV-2 S16803) and 4 (Gr.3/DENV-2 S16803) animals. For statistical analysis, the log10-transformed changes from baseline were analyzed using an ANCOVA model with group, time and group-by-time interaction as factors and baseline values as covariates. The calculated p-values compare, by DENV challenge strain, vaccinated groups to their corresponding non-vaccinated control groups with color codes referring to the vaccinated groups (*, p<0.05; **, p<0.01; ***, p<0.001). (TIF) [file ppat.1007721.s005.tif]

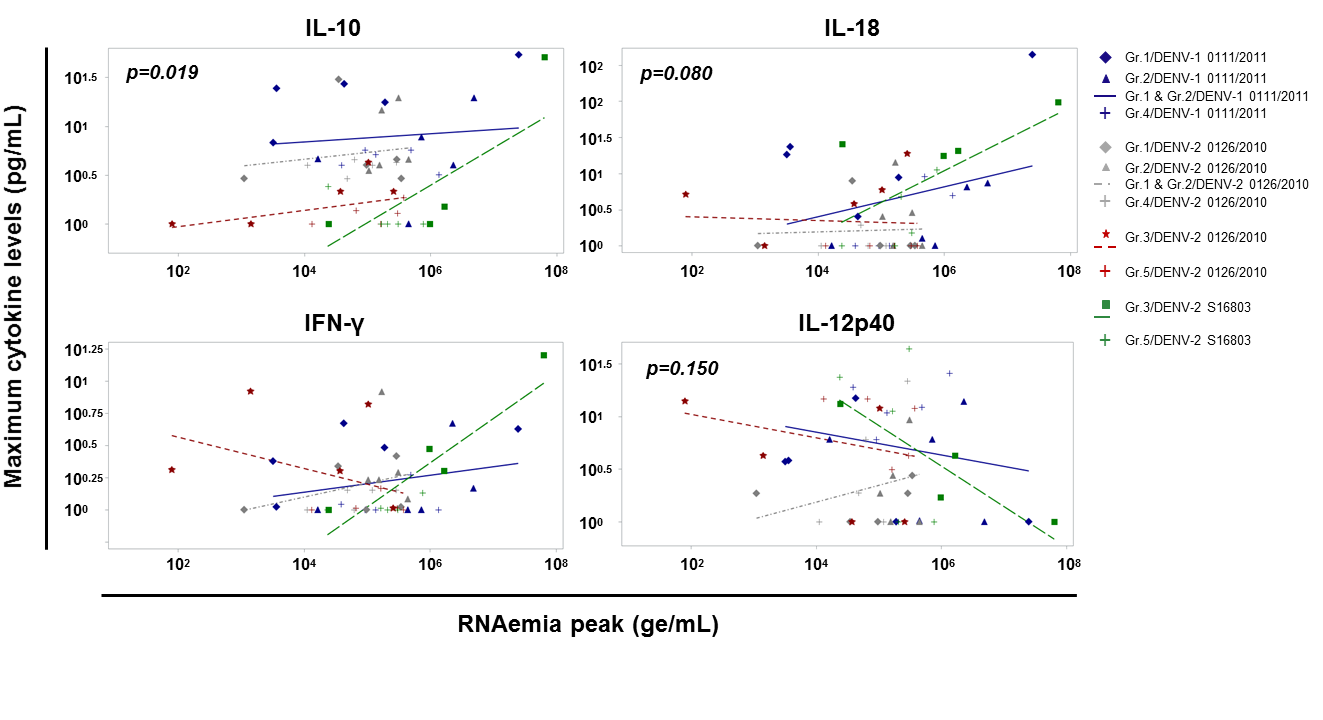

Supplement: S6 Fig — The relationship between the RNAemia peaks and the maximum changes from baseline in IFN-γ, IL-10, IL-12 and IL-18 levels was assessed using a linear regression model performed on log10-transformed values. Shown are all individual values together with, for the vaccinated groups, the linear regression lines. The statistical significance of the linear regression slopes to be different from 0 was assessed across the different vaccinated groups/challenge waves. The measured p values are indicated. No p value could be calculated for IFN-γ due to inter-group interference. (TIF) [file ppat.1007721.s006.tif]

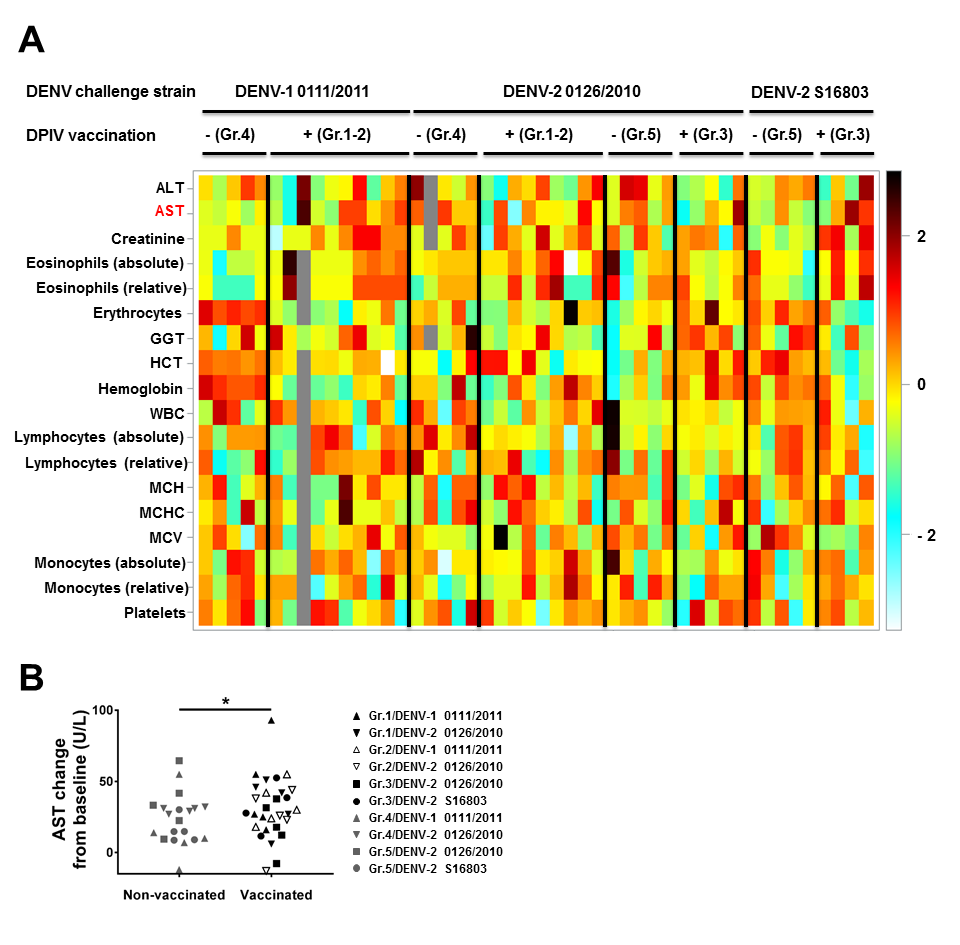

Supplement: S7 Fig — Whole anticoagulated venous blood samples, collected before (baseline) and at day 7 post-DENV challenge, were tested for the indicated hematological and biochemical parameters (ALT, alanine aminotransferase; AST, aspartate aminotransferase; GGT, gamma glutamyl transferase; HCT, hematocrit; WBC, white blood cells; MCH, mean corpuscular hemoglobin; MCHC, mean corpuscular hemoglobin concentration; MCV, mean corpuscular volume). (A) Heat map representation of normalized scores of individual changes from baseline. Monkeys were grouped by DENV challenge strain/wave, further divided based on their vaccination status, and ranked, within each subgroup, based on their maximum RNAemia level, monkeys with the lowest and the highest RNAemia peaks being on the left and the right sides, respectively. Score normalization was performed by DENV challenge strain/wave so that normalized scores can only be compared between vaccinated and non-vaccinated macaques within each DENV challenge strain/wave. The only parameter for which the change from baseline was further shown to significantly differ between vaccinated and non-vaccinated macaques is shown in red font. (B) An ANOVA model was used to compare, across the DENV challenge strains/waves, the changes from baseline in hematological/biochemical parameters between vaccinated and non-vaccinated macaques. Shown are the individual values for AST (*, p<0.05). (TIF) [file ppat.1007721.s007.tif]
