# Supplementary material for: Detection of post-vaccination enhanced dengue virus infection in macaques: An improved model for early assessment of dengue vaccines
Source: PLoS Pathog. 2019 Apr 22;15(4):e1007721. doi: 10.1371/journal.ppat.1007721 (PMC6497418; doi:10.1371/journal.ppat.1007721)
Supplement: S1 Table — (DOCX) [file ppat.1007721.s008.docx]

**S1 Table. Between-time-point PRNT50 comparisons.**

| **DENV type** | **Group** | **Time-point 1** | **Time-point 2** | **GMR^a^** | **Lower Limit^b^** | **Upper Limit^b^** | **Significance^c^** |
| --- | --- | --- | --- | --- | --- | --- | --- |
| **DENV-1** | Gr.1 | 28 | 56 | 8.34 | 4.59 | 15.13 | *** |
|  | Gr.1 | 56 | 112 | 0.63 | 0.35 | 1.15 | ns |
|  | Gr.1 | 112 | 173 | 0.45 | 0.25 | 0.82 | ** |
|  | Gr.1 | 173 | 254 | 0.83 | 0.44 | 1.57 | ns |
|  | Gr.2 | 28 | 56 | 14.84 | 8.18 | 26.93 | *** |
|  | Gr.2 | 56 | 112 | 1.27 | 0.70 | 2.30 | ns |
|  | Gr.2 | 112 | 173 | 0.39 | 0.22 | 0.71 | ** |
|  | Gr.2 | 173 | 254 | 0.48 | 0.25 | 0.91 | * |
|  | Gr.3 | 28 | 56 | 9.18 | 5.06 | 16.66 | *** |
|  | Gr.3 | 56 | 112 | 0.19 | 0.11 | 0.35 | *** |
|  | Gr.3 | 112 | 168 | 0.94 | 0.52 | 1.70 | ns |
|  | Gr.3 | 168 | 254 | 0.95 | 0.49 | 1.85 | ns |
| **DENV-2** | Gr.1 | 28 | 56 | 10.04 | 6.19 | 16.29 | *** |
|  | Gr.1 | 56 | 112 | 0.18 | 0.11 | 0.29 | *** |
|  | Gr.1 | 112 | 173 | 1.02 | 0.63 | 1.66 | ns |
|  | Gr.1 | 173 | 254 | 0.95 | 0.53 | 1.69 | ns |
|  | Gr.2 | 28 | 56 | 24.29 | 14.98 | 39.38 | *** |
|  | Gr.2 | 56 | 112 | 0.22 | 0.14 | 0.36 | *** |
|  | Gr.2 | 112 | 173 | 0.70 | 0.43 | 1.14 | ns |
|  | Gr.2 | 173 | 254 | 0.73 | 0.41 | 1.31 | ns |
|  | Gr.3 | 28 | 56 | 5.57 | 3.43 | 9.03 | *** |
|  | Gr.3 | 56 | 112 | 0.12 | 0.07 | 0.19 | *** |
|  | Gr.3 | 112 | 168 | 2.67 | 1.65 | 4.33 | *** |
|  | Gr.3 | 168 | 254 | 0.96 | 0.52 | 1.75 | ns |
| **DENV-3** | Gr.1 | 28 | 56 | 4.35 | 2.69 | 7.04 | *** |
|  | Gr.1 | 56 | 112 | 0.77 | 0.48 | 1.25 | ns |
|  | Gr.1 | 112 | 173 | 0.67 | 0.42 | 1.09 | ns |
|  | Gr.1 | 173 | 254 | 0.96 | 0.60 | 1.54 | ns |
|  | Gr.2 | 28 | 56 | 7.03 | 4.25 | 11.62 | *** |
|  | Gr.2 | 56 | 112 | 1.21 | 0.75 | 1.95 | ns |
|  | Gr.2 | 112 | 173 | 0.30 | 0.19 | 0.49 | *** |
|  | Gr.2 | 173 | 254 | 1.18 | 0.74 | 1.90 | ns |
|  | Gr.3 | 28 | 56 | 3.24 | 2.00 | 5.25 | *** |
|  | Gr.3 | 56 | 112 | 0.16 | 0.10 | 0.25 | *** |
|  | Gr.3 | 112 | 168 | 1.98 | 1.22 | 3.21 | ** |
|  | Gr.3 | 168 | 254 | 0.51 | 0.31 | 0.83 | ** |
| **DENV-4** | Gr.1 | 28 | 56 | 4.65 | 2.54 | 8.51 | *** |
|  | Gr.1 | 56 | 112 | 0.19 | 0.10 | 0.35 | *** |
|  | Gr.1 | 112 | 173 | 3.31 | 1.81 | 6.06 | *** |
|  | Gr.1 | 173 | 254 | 0.76 | 0.41 | 1.39 | ns |
|  | Gr.2 | 28 | 56 | 37.56 | 20.52 | 68.74 | *** |
|  | Gr.2 | 56 | 112 | 0.04 | 0.02 | 0.07 | *** |
|  | Gr.2 | 112 | 173 | 2.13 | 1.16 | 3.90 | * |
|  | Gr.2 | 173 | 254 | 1.67 | 0.91 | 3.05 | ns |
|  | Gr.3 | 28 | 56 | 3.62 | 1.98 | 6.62 | *** |
|  | Gr.3 | 56 | 112 | 0.15 | 0.08 | 0.28 | *** |
|  | Gr.3 | 112 | 168 | 3.53 | 1.93 | 6.45 | *** |
|  | Gr.3 | 168 | 254 | 0.29 | 0.16 | 0.55 | *** |

^a^Geometric mean ratio (GMR) compare PRNT50 geometric mean titers between time-points 1 and 2;

^b^Shown are the lower and upper limits of 95% confidence intervals;

^c^*P*-values were determined using a repeated ANOVA model: *, *p*<0.05; **, *p*<0.01; ***, *p*<0.001; ns, not significant.
