# Supplementary material for: Detection of post-vaccination enhanced dengue virus infection in macaques: An improved model for early assessment of dengue vaccines
Source: PLoS Pathog. 2019 Apr 22;15(4):e1007721. doi: 10.1371/journal.ppat.1007721 (PMC6497418; doi:10.1371/journal.ppat.1007721)
Supplement: S2 Table — (DOCX) [file ppat.1007721.s009.docx]

**S2 Table. Between-DENV type PRNT50 comparisons.**

| **Group** | **Time-point** | **DENV**  **type a** | **DENV**  **type b** | **GMR^a^** | **Lower limit^b^** | **Upper limit^b^** | **Significance^c^** |
| --- | --- | --- | --- | --- | --- | --- | --- |
| **Gr.1** | 28 | DENV-1 | DENV-2 | 1.84 | 0.92 | 3.66 | ns |
|  | 28 | DENV-1 | DENV-3 | 0.51 | 0.26 | 1.00 | * |
|  | 28 | DENV-1 | DENV-4 | 0.52 | 0.28 | 0.97 | * |
|  | 28 | DENV-2 | DENV-3 | 0.28 | 0.15 | 0.49 | *** |
|  | 28 | DENV-2 | DENV-4 | 0.28 | 0.15 | 0.53 | *** |
|  | 28 | DENV-3 | DENV-4 | 1.02 | 0.56 | 1.86 | ns |
|  | 56 | DENV-1 | DENV-2 | 2.41 | 1.26 | 4.61 | * |
|  | 56 | DENV-1 | DENV-3 | 0.29 | 0.14 | 0.60 | ** |
|  | 56 | DENV-1 | DENV-4 | 0.31 | 0.13 | 0.73 | ** |
|  | 56 | DENV-2 | DENV-3 | 0.12 | 0.06 | 0.23 | *** |
|  | 56 | DENV-2 | DENV-4 | 0.13 | 0.06 | 0.27 | *** |
|  | 56 | DENV-3 | DENV-4 | 1.09 | 0.52 | 2.29 | ns |
|  | 112 | DENV-1 | DENV-2 | 0.83 | 0.39 | 1.73 | ns |
|  | 112 | DENV-1 | DENV-3 | 0.52 | 0.21 | 1.28 | ns |
|  | 112 | DENV-1 | DENV-4 | 0.16 | 0.06 | 0.44 | ** |
|  | 112 | DENV-2 | DENV-3 | 0.62 | 0.29 | 1.36 | ns |
|  | 112 | DENV-2 | DENV-4 | 0.20 | 0.10 | 0.38 | *** |
|  | 112 | DENV-3 | DENV-4 | 0.32 | 0.16 | 0.62 | ** |
|  | 173 | DENV-1 | DENV-2 | 1.49 | 0.78 | 2.86 | ns |
|  | 173 | DENV-1 | DENV-3 | 0.62 | 0.30 | 1.29 | ns |
|  | 173 | DENV-1 | DENV-4 | 0.98 | 0.47 | 2.04 | ns |
|  | 173 | DENV-2 | DENV-3 | 0.42 | 0.26 | 0.68 | ** |
|  | 173 | DENV-2 | DENV-4 | 0.66 | 0.37 | 1.18 | ns |
|  | 173 | DENV-3 | DENV-4 | 1.58 | 0.87 | 2.88 | ns |
|  | 224 | DENV-1 | DENV-2 | 1.65 | 0.84 | 3.22 | ns |
|  | 224 | DENV-1 | DENV-3 | 0.49 | 0.28 | 0.84 | * |
|  | 224 | DENV-1 | DENV-4 | 1.81 | 0.90 | 3.65 | ns |
|  | 224 | DENV-2 | DENV-3 | 0.29 | 0.18 | 0.49 | *** |
|  | 224 | DENV-2 | DENV-4 | 1.10 | 0.58 | 2.06 | ns |
|  | 224 | DENV-3 | DENV-4 | 3.73 | 1.98 | 7.01 | *** |
|  | 254 | DENV-1 | DENV-2 | 1.58 | 0.96 | 2.62 | ns |
|  | 254 | DENV-1 | DENV-3 | 0.65 | 0.40 | 1.05 | ns |
|  | 254 | DENV-1 | DENV-4 | 0.80 | 0.41 | 1.54 | ns |
|  | 254 | DENV-2 | DENV-3 | 0.41 | 0.28 | 0.60 | *** |
|  | 254 | DENV-2 | DENV-4 | 0.50 | 0.26 | 0.98 | * |
|  | 254 | DENV-3 | DENV-4 | 1.22 | 0.64 | 2.34 | ns |
| **Gr.2** | 28 | DENV-1 | DENV-2 | 1.65 | 0.84 | 3.24 | ns |
|  | 28 | DENV-1 | DENV-3 | 0.37 | 0.19 | 0.70 | ** |
|  | 28 | DENV-1 | DENV-4 | 0.93 | 0.54 | 1.59 | ns |
|  | 28 | DENV-2 | DENV-3 | 0.22 | 0.12 | 0.42 | *** |
|  | 28 | DENV-2 | DENV-4 | 0.56 | 0.30 | 1.06 | ns |
|  | 28 | DENV-3 | DENV-4 | 2.52 | 1.38 | 4.60 | ** |
|  | 56 | DENV-1 | DENV-2 | 2.21 | 1.18 | 4.13 | * |
|  | 56 | DENV-1 | DENV-3 | 0.16 | 0.08 | 0.31 | *** |
|  | 56 | DENV-1 | DENV-4 | 1.28 | 0.56 | 2.92 | ns |
|  | 56 | DENV-2 | DENV-3 | 0.07 | 0.04 | 0.14 | *** |
|  | 56 | DENV-2 | DENV-4 | 0.58 | 0.26 | 1.27 | ns |
|  | 56 | DENV-3 | DENV-4 | 8.18 | 3.76 | 17.78 | *** |
|  | 112 | DENV-1 | DENV-2 | 0.36 | 0.18 | 0.72 | ** |
|  | 112 | DENV-1 | DENV-3 | 0.16 | 0.07 | 0.38 | *** |
|  | 112 | DENV-1 | DENV-4 | 0.09 | 0.04 | 0.23 | *** |
|  | 112 | DENV-2 | DENV-3 | 0.44 | 0.20 | 0.98 | * |
|  | 112 | DENV-2 | DENV-4 | 0.25 | 0.13 | 0.48 | *** |
|  | 112 | DENV-3 | DENV-4 | 0.57 | 0.30 | 1.10 | ns |
|  | 173 | DENV-1 | DENV-2 | 0.72 | 0.39 | 1.31 | ns |
|  | 173 | DENV-1 | DENV-3 | 0.12 | 0.06 | 0.24 | *** |
|  | 173 | DENV-1 | DENV-4 | 0.50 | 0.26 | 0.96 | * |
|  | 173 | DENV-2 | DENV-3 | 0.17 | 0.10 | 0.29 | *** |
|  | 173 | DENV-2 | DENV-4 | 0.70 | 0.40 | 1.24 | ns |
|  | 173 | DENV-3 | DENV-4 | 4.03 | 2.24 | 7.27 | *** |
|  | 224 | DENV-1 | DENV-2 | 0.44 | 0.23 | 0.86 | * |
|  | 224 | DENV-1 | DENV-3 | 0.28 | 0.17 | 0.46 | *** |
|  | 224 | DENV-1 | DENV-4 | 0.33 | 0.17 | 0.62 | ** |
|  | 224 | DENV-2 | DENV-3 | 0.64 | 0.36 | 1.11 | ns |
|  | 224 | DENV-2 | DENV-4 | 0.74 | 0.39 | 1.41 | ns |
|  | 224 | DENV-3 | DENV-4 | 1.16 | 0.62 | 2.17 | ns |
|  | 254 | DENV-1 | DENV-2 | 1.09 | 0.65 | 1.80 | ns |
|  | 254 | DENV-1 | DENV-3 | 0.30 | 0.20 | 0.47 | *** |
|  | 254 | DENV-1 | DENV-4 | 1.70 | 0.91 | 3.16 | ns |
|  | 254 | DENV-2 | DENV-3 | 0.28 | 0.18 | 0.42 | *** |
|  | 254 | DENV-2 | DENV-4 | 1.56 | 0.79 | 3.08 | ns |
|  | 254 | DENV-3 | DENV-4 | 5.60 | 2.94 | 10.65 | *** |
| **Gr.3** | 28 | DENV-1 | DENV-2 | 1.63 | 0.86 | 3.08 | ns |
|  | 28 | DENV-1 | DENV-3 | 0.89 | 0.48 | 1.66 | ns |
|  | 28 | DENV-1 | DENV-4 | 0.89 | 0.51 | 1.56 | ns |
|  | 28 | DENV-2 | DENV-3 | 0.55 | 0.31 | 0.96 | * |
|  | 28 | DENV-2 | DENV-4 | 0.55 | 0.31 | 0.98 | * |
|  | 28 | DENV-3 | DENV-4 | 1.00 | 0.55 | 1.81 | ns |
|  | 56 | DENV-1 | DENV-2 | 0.99 | 0.56 | 1.75 | ns |
|  | 56 | DENV-1 | DENV-3 | 0.32 | 0.16 | 0.62 | ** |
|  | 56 | DENV-1 | DENV-4 | 0.35 | 0.16 | 0.78 | * |
|  | 56 | DENV-2 | DENV-3 | 0.32 | 0.17 | 0.61 | ** |
|  | 56 | DENV-2 | DENV-4 | 0.35 | 0.18 | 0.70 | ** |
|  | 56 | DENV-3 | DENV-4 | 1.11 | 0.53 | 2.32 | ns |
|  | 112 | DENV-1 | DENV-2 | 0.57 | 0.29 | 1.11 | ns |
|  | 112 | DENV-1 | DENV-3 | 0.27 | 0.12 | 0.65 | ** |
|  | 112 | DENV-1 | DENV-4 | 0.35 | 0.14 | 0.89 | * |
|  | 112 | DENV-2 | DENV-3 | 0.48 | 0.22 | 1.03 | ns |
|  | 112 | DENV-2 | DENV-4 | 0.61 | 0.33 | 1.13 | ns |
|  | 112 | DENV-3 | DENV-4 | 1.27 | 0.66 | 2.48 | ns |
|  | 168 | DENV-1 | DENV-2 | 1.62 | 0.92 | 2.87 | ns |
|  | 168 | DENV-1 | DENV-3 | 0.58 | 0.30 | 1.13 | ns |
|  | 168 | DENV-1 | DENV-4 | 1.34 | 0.69 | 2.60 | ns |
|  | 168 | DENV-2 | DENV-3 | 0.36 | 0.23 | 0.57 | *** |
|  | 168 | DENV-2 | DENV-4 | 0.83 | 0.49 | 1.40 | ns |
|  | 168 | DENV-3 | DENV-4 | 2.29 | 1.26 | 4.18 | ns |
|  | 224 | DENV-1 | DENV-2 | 1.14 | 0.59 | 2.21 | ns |
|  | 224 | DENV-1 | DENV-3 | 0.69 | 0.41 | 1.16 | ns |
|  | 224 | DENV-1 | DENV-4 | 0.46 | 0.23 | 0.91 | * |
|  | 224 | DENV-2 | DENV-3 | 0.61 | 0.36 | 1.03 | ns |
|  | 224 | DENV-2 | DENV-4 | 0.40 | 0.21 | 0.76 | ** |
|  | 224 | DENV-3 | DENV-4 | 0.66 | 0.34 | 1.30 | ns |
|  | 254 | DENV-1 | DENV-2 | 1.51 | 0.92 | 2.48 | ns |
|  | 254 | DENV-1 | DENV-3 | 0.28 | 0.18 | 0.45 | *** |
|  | 254 | DENV-1 | DENV-4 | 0.40 | 0.21 | 0.78 | ** |
|  | 254 | DENV-2 | DENV-3 | 0.19 | 0.13 | 0.27 | *** |
|  | 254 | DENV-2 | DENV-4 | 0.27 | 0.13 | 0.53 | *** |
|  | 254 | DENV-3 | DENV-4 | 1.42 | 0.72 | 2.82 | ns |

^a^Geometric mean ratio (GMR) compare PRNT50 geometric mean titers between DENV types a and b;

^b^Shown are the lower and upper limits of 95% confidence intervals;

^c^*P*-values were determined using an ANCOVA model: *, *p*<0.05; **, *p*<0.01; ***, *p*<0.001; ns, not significant.
