# Supplementary material for: Detection of post-vaccination enhanced dengue virus infection in macaques: An improved model for early assessment of dengue vaccines
Source: PLoS Pathog. 2019 Apr 22;15(4):e1007721. doi: 10.1371/journal.ppat.1007721 (PMC6497418; doi:10.1371/journal.ppat.1007721)
Supplement: S3 Table — (DOCX) [file ppat.1007721.s010.docx]

**S3 Table. Between-group PRNT50 comparisons.**

| **DENV type** | **Time-point** | **Group a** | **Group b** | **GMR^a^** | **Lower limit^b^** | **Upper limit^b^** | **Significance^c^** |
| --- | --- | --- | --- | --- | --- | --- | --- |
| **DENV-1** | 28 | Gr.1 | Gr.2 | 0.48 | 0.17 | 1.41 | ns |
|  |  | Gr.1 | Gr.3 | 1.84 | 0.63 | 5.34 | ns |
|  |  | Gr.2 | Gr.3 | 3.80 | 1.31 | 11.06 | * |
|  | 56 | Gr.1 | Gr.2 | 0.86 | 0.30 | 2.50 | ns |
|  |  | Gr.1 | Gr.3 | 2.02 | 0.70 | 5.88 | ns |
|  |  | Gr.2 | Gr.3 | 2.35 | 0.81 | 6.84 | ns |
|  | 112 | Gr.1 | Gr.2 | 1.72 | 0.59 | 5.00 | ns |
|  |  | Gr.1 | Gr.3 | 0.62 | 0.21 | 1.80 | ns |
|  |  | Gr.2 | Gr.3 | 0.36 | 0.12 | 1.05 | ns |
|  | 168/173^d^ | Gr.1 | Gr.2 | 1.50 | 0.51 | 4.35 | ns |
|  |  | Gr.1 | Gr.3 | 1.29 | 0.44 | 3.75 | ns |
|  |  | Gr.2 | Gr.3 | 0.86 | 0.30 | 2.51 | ns |
|  | 224 | Gr.1 | Gr.2 | 2.33 | 0.80 | 6.77 | ns |
|  |  | Gr.1 | Gr.3 | 1.54 | 0.52 | 4.55 | ns |
|  |  | Gr.2 | Gr.3 | 0.66 | 0.22 | 1.95 | ns |
|  | 254 | Gr.1 | Gr.2 | 0.86 | 0.30 | 2.50 | ns |
|  |  | Gr.1 | Gr.3 | 1.47 | 0.50 | 4.36 | ns |
|  |  | Gr.2 | Gr.3 | 1.71 | 0.58 | 5.07 | ns |
| **DENV-2** | 28 | Gr.1 | Gr.2 | 0.37 | 0.17 | 0.83 | * |
|  |  | Gr.1 | Gr.3 | 1.57 | 0.70 | 3.52 | ns |
|  |  | Gr.2 | Gr.3 | 4.26 | 1.91 | 9.52 | ** |
|  | 56 | Gr.1 | Gr.2 | 0.89 | 0.40 | 2.00 | ns |
|  |  | Gr.1 | Gr.3 | 0.87 | 0.39 | 1.95 | ns |
|  |  | Gr.2 | Gr.3 | 0.98 | 0.44 | 2.18 | ns |
|  | 112 | Gr.1 | Gr.2 | 1.11 | 0.50 | 2.49 | ns |
|  |  | Gr.1 | Gr.3 | 0.57 | 0.26 | 1.28 | ns |
|  |  | Gr.2 | Gr.3 | 0.51 | 0.23 | 1.15 | ns |
|  | 168/173^d^ | Gr.1 | Gr.2 | 0.77 | 0.34 | 1.71 | ns |
|  |  | Gr.1 | Gr.3 | 1.50 | 0.67 | 3.35 | ns |
|  |  | Gr.2 | Gr.3 | 1.96 | 0.88 | 4.37 | ns |
|  | 224 | Gr.1 | Gr.2 | 0.92 | 0.41 | 2.06 | ns |
|  |  | Gr.1 | Gr.3 | 1.41 | 0.62 | 3.20 | ns |
|  |  | Gr.2 | Gr.3 | 1.53 | 0.68 | 3.47 | ns |
|  | 254 | Gr.1 | Gr.2 | 0.59 | 0.27 | 1.33 | ns |
|  |  | Gr.1 | Gr.3 | 1.52 | 0.67 | 3.46 | ns |
|  |  | Gr.2 | Gr.3 | 2.56 | 1.12 | 5.82 | * |
| **DENV-3** | 28 | Gr.1 | Gr.2 | 0.28 | 0.12 | 0.64 | ** |
|  |  | Gr.1 | Gr.3 | 2.89 | 1.27 | 6.56 | * |
|  |  | Gr.2 | Gr.3 | 10.34 | 4.50 | 23.76 | *** |
|  | 56 | Gr.1 | Gr.2 | 0.45 | 0.20 | 1.03 | ns |
|  |  | Gr.1 | Gr.3 | 2.16 | 0.95 | 4.89 | ns |
|  |  | Gr.2 | Gr.3 | 4.77 | 2.10 | 10.83 | *** |
|  | 112 | Gr.1 | Gr.2 | 0.70 | 0.31 | 1.60 | ns |
|  |  | Gr.1 | Gr.3 | 0.44 | 0.19 | 0.99 | * |
|  |  | Gr.2 | Gr.3 | 0.62 | 0.27 | 1.41 | ns |
|  | 168/173^d^ | Gr.1 | Gr.2 | 0.32 | 0.14 | 0.72 | ** |
|  |  | Gr.1 | Gr.3 | 1.29 | 0.57 | 2.92 | ns |
|  |  | Gr.2 | Gr.3 | 4.04 | 1.78 | 9.17 | ** |
|  | 224 | Gr.1 | Gr.2 | 1.70 | 0.75 | 3.87 | ns |
|  |  | Gr.1 | Gr.3 | 2.91 | 1.27 | 6.68 | * |
|  |  | Gr.2 | Gr.3 | 1.71 | 0.74 | 3.92 | ns |
|  | 254 | Gr.1 | Gr.2 | 0.39 | 0.17 | 0.89 | * |
|  |  | Gr.1 | Gr.3 | 0.68 | 0.30 | 1.57 | ns |
|  |  | Gr.2 | Gr.3 | 1.73 | 0.75 | 3.99 | ns |
| **DENV-4** | 28 | Gr.1 | Gr.2 | 0.73 | 0.32 | 1.68 | ns |
|  |  | Gr.1 | Gr.3 | 2.80 | 1.23 | 6.40 | * |
|  |  | Gr.2 | Gr.3 | 3.82 | 1.67 | 8.73 | ** |
|  | 56 | Gr.1 | Gr.2 | 5.93 | 2.60 | 13.54 | *** |
|  |  | Gr.1 | Gr.3 | 2.18 | 0.96 | 4.98 | ns |
|  |  | Gr.2 | Gr.3 | 0.37 | 0.16 | 0.84 | * |
|  | 112 | Gr.1 | Gr.2 | 1.26 | 0.55 | 2.89 | ns |
|  |  | Gr.1 | Gr.3 | 1.76 | 0.77 | 4.01 | ns |
|  |  | Gr.2 | Gr.3 | 1.39 | 0.61 | 3.17 | ns |
|  | 168/173^d^ | Gr.1 | Gr.2 | 0.81 | 0.36 | 1.86 | ns |
|  |  | Gr.1 | Gr.3 | 1.87 | 0.82 | 4.27 | ns |
|  |  | Gr.2 | Gr.3 | 2.30 | 1.01 | 5.25 | * |
|  | 224 | Gr.1 | Gr.2 | 0.54 | 0.24 | 1.23 | ns |
|  |  | Gr.1 | Gr.3 | 0.50 | 0.22 | 1.16 | ns |
|  |  | Gr.2 | Gr.3 | 0.93 | 0.40 | 2.15 | ns |
|  | 254 | Gr.1 | Gr.2 | 1.79 | 0.78 | 4.08 | ns |
|  |  | Gr.1 | Gr.3 | 0.72 | 0.31 | 1.67 | ns |
|  |  | Gr.2 | Gr.3 | 0.40 | 0.17 | 0.94 | * |

^a^Geometric mean ratio (GMR) compare PRNT50 geometric mean titers between groups a

and b;

^b^Shown are the lower and upper limits of 95% confidence intervals;

^c^*P*-values were determined using a repeated ANOVA model followed by a global F-test: *, *p*<0.05; **, *p*<0.01; ***, *p*<0.001; ns, not significant.

^d^Sera were collected at day 173 in Gr.1-2 and at day 168 in Gr.3.
