# Supplementary material for: Detection of post-vaccination enhanced dengue virus infection in macaques: An improved model for early assessment of dengue vaccines
Source: PLoS Pathog. 2019 Apr 22;15(4):e1007721. doi: 10.1371/journal.ppat.1007721 (PMC6497418; doi:10.1371/journal.ppat.1007721)
Supplement: S4 Table — (DOCX) [file ppat.1007721.s011.docx]

**S4 Table. RNAemia area under the curves, peaks and durations after challenge of Gr.1-2 and Gr.4 with either DENV-1 0111/2011 or DENV-2 0126/2010 (frozen-thawed sera).**

| **DENV strain** | **Parameter^a^** | **Group** | **Geometric mean/**  **mean with 95% CI^b^** | **Between-group comparison^c^** | | ***P*-value^d^** |
| --- | --- | --- | --- | --- | --- | --- |
|  |  |  |  | **Compared groups** | **GMR/Difference with 95% CI** |  |
| **DENV-1 0111/2011** | AUC | Gr.1 | 1192.4 (94.6; 15031) | Gr.1/Gr.4 | 0.09 (0.01; 1.26) | 0.068 |
|  |  | Gr.2 | 1733.4 (264.5; 11360) |  |  |  |
|  |  | Gr.4 | 12643 (2357.9; 67791) | Gr.2/Gr.4 | 0.14 (0.02; 1.12) | 0.060 |
|  | Peak | Gr.1 | 75162 (783.4; 7.21x10^6^) | Gr.1/Gr.4 | 0.38 (0.00; 33.42) | 0.585 |
|  |  | Gr.2 | 570164 (37586; 8.65x10^6^) |  |  |  |
|  |  | Gr.4 | 199526 (34856; 1.14x10^6^) | Gr.2/Gr.4 | 2.86 (0.18; 45.46) | 0.455 |
|  | Duration | Gr.1 | 6 (4.8; 7.2) | Gr.1-Gr.4 | -3.6 (-4.99; -2.21) | 0.000 |
|  |  | Gr.2 | 5.8 (3.8; 7.8) |  |  |  |
|  |  | Gr.4 | 9.6 (8.5; 10.7) | Gr.2-Gr.4 | -3.80 (-5.83; -1.77) | 0.004 |
| **DENV-2 0126/2010** | AUC | Gr.1 | 630.6 (149.2; 2665.4) | Gr.1/Gr.4 | 0.38 (0.09; 1.60) | 0.152 |
|  |  | Gr.2 | 2184.9 (1067.5; 4472.0) |  |  |  |
|  |  | Gr.4 | 1675.7 (724.2; 3877.0) | Gr.2/Gr.4 | 1.30 (0.52; 3.27) | 0.523 |
|  | Peak | Gr.1 | 52240 (2860.6; 953980) | Gr.1/Gr.4 | 0.81 (0.05; 14.36) | 0.723 |
|  |  | Gr.2 | 207014 (100902; 424717) |  |  |  |
|  |  | Gr.4 | 64863 (14580; 288570) | Gr.2/Gr.4 | 3.19 (0.73; 13.95) | 0.103 |
|  | Duration | Gr.1 | 4.4 (2.0; 6.8) | Gr.1-Gr.4 | -2.40 (-4.78; -0.02) | 0.049 |
|  |  | Gr.2 | 6.0 (5.1; 6.9) |  |  |  |
|  |  | Gr.4 | 6.8 (5.8; 7.8) | Gr.2-Gr.4 | -0.80 (-1.94; 0.34) | 0.142 |

^a^RNAemia were measured daily, before and until, at least, day 12 post-DENV challenge, by DENV-specific real-time RT-PCR, and expressed as genome equivalents (ge)/mL. No RNAemia was detected in any group after day 10 post-challenge. RNAemia area under the curves (AUC) for days 1-10 post-challenge were computed, for each monkey, on the log_10_-transformed values by applying the trapezoidal rule, further normalized over the number of days (*i.e.* divided by 10) and back-transformed to the original unit, *i.e.* ge/mL. Peaks, also expressed as ge/mL, correspond to the highest RNAemia titers detected after DENV challenge. Durations correspond to the number of days with detectable RNAemia;

^b^Shown are the geometric means and 95% confidence intervals (CI) for RNAemia AUC and peaks, and means and 95% CI for RNAemia durations, all from macaques from the same group challenged with the same DENV strain;

^c^Shown are the geometric mean ratio (GMR) and 95% CI for RNAemia AUC and peaks, and differences for RNAemia durations, between the indicated vaccinated and non-vaccinated groups;

^d^*P*-values were determined using, for RNAemia AUC and durations, an ANOVA model, and, for RNAemia peaks, a non-parametric analysis (ANOVA on ranks). No adjustment for multiplicity was performed as these analyses were performed to assess not only efficacy but also safety.
