# Supplementary material for: Detection of post-vaccination enhanced dengue virus infection in macaques: An improved model for early assessment of dengue vaccines
Source: PLoS Pathog. 2019 Apr 22;15(4):e1007721. doi: 10.1371/journal.ppat.1007721 (PMC6497418; doi:10.1371/journal.ppat.1007721)
Supplement: S5 Table — (DOCX) [file ppat.1007721.s012.docx]

**S5 Table. Viremia area under the curves, peaks and durations after challenge of Gr.3 and Gr.5 with either DENV-2 0126/2010 or DENV-2 S16803 (fresh sera).**

| **DENV strain** | **Parameter^a^** | **Group** | **Geometric mean/mean with 95% CI^b^** | **Between-group comparison^c^** | ***P*-value^d^** |
| --- | --- | --- | --- | --- | --- |
| **DENV-2 0126/2010** | AUC | Gr.3 | 7.7 (1.5; 40.2) | 0.17 (0.03; 0.85) | 0.036 |
|  |  | Gr.5 | 46.4 (21.7; 99.1) |  |  |
|  | Peak | Gr.3 | 50.6 (0.9; 2842.7) | 0.04 (0.00; 1.96) | 0.032 |
|  |  | Gr.5 | 1351.2 (291.3; 6267.6) |  |  |
|  | Duration | Gr.3 | 2.2 (-0.6; 5.0) | -3.40 (-6.24; -0.56) | 0.026 |
|  |  | Gr.5 | 5.6 (3.9; 7.3) |  |  |
| **DENV-2 S16803** | AUC | Gr.3 | 19.5 (3.1; 123.9) | 1.64 (0.29; 9.41) | 0.507 |
|  |  | Gr.5 | 11.9 (4.1; 34.6) |  |  |
|  | Peak | Gr.3 | 645.7 (4.4; 93983) | 4.06 (0.04; 465.89) | 0.500 |
|  |  | Gr.5 | 159.2 (36.7; 690.1) |  |  |
|  | Duration | Gr.3 | 3.8 (1.7; 5.8) | 0.15 (-2.29; 2.59) | 0.888 |
|  |  | Gr.5 | 3.6 (1.3; 5.9) |  |  |

^a^Viremia were measured daily, before and until, at least, day 12 post-DENV challenge, by plaque assay, and expressed as plaque-forming units (PFU)/mL. No viremia was detected in any group after day 10 post-challenge. Viremia area under the curves (AUC) for days 1-10 post-challenge were computed, for each monkey, on the log_10_-transformed values by applying the trapezoidal rule, further normalized over the number of days (*i.e.* divided by 10) and back-transformed to the original unit, *i.e.* PFU/mL. Peaks, also expressed as PFU/mL, correspond to the highest viremia titers detected after DENV challenge. Durations correspond to the number of days with detectable viremia;

^b^Shown are the geometric means and 95% confidence intervals (CI) for viremia AUC and peaks, and means and 95% CI for viremia durations, all from macaques from the same group challenged with the same DENV strain;

^c^Shown are the geometric mean ratio (GMR) and 95% CI for viremia AUC and peaks, and differences for viremia durations, between Gr.3 and Gr.5;

^d^*P*-values were determined using, for viremia AUC and durations, an ANOVA model, and, for viremia peaks, a non-parametric analysis (ANOVA on ranks). No adjustment for multiplicity was performed as these analyses were performed to assess not only efficacy but also safety.
