# Supplementary material for: Detection of post-vaccination enhanced dengue virus infection in macaques: An improved model for early assessment of dengue vaccines
Source: PLoS Pathog. 2019 Apr 22;15(4):e1007721. doi: 10.1371/journal.ppat.1007721 (PMC6497418; doi:10.1371/journal.ppat.1007721)
Supplement: S6 Table — (DOCX) [file ppat.1007721.s013.docx]

**S6 Table. RNAemia area under the curves, peaks and durations after challenge of Gr.3**

**and Gr.5 with either DENV-2 0126/2010 or DENV-2 S16803 (frozen-thawed sera).**

| **DENV strain** | **Parameter^a^** | **Group** | **Geometric mean/mean**  **with 95% CI^b^** | **Between-group comparison^c^** | ***P*-value^d^** |
| --- | --- | --- | --- | --- | --- |
| **DENV-2 0126/2010** | AUC | Gr.3 | 404.3 (71.1; 2299.7) | 0.25 (0.04; 1.36) | 0.090 |
|  |  | Gr.5 | 1640.6 (794.2; 3388.9) |  |  |
|  | Peak | Gr.3 | 10222 (159.6; 654827) | 0.09 (0.00; 5.57) | 0.131 |
|  |  | Gr.5 | 109781 (20175; 597385) |  |  |
|  | Duration | Gr.3 | 3.6 (0.5; 6.7) | -2.60 (-5.69; 0.49) | 0.085 |
|  |  | Gr.5 | 6.2 (4.6; 7.8) |  |  |
| **DENV-2 S16803** | AUC | Gr.3 | 6762.0 (980.9; 46613) | 1.54 (0.23; 10.40) | 0.601 |
|  |  | Gr.5 | 4379.2 (1077.6; 17796) |  |  |
|  | Peak | Gr.3 | 1.26x10^6^ (7449.2; 2.12x10^8^) | 6.99 (0.05; 910.66) | 0.152 |
|  |  | Gr.5 | 179616 (36781; 877133) |  |  |
|  | Duration | Gr.3 | 6.5 (5.6; 7.4) | -0.70 (-2.08; 0.68) | 0.263 |
|  |  | Gr.5 | 7.2 (5.8; 8.6) |  |  |

^a^RNAemia were measured daily, before and until, at least, day 12 post-DENV challenge, by DENV-specific real-time RT-PCR, and expressed as genome equivalents (ge)/mL. No RNAemia was detected in any group after day 10 post-challenge. RNAemia area under the curves (AUC) for days 1-10 post-challenge were computed, for each monkey, on the log_10_-transformed values by applying the trapezoidal rule, further normalized over the number of days (*i.e.* divided by 10) and back-transformed to the original unit, *i.e.* ge/mL. Peaks, also expressed as ge/mL, correspond to the highest RNAemia titers detected after DENV challenge. Durations correspond to the number of days with detectable RNAemia;

^b^Shown are the geometric means and 95% confidence intervals (CI) for RNAemia AUC and peaks, and means and 95% CI for RNAemia durations, all from macaques from the same group challenged with the same DENV strain;

^c^Shown are the geometric mean ratio (GMR) and 95% CI for RNAemia AUC and peaks, and differences for RNAemia durations, between Gr.3 and Gr.5;

^d^*P*-values were determined using, for RNAemia AUC and durations, an ANOVA model, and, for RNAemia peaks, a non-parametric analysis (ANOVA on ranks). No adjustment for multiplicity was performed as these analyses were performed to assess not only efficacy but also safety.
