# Supplementary material for: Detection of post-vaccination enhanced dengue virus infection in macaques: An improved model for early assessment of dengue vaccines
Source: PLoS Pathog. 2019 Apr 22;15(4):e1007721. doi: 10.1371/journal.ppat.1007721 (PMC6497418; doi:10.1371/journal.ppat.1007721)
Supplement: S1 Data — (DOCX) [file ppat.1007721.s014.docx]

**Group 1**

| **AF104** | **NHPs Transfer** | | **-1d pre-challenge*** | | **7d post-challenge** | |
| --- | --- | --- | --- | --- | --- | --- |
| **Erythrocytes** | 5.88 millions/μl | | 4.71 millions/mm^3^ | | 4.69 millions/mm^3^ | |
| Hemoglobin | 13.5 g/dL | | 10.40 g/dL | | 10.40 g/dL | |
| Globular Volume | 40.1 % | |  | |  | |
| Hematocrit (HCT) |  | | 33.60 % | | 33.50 % | |
| Mean Corpuscular Volume (MCV) | 68.2 fl | | 71.30 fm^3^ | | 71.40 fm^3^ | |
| Mean Corpuscular Hemoglobin (MCH) |  | | 22.10 pg | | 22.20 pg | |
| Mean Corpuscular Hemoglobin Concentration (MCHC) | 33.6 % | | 31 g/dL | | 31 g/dL | |
| Note:  *Normocytic and normochromic erythrocytes. | | | | | | |
| **Leukocytes** | 9.1 x 10^3^/μl | | 7.50 x 10^3^/mm^3^ | | 3.40 x 10^3^/mm^3^ | |
|  | Relative (%) | Absolute (μL) | Relative (%) | Absolute (10^3^/mm^3^) | Relative (%) | Absolute (10^3^/mm^3^) |
| Basophils | 2 | 182 | 0 | 0 | 0 | 0 |
| Eosinophils | 2 | 182 | 0 | 0 | 0 | 0 |
| Myelocytes | 0 | 0 |  |  |  |  |
| Metamyelocytes | 0 | 0 |  |  |  |  |
| Neutrophils (Bands) | 0 | 0 | 0 | 0 | 0 | 0 |
| Neutrophils (Segmented) | 46 | 4186 | 67 | 0.67 | 58 | 1.97 |
| Lymphocytes | 48 | 4368 | 31 | 0.31 | 38 | 1.29 |
| Monocytes | 2 | 182 | 2 | 0.02 | 4 | 0.14 |
| **Platelets** | 560 x 10^3^/μl | | 429 x 10^3^/mm^3^ | | 481 x 10^3^/mm^3^ | |
| **ALT** | 26 UI/L  (0 a 82 UI/L) | | 16 U/L  (18 a 129 U/L) | | 46 U/L  (18 a 129 U/L) | |
| **AST** | 42 UI/L  (13 a 37 UI/L) | | 45 U/L  (23 a 70 U/L) | | 65 U/L  (23 a 70 U/L) | |
| **Creatinine** | 1.0 mg/dL  (0.8 a 2.32 mg/dL) | | 0.7 mg/dL  (0.30 a 1.30 mg/dL) | | 0.7 mg/dL  (0.30 a 1.30 mg/dL) | |
| **GGT** | 52 U/L  (47.7 a 86 UI/L) | | 51 U/L  (48 a 130 U/L) | | 53 U/L  (48 a 130 U/L) | |

| **AF124** | **NHPs Transfer** | | **-1d pre-challenge*** | | **7d post-challenge** | |
| --- | --- | --- | --- | --- | --- | --- |
| **Erythrocytes** | 5.87 millions/μl | | 4.80 millions/mm^3^ | | 4.61 millions/mm^3^ | |
| Hemoglobin | 13.8 g/dL | | 11 g/dL | | 10.60 g/dL | |
| Globular Volume | 43.0 % | |  | |  | |
| Hematocrit (HCT) |  | | 36.60 % | | 35.40 % | |
| Mean Corpuscular Volume (MCV) | 73.3 fl | | 76.30 fm^3^ | | 76.80 fm^3^ | |
| Mean Corpuscular Hemoglobin (MCH) |  | | 22.90 pg | | 23 pg | |
| Mean Corpuscular Hemoglobin Concentration (MCHC) | 32.0 % | | 30.10 g/dL | | 29.90 g/dL | |
| Note:  *Normocytic and normochromic erythrocytes. | | | | | | |
| **Leukocytes** | 6.8 x 10^3^/μl | | 6.70 x 10^3^/mm^3^ | | 3 x 10^3^/mm^3^ | |
|  | Relative (%) | Absolute (μL) | Relative (%) | Absolute (10^3^/mm^3^) | Relative (%) | Absolute (10^3^/mm^3^) |
| Basophils | 0 | 0 | 0 | 0 | 2 | 0.06 |
| Eosinophils | 2 | 136 | 0 | 0 | 0 | 0 |
| Myelocytes | 0 | 0 |  |  |  |  |
| Metamyelocytes | 0 | 0 |  |  |  |  |
| Neutrophils (Bands) | 0 | 0 | 0 | 0 | 0 | 0 |
| Neutrophils (Segmented) | 74 | 5032 | 73 | 0.73 | 57 | 1.71 |
| Lymphocytes | 18 | 1224 | 26 | 0.26 | 39 | 1.17 |
| Monocytes | 6 | 408 | 1 | 0.01 | 2 | 0.06 |
| **Platelets** | 400 x 10^3^/μl | | 526 x 10^3^/mm^3^ | | 443 x 10^3^/mm^3^ | |
| **ALT** | 25 UI/L  (0 a 82 UI/L) | | 13 U/L  (18 a 129 U/L) | | 54 U/L  (18 a 129 U/L) | |
| **AST** | 40 UI/L  (13 a 37 UI/L) | | 48 U/L  (23 a 70 U/L) | | 75 U/L  (23 a 70 U/L) | |
| **Creatinine** | 0.9 mg/dL  (0.8 a 2.32 mg/dL) | | 0.5 mg/dL  (0.30 a 1.30 mg/dL) | | 0.5 mg/dL  (0.30 a 1.30 mg/dL) | |
| **GGT** | 47 U/L  (47.7 a 86 UI/L) | | 60 U/L  (48 a 130 U/L) | | 54 U/L  (48 a 130 U/L) | |

| **AF125** | **NHPs Transfer** | | **-1d pre-challenge*** | | **7d post-challenge** | |
| --- | --- | --- | --- | --- | --- | --- |
| **Erythrocytes** | 6 millions/μl | | 5.72 millions/mm^3^ | | 4.75 millions/mm^3^ | |
| Hemoglobin | 13 g/dL | | 12.50 g/dL | | 10.40 g/dL | |
| Globular Volume | 40.3 % | |  | |  | |
| Hematocrit (HCT) |  | | 42.40 % | | 35.50 % | |
| Mean Corpuscular Volume (MCV) | 40.3 % | | 74.10 fm^3^ | | 74.70 fm^3^ | |
| Mean Corpuscular Hemoglobin (MCH) |  | | 21.90 pg | | 21.90 pg | |
| Mean Corpuscular Hemoglobin Concentration (MCHC) | 32.2 % | | 29.50 g/dL | | 29.30 g/dL | |
| Note:  *Normocytic and normochromic erythrocytes. | | | | | | |
| **Leukocytes** | 11.6 x 10^3^/mm^3^μl | | 10.50 x 10^3^/mm^3^ | | 4.50 x 10^3^/mm^3^ | |
|  | Relative (%) | Absolute (μL) | Relative (%) | Absolute (10^3^/mm^3^) | Relative (%) | Absolute (10^3^/mm^3^) |
| Basophils | 0 | 0 | 0 | 0 | 4 | 0.18 |
| Eosinophils | 2 | 232 | 0 | 0 | 1 | 0.04 |
| Myelocytes | 0 | 0 |  |  |  |  |
| Metamyelocytes | 0 | 0 |  |  |  |  |
| Neutrophils (Bands) | 0 | 0 | 0 | 0 | 1 | 0.04 |
| Neutrophils (Segmented) | 54 | 6264 | 76 | 0.76 | 66 | 2.97 |
| Lymphocytes | 42 | 4872 | 18 | 0.18 | 24 | 1.08 |
| Monocytes | 2 | 232 | 4 | 0.04 | 4 | 0.18 |
| **Platelets** | 540 x 10^3^/mm^3^μl | | 330 x 10^3^/mm^3^ | | 156 x 10^3^/mm^3^ | |
| **ALT** | 36 UI/L  (0 a 82 UI/L) | | 13 U/L  (18 a 129 U/L) | | 55 U/L  (18 a 129 U/L) | |
| **AST** | 34 UI/L  (13 a 37 UI/L) | | 41 U/L  (23 a 70 U/L) | | 96 U/L  (23 a 70 U/L) | |
| **Creatinine** | 0.9 mg/dL  (0.8 a 2.32 mg/dL) | | 0.8 mg/dL  (0.30 a 1.30 mg/dL) | | 0.9 mg/dL  (0.30 a 1.30 mg/dL) | |
| **GGT** | 77 U/L  (47.7 a 86 UI/L) | | 85 U/L  (48 a 130 U/L) | | 64 U/L  (48 a 130 U/L) | |

| **AF138** | **NHPs Transfer** | | **-1d pre-challenge*** | | **7d post-challenge** | |
| --- | --- | --- | --- | --- | --- | --- |
| **Erythrocytes** | 6.24 millions/μl | | 5.28 millions/mm^3^ | | 4.78 millions/mm^3^ | |
| Hemoglobin | 13.5 g/dL | | 11.20 g/dL | | 10.20 g/dL | |
| Globular Volume | 41.5 % | |  | |  | |
| Hematocrit (HCT) |  | | 36.80 % | | 34 % | |
| Mean Corpuscular Volume (MCV) | 66.5 fl | | 69.70 fm^3^ | | 71.10 fm^3^ | |
| Mean Corpuscular Hemoglobin (MCH) |  | | 21.20 pg | | 21.30 pg | |
| Mean Corpuscular Hemoglobin Concentration (MCHC) | 32.5 % | | 30.40 g/dL | | 30 g/dL | |
| Note:  *Normocytic and normochromic erythrocytes. | | | | | | |
| **Leukocytes** | 12.4 x 10^3^/μL | | 7.50 x 10^3^/mm^3^ | | 3.10 x 10^3^/mm^3^ | |
|  | Relative (%) | Absolute (μL) | Relative (%) | Absolute (10^3^/mm^3^) | Relative (%) | Absolute (10^3^/mm^3^) |
| Basophils | 0 | 0 | 0 | 0 | 0 | 0 |
| Eosinophils | 2 | 248 | 0 | 0 | 2 | 0.06 |
| Myelocytes | 0 | 0 |  |  |  |  |
| Metamyelocytes | 0 | 0 |  |  |  |  |
| Neutrophils (Bands) | 0 | 0 | 0 | 0 | 1 | 0.03 |
| Neutrophils (Segmented) | 63 | 7812 | 54 | 0.54 | 29 | 0.90 |
| Lymphocytes | 30 | 3720 | 45 | 0.45 | 64 | 1.98 |
| Monocytes | 5 | 620 | 1 | 0.01 | 4 | 0.12 |
| **Platelets** | 600 x 10^3^/μl | | 458 x 10^3^/mm^3^ | | 412 x 10^3^/mm^3^ | |
| **ALT** | 30 UI/L  (0 a 82 UI/L) | | 22 U/L  (18 a 129 U/L) | | 38 U/L  (18 a 129 U/L) | |
| **AST** | 44 UI/L  (13 a 37 UI/L) | | 47 U/L  (23 a 70 U/L) | | 74 U/L  (23 a 70 U/L) | |
| **Creatinine** | 0.9 mg/dL  (0.8 a 2.32 mg/dL) | | 0.7 mg/dL  (0.30 a 1.30 mg/dL) | | 0.7 mg/dL  (0.30 a 1.30 mg/dL) | |
| **GGT** | 58 U/L  (47.7 a 86 UI/L) | | 56 U/L  (48 a 130 U/L) | | 53 U/L  (48 a 130 U/L) | |

| **AG101** | **NHPs Transfer** | | **-1d pre-challenge*** | | **7d post-challenge** | |
| --- | --- | --- | --- | --- | --- | --- |
| **Erythrocytes** | 6.21 millions/μl | | 5.42 millions/mm^3^ | | 5.16 millions/mm^3^ | |
| Hemoglobin | 14.8 g/dL | | 13 g/dL | | 12.60 g/dL | |
| Globular Volume | 44.5 % | |  | |  | |
| Hematocrit (HCT) |  | | 41.20 % | | 39.60 % | |
| Mean Corpuscular Volume (MCV) | 71.7 fl | | 76 fm^3^ | | 76.70 fm^3^ | |
| Mean Corpuscular Hemoglobin (MCH) |  | | 24 pg | | 24.40 pg | |
| Mean Corpuscular Hemoglobin Concentration (MCHC) | 33.2 % | | 31.60 g/dL | | 31.80 g/dL | |
| Note:  *Normocytic and normochromic erythrocytes. | | | | | | |
| **Leukocytes** | 6.4 x 10^3^/μl | | 5.50 x 10^3^/mm^3^ | | 5.10 x 10^3^/mm^3^ | |
|  | Relative (%) | Absolute (μL) | Relative (%) | Absolute (10^3^/mm^3^) | Relative (%) | Absolute (10^3^/mm^3^) |
| Basophils | 0 | 0 | 0 | 0 | 0 | 0 |
| Eosinophils | 8 | 512 | 1 | 0.06 | 0 | 0 |
| Myelocytes | 0 | 0 |  |  |  |  |
| Metamyelocytes | 0 | 0 |  |  |  |  |
| Neutrophils (Bands) | 0 | 0 | 0 | 0 | 1 | 0.05 |
| Neutrophils (Segmented) | 53 | 3392 | 38 | 2.09 | 63 | 3.21 |
| Lymphocytes | 37 | 2368 | 60 | 3.30 | 36 | 1.84 |
| Monocytes | 2 | 128 | 1 | 0.01 | 0 | 0 |
| **Platelets** | 550 x 10^3^/μl | | 382 x 10^3^/mm^3^ | | 379 x 10^3^/mm^3^ | |
| **ALT** | 25 UI/L  (0 a 82 UI/L) | | 21 U/L  (18 a 129 U/L) | | 37 U/L  (18 a 129 U/L) | |
| **AST** | 39 UI/L  (13 a 37 UI/L) | | 59 U/L  (23 a 70 U/L) | | 75 U/L  (23 a 70 U/L) | |
| **Creatinine** | 0.7 mg/dL  (0.8 a 2.32 mg/dL) | | 0.7 mg/dL  (0.30 a 1.30 mg/dL) | | 0.7 mg/dL  (0.30 a 1.30 mg/dL) | |
| **GGT** | 67 U/L  (47.7 a 86 UI/L) | | 87 U/L  (48 a 130 U/L) | | 56 U/L  (48 a 130 U/L) | |

| **AG119** | **NHPs Transfer** | | **-1d pre-challenge*** | | **7d post-challenge** | |
| --- | --- | --- | --- | --- | --- | --- |
| **Erythrocytes** | 5.96 millions/μl | |  | | 5.16 millions/mm^3^ | |
| Hemoglobin | 13.8 g/dL | |  | | 11.60 g/dL | |
| Globular Volume | 40.6 % | |  | |  | |
| Hematocrit (HCT) |  | |  | | 38.40 % | |
| Mean Corpuscular Volume (MCV) | 68.1 fl | |  | | 74.40 fm^3^ | |
| Mean Corpuscular Hemoglobin (MCH) |  | |  | | 22.50 pg | |
| Mean Corpuscular Hemoglobin Concentration (MCHC) | 33.9 % | |  | | 30.20 g/dL | |
| Note:  **^#^**Normocytic and normochromic erythrocytes. | | | | | | |
| **Leukocytes** | 12.0 x 10^3^/μl | |  | | 4.80 x 10^3^/mm^3^ | |
|  | Relative (%) | Absolute (μL) | Relative (%) | Absolute (10^3^/mm^3^) | Relative (%) | Absolute (10^3^/mm^3^) |
| Basophils | 0 | 0 |  |  | 0 | 0 |
| Eosinophils | 4 | 480 |  |  | 0 | 0 |
| Myelocytes | 0 | 0 |  |  |  |  |
| Metamyelocytes | 0 | 0 |  |  |  |  |
| Neutrophils (Bands) | 0 | 0 |  |  | 1 | 0.05 |
| Neutrophils (Segmented) | 68 | 8160 |  |  | 75 | 3.60 |
| Lymphocytes | 26 | 3120 |  |  | 22 | 1.06 |
| Monocytes | 2 | 240 |  |  | 2 | 0.10 |
| **Platelets** | 402 x 10^3^/μl | |  | | 327 x 10^3^/mm^3^ | |
| Note:  *-1d pre-challenge sample coagulated. | | | | | | |
| **ALT** | 55 UI/L  (0 a 82 UI/L) | | 34 U/L  (18 a 129 U/L) | | 109 U/L  (18 a 129 U/L) | |
| **AST** | 49 UI/L  (13 a 37 UI/L) | | 41 U/L  (23 a 70 U/L) | | 134 U/L  (23 a 70 U/L) | |
| **Creatinine** | 0.8 mg/dL  (0.8 a 2.32 mg/dL) | | 0.8 mg/dL  (0.30 a 1.30 mg/dL) | | 0.8 mg/dL  (0.30 a 1.30 mg/dL) | |
| **GGT** | 74 U/L  (47.7 a 86 UI/L) | | 77 U/L  (48 a 130 U/L) | | 64 U/L  (48 a 130 U/L) | |

| **AG125** | **NHPs Transfer** | | **-1d pre-challenge*** | | **7d post-challenge** | |
| --- | --- | --- | --- | --- | --- | --- |
| **Erythrocytes** | 6.46 millions/μl | | 5.31 millions/mm^3^ | | 4.82 millions/mm^3^ | |
| Hemoglobin | 14.6 g/dL | | 12.10 g/dL | | 10.90 g/dL | |
| Globular Volume | 45.0 % | |  | |  | |
| Hematocrit (HCT) |  | | 40.50 % | | 37.30 % | |
| Mean Corpuscular Volume (MCV) | 69.7 fl | | 76.30 fm^3^ | | 77.40 fm^3^ | |
| Mean Corpuscular Hemoglobin (MCH) |  | | 22.80 pg | | 22.60 pg | |
| Mean Corpuscular Hemoglobin Concentration (MCHC) | 32.4 % | | 29.90 g/dL | | 29.20 g/dL | |
| Note:  *Normocytic and normochromic erythrocytes. | | | | | | |
| **Leukocytes** | 6.9 x 10^3^/μl | | 6.30 x 10^3^/mm^3^ | | 4.10 x 10^3^/mm^3^ | |
|  | Relative (%) | Absolute (μL) | Relative (%) | Absolute (10^3^/mm^3^) | Relative (%) | Absolute (10^3^/mm^3^) |
| Basophils | 0 | 0 | 0 | 0 | 0 | 0 |
| Eosinophils | 2 | 138 | 2 | 0.02 | 0 | 0 |
| Myelocytes | 0 | 0 |  |  |  |  |
| Metamyelocytes | 0 | 0 |  |  |  |  |
| Neutrophils (Bands) | 0 | 0 | 60 | 0.60 | 0 | 0 |
| Neutrophils (Segmented) | 61 | 4209 | 37 | 0.37 | 54 | 2.21 |
| Lymphocytes | 34 | 2346 | 1 | 0.01 | 43 | 1.76 |
| Monocytes | 3 | 207 | 0 | 0 | 3 | 0.12 |
| **Platelets** | 550 x 10^3^/μl | | 394 x 10^3^/mm^3^ | | 327 x 10^3^/mm^3^ | |
| **ALT** | 45 UI/L  (0 a 82 UI/L) | | 29 U/L  (18 a 129 U/L) | | 84 U/L  (18 a 129 U/L) | |
| **AST** | 52 UI/L  (13 a 37 UI/L) | | 49 U/L  (23 a 70 U/L) | | 91 U/L  (23 a 70 U/L) | |
| **Creatinine** | 0.8 mg/dL  (0.8 a 2.32 mg/dL) | | 0.8 mg/dL  (0.30 a 1.30 mg/dL) | | 0.8 mg/dL  (0.30 a 1.30 mg/dL) | |
| **GGT** | 72 U/L  (47.7 a 86 UI/L) | | 65 U/L  (48 a 130 U/L) | | 75 U/L  (48 a 130 U/L) | |

| **AG135** | **NHPs Transfer*** | | **-1d pre-challenge**^#^ | | **7d post-challenge** | |
| --- | --- | --- | --- | --- | --- | --- |
| **Erythrocytes** | 5.64 millions/μl | | 5.20 millions/mm^3^ | | 4.73 millions/mm^3^ | |
| Hemoglobin | 12.4 g/dL | | 10.90 g/dL | | 10 g/dL | |
| Globular Volume | 37.1 % | |  | |  | |
| Hematocrit (HCT) |  | | 36.50 % | | 33.30 % | |
| Mean Corpuscular Volume (MCV) | 65.8 fl | | 70.20 fm^3^ | | 70.40 fm^3^ | |
| Mean Corpuscular Hemoglobin (MCH) |  | | 21 pg | | 21.10 pg | |
| Mean Corpuscular Hemoglobin Concentration (MCHC) | 33.4 % | | 29.90 g/dL | | 30 g/dL | |
| Note:  ^#^Normocytic and normochromic erythrocytes. | | | | | | |
| **Leukocytes** | 14.5 x 10^3^/μl | | 7.90 x 10^3^/mm^3^ | | 3.20 x 10^3^/mm^3^ | |
|  | Relative (%) | Absolute (μL) | Relative (%) | Absolute (10^3^/mm^3^) | Relative (%) | Absolute (10^3^/mm^3^) |
| Basophils | 0 | 0 | 0 | 0 | 0 | 0 |
| Eosinophils | 0 | 0 | 2 | 0.02 | 0 | 0 |
| Myelocytes | 0 | 0 |  |  |  |  |
| Metamyelocytes | 0 | 0 |  |  |  |  |
| Neutrophils (Bands) | 0 | 0 | 0 | 0 | 1 | 0.03 |
| Neutrophils (Segmented) | 75 | 10875 | 51 | 0.51 | 27 | 0.86 |
| Lymphocytes | 23 | 3335 | 45 | 0.45 | 64 | 2.05 |
| Monocytes | 2 | 290 | 2 | 0.02 | 7 | 0.22 |
| Note:  *Some reactive lymphocytes. | | | | | | |
| **Platelets** | 580 x 10^3^/μl | | 401 x 10^3^/mm^3^ | | 377 x 10^3^/mm^3^ | |
| Note:  *Some platelet aggregates. | | | | | | |
| **ALT** | 70 UI/L  (0 a 82 UI/L) | | 40 U/L  (18 a 129 U/L) | | 73 U/L  (18 a 129 U/L) | |
| **AST** | 47 UI/L  (13 a 37 UI/L) | | 46 U/L  (23 a 70 U/L) | | 97 U/L  (23 a 70 U/L) | |
| **Creatinine** | 1.2 mg/dL  (0.8 a 2.32 mg/dL) | | 0.7 mg/dL  (0.30 a 1.30 mg/dL) | | 0.9 mg/dL  (0.30 a 1.30 mg/dL) | |
| **GGT** | 101 U/L  (47.7 a 86 UI/L) | | 79 U/L  (48 a 130 U/L) | | 58 U/L  (48 a 130 U/L) | |

| **AG169** | **NHPs Transfer*** | | **-1d pre-challenge**^#^ | | **7d post-challenge** | |
| --- | --- | --- | --- | --- | --- | --- |
| **Erythrocytes (millions/μl)** | 5.64 millions/μl | | 5.14 millions/mm^3^ | | 4.44 millions/mm^3^ | |
| Hemoglobin (g/dL) | 12.6 g/dL | | 11.90 g/dL | | 10 g/dL | |
| Globular Volume (%) | 38.7 % | |  | |  | |
| Hematocrit (HCT) |  | | 37.90 % | | 33 % | |
| Mean Corpuscular Volume (MCV) | 68.6 fl | | 73.70 fm^3^ | | 74.30 fm^3^ | |
| Mean Corpuscular Hemoglobin (MCH) |  | | 23.20 pg | | 22.50 pg | |
| Mean Corpuscular Hemoglobin Concentration (MCHC) | 32.5 % | | 31.40 g/dL | | 30.30 g/dL | |
| Note:  ^#^Normocytic and normochromic erythrocytes. | | | | | | |
| **Leukocytes** | 10.4 x 10^3^/μl | | 14.60 x 10^3^/mm^3^ | | 4.10 x 10^3^/mm^3^ | |
|  | Relative (%) | Absolute (μL) | Relative (%) | Absolute (10^3^/mm^3^) | Relative (%) | Absolute (10^3^/mm^3^) |
| Basophils | 0 | 0 | 0 | 0 | 1 | 0.04 |
| Eosinophils | 0 | 0 | 0 | 0 | 0 | 0 |
| Myelocytes | 0 | 0 |  |  |  |  |
| Metamyelocytes | 0 | 0 |  |  |  |  |
| Neutrophils (Bands) | 0 | 0 | 0 | 0 | 2 | 0.08 |
| Neutrophils (Segmented) | 80 | 8320 | 80 | 0.80 | 66 | 2.71 |
| Lymphocytes | 14 | 1456 | 11 | 0.11 | 30 | 1.23 |
| Monocytes | 6 | 624 | 9 | 0.90 | 1 | 0.04 |
| **Platelets** | 541 x 10^3^/μl | | 360 x 10^3^/mm^3^ | | 372 x 10^3^/mm^3^ | |
| Note:  *Some platelet aggregates. | | | | | | |
| **ALT** | 28 UI/L  (0 a 82 UI/L) | | 29 U/L  (18 a 129 U/L) | | 55 U/L  (18 a 129 U/L) | |
| **AST** | 35 UI/L  (13 a 37 UI/L) | | 54 U/L  (23 a 70 U/L) | | 100 U/L  (23 a 70 U/L) | |
| **Creatinine** | 0.8 mg/dL  (0.8 a 2.32 mg/dL) | | 0.8 mg/dL  (0.30 a 1.30 mg/dL) | | 0.8 mg/dL  (0.30 a 1.30 mg/dL) | |
| **GGT** | 53 U/L  (47.7 a 86 UI/L) | | 72 U/L  (48 a 130 U/L) | | 54 U/L  (48 a 130 U/L) | |

| **AG177** | **NHPs Transfer** | | **-1d pre-challenge** | | **7d post-challenge** | |
| --- | --- | --- | --- | --- | --- | --- |
| **Erythrocytes (millions/μl)** | 6.10 millions/μl | | 5.29 millions/mm^3^ | | 4.36 millions/mm^3^ | |
| Hemoglobin (g/dL) | 13.9 g/dL | | 11.90 g/dL | | 9.70 g/dL | |
| Globular Volume (%) | 41.4 % | |  | |  | |
| Hematocrit (HCT) |  | | 38 % | | 31.60 % | |
| Mean Corpuscular Volume (MCV) | 67.9 fl | | 71.80 fm^3^ | | 72.50 fm^3^ | |
| Mean Corpuscular Hemoglobin (MCH) |  | | 22.50 pg | | 22.20 pg | |
| Mean Corpuscular Hemoglobin Concentration (MCHC) | 33.5 % | | 31.30 g/dL | | 30.70 g/dL | |
| **Leukocytes** | 8.6 x 10^3^/μl | | 10.70 x 10^3^/mm^3^ | | 3.30 x 10^3^/mm^3^ | |
|  | Relative (%) | Absolute (μL) | Relative (%) | Absolute (10^3^/mm^3^) | Relative (%) | Absolute (10^3^/mm^3^) |
| Basophils | 0 | 0 | 0 | 0 | 0 | 0 |
| Eosinophils | 10 | 860 | 1 | 0.01 | 0 | 0 |
| Myelocytes | 0 | 0 |  |  |  |  |
| Metamyelocytes | 0 | 0 |  |  |  |  |
| Neutrophils (Bands) | 0 | 0 | 1 | 0.01 | 2 | 0.07 |
| Neutrophils (Segmented) | 64 | 5504 | 72 | 0.72 | 59 | 1.95 |
| Lymphocytes | 24 | 2064 | 21 | 0.21 | 36 | 1.19 |
| Monocytes | 2 | 172 | 5 | 0.05 | 3 | 0.10 |
| **Platelets** | 574 x 10^3^/μl | | 363 x 10^3^/mm^3^ | | 384 x 10^3^/mm^3^ | |
| **ALT** | 29 UI/L  (0 a 82 UI/L) | | 21 U/L  (18 a 129 U/L) | | 35 U/L  (18 a 129 U/L) | |
| **AST** | 42 UI/L  (13 a 37 UI/L) | | 51 U/L  (23 a 70 U/L) | | 57 U/L  (23 a 70 U/L) | |
| **Creatinine** | 0.8 mg/dL  (0.8 a 2.32 mg/dL) | | 0.8 mg/dL  (0.30 a 1.30 mg/dL) | | 0.5 mg/dL  (0.30 a 1.30 mg/dL) | |
| **GGT** | 68 U/L  (47.7 a 86 UI/L) | | 67 U/L  (48 a 130 U/L) | | 48 U/L  (48 a 130 U/L) | |

**Group 2**

| **AE87** | **NHPs Transfer*** | | **-1d pre-challenge^#^** | | **7d post-challenge** | |
| --- | --- | --- | --- | --- | --- | --- |
| **Erythrocytes** | 6.51 millions/μl | | 6.17 millions/mm^3^ | | 4.96 millions/mm^3^ | |
| Hemoglobin | 14.6 g/dL | | 13.40 g/dL | | 11.10 g/dL | |
| Globular Volume | 44.4 % | |  | |  | |
| Hematocrit (HCT) |  | | 44.30 % | | 35.60 % | |
| Mean Corpuscular Volume (MCV) | 68.2 fl | | 71.80 fm^3^ | | 71.80 fm^3^ | |
| Mean Corpuscular Hemoglobin (MCH) |  | | 21.70 pg | | 22.40 pg | |
| Mean Corpuscular Hemoglobin Concentration (MCHC) | 32.8 % | | 30.20 g/dL | | 31.20 g/dL | |
| Note:  **^#^**Normocytic and normochromic erythrocytes. | | | | | | |
| **Leukocytes** | 9.5 x 10^3^/μl | | 7.70 x 10^3^/mm^3^ | | 4.50 x 10^3^/mm^3^ | |
|  | Relative (%) | Absolute (μL) | Relative (%) | Absolute (mil/mm^3^) | Relative (%) | Absolute (mil/mm^3^) |
| Basophils | 0 | 0 | 1 | 0.01 | 0 | 0 |
| Eosinophils | 0 | 0 | 0 | 0 | 0 | 0 |
| Myelocytes | 0 | 0 |  |  |  |  |
| Metamyelocytes | 0 | 0 |  |  |  |  |
| Neutrophils (Bands) | 0 | 0 | 0 | 0 | 0 | 0 |
| Neutrophils (Segmented) | 66 | 6270 | 56 | 0.56 | 53 | 2.38 |
| Lymphocytes | 32 | 3040 | 40 | 0.40 | 42 | 1.89 |
| Monocytes | 2 | 190 | 3 | 0.03 | 5 | 0.22 |
| Note:  *Some reactive lymphocytes. | | | | | | |
| **Platelets** | 480 x 10^3^/μl | | 293 x 10^3^/mm^3^ | | 185 x 10^3^/mm^3^ | |
| **ALT** | 25 UI/L  (0 a 82 UI/L) | | 17 U/L  (18 a 129 U/L) | | 44 U/L  (18 a 129 U/L) | |
| **AST** | 47 UI/L  (13 a 37 UI/L) | | 43 U/L  (23 a 70 U/L) | | 98 U/L  (23 a 70 U/L) | |
| **Creatinine** | 1.4 mg/dL  (0.8 a 2.32 mg/dL) | | 1.2 mg/dL  (0.30 a 1.30 mg/dL) | | 1.3 mg/dL  (0.30 a 1.30 mg/dL) | |
| **GGT** | 69 U/L  (47.7 a 86 UI/L) | | 74 U/L  (48 a 130 U/L) | | 84 U/L  (48 a 130 U/L) | |

| **AE107** | **NHPs Transfer** | | **-1d pre-challenge*** | | **7d post-challenge** | |
| --- | --- | --- | --- | --- | --- | --- |
| **Erythrocytes** | 6.94 millions/μl | | 6.41 millions/mm^3^ | | 5.50 millions/mm^3^ | |
| Hemoglobin | 16.2 g/dL | | 13.70 g/dL | | 11.60 g/dL | |
| Globular Volume | 47.9 % | |  | |  | |
| Hematocrit (HCT) |  | | 45.10 % | | 38.70 % | |
| Mean Corpuscular Volume (MCV) | 69.0 fl | | 70.40 fm^3^ | | 70.40 fm^3^ | |
| Mean Corpuscular Hemoglobin (MCH) |  | | 21.40 pg | | 21.10 pg | |
| Mean Corpuscular Hemoglobin Concentration (MCHC) | 33.8 % | | 30.40 g/dL | | 30 g/dL | |
| Note:  **^*^**Normocytic and normochromic erythrocytes. | | | | | | |
| **Leukocytes** | 11.4 x 10^3^/μl | | 8.20 x 10^3^/mm^3^ | | 5.50 x 10^3^/mm^3^ | |
|  | Relative (%) | Absolute (μL) | Relative (%) | Absolute (mil/mm^3^) | Relative (%) | Absolute (mil/mm^3^) |
| Basophils | 0 | 0 | 0 | 0 | 0 | 0 |
| Eosinophils | 0 | 0 | 0 | 0 | 0 | 0 |
| Myelocytes | 0 | 0 |  |  |  |  |
| Metamyelocytes | 0 | 0 |  |  |  |  |
| Neutrophils (Bands) | 0 | 0 | 1 | 0.01 | 1 | 0.06 |
| Neutrophils (Segmented) | 72 | 8208 | 59 | 0.59 | 56 | 3.08 |
| Lymphocytes | 20 | 2280 | 30 | 0.30 | 38 | 2.09 |
| Monocytes | 8 | 912 | 10 | 0.10 | 5 | 0.28 |
| **Platelets** | 548 x 10^3^/μl | | 558 x 10^3^/mm^3^ | | 668 x 10^3^/mm^3^ | |
| **ALT** | 25 UI/L  (0 a 82 UI/L) | | 11 U/L  (18 a 129 U/L) | | 22 U/L  (18 a 129 U/L) | |
| **AST** | 46 UI/L  (13 a 37 UI/L) | | 48 U/L  (23 a 70 U/L) | | 66 U/L  (23 a 70 U/L) | |
| **Creatinine** | 1.4 mg/dL  (0.8 a 2.32 mg/dL) | | 0.7 mg/dL  (0.30 a 1.30 mg/dL) | | 0.8 mg/dL  (0.30 a 1.30 mg/dL) | |
| **GGT** | 71 U/L  (47.7 a 86 UI/L) | | 64 U/L  (48 a 130 U/L) | | 58 U/L  (48 a 130 U/L) | |

| **AF50** | **NHPs Transfer** | | **-1d pre-challenge*** | | **7d post-challenge** | |
| --- | --- | --- | --- | --- | --- | --- |
| **Erythrocytes** | 5.96 millions/μl | | 4.96 millions/mm^3^ | | 4.61 millions/mm^3^ | |
| Hemoglobin | 14.2 g/dL | | 11.30 g/dL | | 10.50 g/dL | |
| Globular Volume | 42.3 % | |  | |  | |
| Hematocrit (HCT) |  | | 36.90 % | | 4.20 % | |
| Mean Corpuscular Volume (MCV) | 71.0 fl | | 74.40 fm^3^ | | 74.20 fm^3^ | |
| Mean Corpuscular Hemoglobin (MCH) |  | | 22.80 pg | | 22.80 pg | |
| Mean Corpuscular Hemoglobin Concentration (MCHC) | 33.5 % | | 30.60 g/dL | | 30.70 g/dL | |
| Note:  **^*^**Normocytic and normochromic erythrocytes. | | | | | | |
| **Leukocytes** | 8.5 x 10^3^/μl | | 6.40 x 10^3^/mm^3^ | | 3.40 x 10^3^/mm^3^ | |
|  | Relative (%) | Absolute (μL) | Relative (%) | Absolute (mil/mm^3^) | Relative (%) | Absolute (mil/mm^3^) |
| Basophils | 0 | 0 | 0 | 0 | 0 | 0 |
| Eosinophils | 10 | 850 | 0 | 0 | 1 | 0.03 |
| Myelocytes | 0 | 0 |  |  |  |  |
| Metamyelocytes | 0 | 0 |  |  |  |  |
| Neutrophils (Bands) | 0 | 0 | 0 | 0 | 0 | 0 |
| Neutrophils (Segmented) | 20 | 1700 | 63 | 0.63 | 45 | 1.53 |
| Lymphocytes | 64 | 5440 | 36 | 0.36 | 48 | 1.63 |
| Monocytes | 6 | 510 | 1 | 0.01 | 6 | 0.20 |
| **Platelets** | 508 x 10^3^/μl | | 327 x 10^3^/mm^3^ | | 374 x 10^3^/mm^3^ | |
| **ALT** | 22 UI/L  (0 a 82 UI/L) | | 27 U/L  (18 a 129 U/L) | | 60 U/L  (18 a 129 U/L) | |
| **AST** | 42 UI/L  (13 a 37 UI/L) | | 49 U/L  (23 a 70 U/L) | | 91 U/L  (23 a 70 U/L) | |
| **Creatinine** | 0.8 mg/dL  (0.8 a 2.32 mg/dL) | | 0.6 mg/dL  (0.30 a 1.30 mg/dL) | | 0.7 mg/dL  (0.30 a 1.30 mg/dL) | |
| **GGT** | 56 U/L  (47.7 a 86 UI/L) | | 53 U/L  (48 a 130 U/L) | | 47 U/L  (48 a 130 U/L) | |

| **AF122** | **NHPs Transfer** | | **-1d pre-challenge*** | | **7d post-challenge** | |
| --- | --- | --- | --- | --- | --- | --- |
| **Erythrocytes** | 4.90 millions/μl | | 5.12 millions/mm^3^ | | 4.70 millions/mm^3^ | |
| Hemoglobin | 10.8 g/dL | | 10.30 g/dL | | 9.60 g/dL | |
| Globular Volume | 32.1 % | |  | |  | |
| Hematocrit (HCT) |  | | 34.30 % | | 31.70 % | |
| Mean Corpuscular Volume (MCV) | 65.5 fl | | 67 fm^3^ | | 67.40 fm^3^ | |
| Mean Corpuscular Hemoglobin (MCH) |  | | 20.10 pg | | 20.40 pg | |
| Mean Corpuscular Hemoglobin Concentration (MCHC) | 33.6 % | | 30 g/dL | | 30.30 g/dL | |
| Note:  **^*^**Normocytic and normochromic erythrocytes. | | | | | | |
| **Leukocytes** | 5.7 x 10^3^/μl | | 10 mil/mm3 | | 2.30 mil/mm3 | |
|  | Relative (%) | Absolute (μL) | Relative (%) | Absolute (mil/mm^3^) | Relative (%) | Absolute (mil/mm^3^) |
| Basophils | 0 | 0 | 0 | 0 | 0 | 0 |
| Eosinophils | 0 | 0 | 0 | 0 | 1 | 0.02 |
| Myelocytes | 0 | 0 |  |  |  |  |
| Metamyelocytes | 0 | 0 |  |  |  |  |
| Neutrophils (Bands) | 0 | 0 | 0 | 0 | 0 | 0 |
| Neutrophils (Segmented) | 60 | 3420 | 79 | 0.79 | 42 | 0.97 |
| Lymphocytes | 36 | 2052 | 20 | 0.20 | 56 | 1.29 |
| Monocytes | 4 | 228 | 1 | 0.01 | 1 | 0.02 |
| **Platelets** | 609 x 10^3^/μl | | 479 x 10^3^/mm^3^ | | 444 x 10^3^/mm^3^ | |
| **ALT** | 19 UI/L  (0 a 82 UI/L) | | 17 U/L  (18 a 129 U/L) | | 48 U/L  (18 a 129 U/L) | |
| **AST** | 30 UI/L  (13 a 37 UI/L) | | 63 U/L  (23 a 70 U/L) | | 50 U/L  (23 a 70 U/L) | |
| **Creatinine** | 0.7 mg/dL  (0.8 a 2.32 mg/dL) | | 0.6 mg/dL  (0.30 a 1.30 mg/dL) | | 0.7 mg/dL  (0.30 a 1.30 mg/dL) | |
| **GGT** | 68 U/L  (47.7 a 86 UI/L) | | 70 U/L  (48 a 130 U/L) | | 65 U/L  (48 a 130 U/L) | |

| **AF167** | **NHPs Transfer** | | **-1d pre-challenge*** | | **7d post-challenge** | |
| --- | --- | --- | --- | --- | --- | --- |
| **Erythrocytes** | 6.67 millions/μl | | 5.72 millions/mm^3^ | | 5.03 millions/mm^3^ | |
| Hemoglobin | 13.8 g/dL | | 11.80 g/dL | | 10.50 g/dL | |
| Globular Volume | 41.8 % | |  | |  | |
| Hematocrit (HCT) |  | | 38.50 % | | 34.40 % | |
| Mean Corpuscular Volume (MCV) | 62.7 fl | | 67.30 fm^3^ | | 68.40 fm^3^ | |
| Mean Corpuscular Hemoglobin (MCH) |  | | 20.60 pg | | 20.90 pg | |
| Mean Corpuscular Hemoglobin Concentration (MCHC) | 33.0 % | | 30.60 g/dL | | 30.50 g/dL | |
| Note:  **^*^**Normocytic and normochromic erythrocytes. | | | | | | |
| **Leukocytes** | 7.7 x 10^3^/μl | | 5.30 x 10^3^/mm^3^ | | 3.60 x 10^3^/mm^3^ | |
|  | Relative (%) | Absolute (μL) | Relative (%) | Absolute (mil/mm^3^) | Relative (%) | Absolute (mil/mm^3^) |
| Basophils | 0 | 0 | 0 | 0 | 0 | 0 |
| Eosinophils | 2 | 154 | 0 | 0 | 1 | 0.04 |
| Myelocytes | 0 | 0 |  |  |  |  |
| Metamyelocytes | 0 | 0 |  |  |  |  |
| Neutrophils (Bands) | 2 | 154 | 0 | 0 | 1 | 0.04 |
| Neutrophils (Segmented) | 68 | 5236 | 61 | 0.61 | 62 | 2.23 |
| Lymphocytes | 24 | 1848 | 34 | 1.80 | 34 | 1.22 |
| Monocytes | 4 | 308 | 5 | 0.26 | 2 | 0.07 |
| **Platelets** | 540 x 10^3^/μl | | 449 x 10^3^/mm^3^ | | 453 x 10^3^/mm^3^ | |
| **ALT** | 59 UI/L  (0 a 82 UI/L) | | 24 U/L  (18 a 129 U/L) | | 30 U/L  (18 a 129 U/L) | |
| **AST** | 51 UI/L  (13 a 37 UI/L) | | 36 U/L  (23 a 70 U/L) | | 66 U/L  (23 a 70 U/L) | |
| **Creatinine** | 0.8 mg/dL  (0.8 a 2.32 mg/dL) | | 0.7 mg/dL  (0.30 a 1.30 mg/dL) | | 0.9 mg/dL  (0.30 a 1.30 mg/dL) | |
| **GGT** | 87 U/L  (47.7 a 86 UI/L) | | 95 U/L  (48 a 130 U/L) | | 87 U/L  (48 a 130 U/L) | |

| **AG35** | **NHPs Transfer** | | **-1d pre-challenge*** | | **7d post-challenge** | |
| --- | --- | --- | --- | --- | --- | --- |
| **Erythrocytes** | 6.54 millions/μl | | 5.42 millions/mm^3^ | | 4.98 millions/mm^3^ | |
| Hemoglobin | 14 g/dL | | 11.70 g/dL | | 10.50 g/dL | |
| Globular Volume | 43.2 % | |  | |  | |
| Hematocrit (HCT) |  | | 38.50 % | | 35.90 % | |
| Mean Corpuscular Volume (MCV) | 66.1 fl | | 71 fm^3^ | | 72.10 fm^3^ | |
| Mean Corpuscular Hemoglobin (MCH) |  | | 21.60 pg | | 21.10 pg | |
| Mean Corpuscular Hemoglobin Concentration (MCHC) | 32.4 % | | 30.40 g/dL | | 29.20 g/dL | |
| Note:  **^*^**Normocytic and normochromic erythrocytes. | | | | | | |
| **Leukocytes** | 8.3 x 10^3^/μl | | 5.30 x 10^3^/mm^3^ | | 4.10 x 10^3^/mm^3^ | |
|  | Relative (%) | Absolute (μL) | Relative (%) | Absolute (mil/mm^3^) | Relative (%) | Absolute (mil/mm^3^) |
| Basophils | 0 | 0 | 1 | 0.05 | 0 | 0 |
| Eosinophils | 0 | 0 | 0 | 0 | 1 | 0.04 |
| Myelocytes | 0 | 0 |  |  |  |  |
| Metamyelocytes | 0 | 0 |  |  |  |  |
| Neutrophils (Bands) | 0 | 0 | 0 | 0 | 3 | 0.12 |
| Neutrophils (Segmented) | 80 | 6640 | 59 | 3.13 | 32 | 1.31 |
| Lymphocytes | 20 | 1660 | 34 | 1.80 | 56 | 2.30 |
| Monocytes | 0 | 0 | 7 | 0.37 | 8 | 0.33 |
| **Platelets** | 600 x 10^3^/μl | | 644 x 10^3^/mm^3^ | | 631 x 10^3^/mm^3^ | |
| **ALT** | 26 UI/L  (0 a 82 UI/L) | | 11 U/L  (18 a 129 U/L) | | 57 U/L  (18 a 129 U/L) | |
| **AST** | 38 UI/L  (13 a 37 UI/L) | | 50 U/L  (23 a 70 U/L) | | 76 U/L  (23 a 70 U/L) | |
| **Creatinine** | 0.9 mg/dL  (0.8 a 2.32 mg/dL) | | 0.7 mg/dL  (0.30 a 1.30 mg/dL) | | 0.7 mg/dL  (0.30 a 1.30 mg/dL) | |
| **GGT** | 74 U/L  (47.7 a 86 UI/L) | | 90 U/L  (48 a 130 U/L) | | 49 U/L  (48 a 130 U/L) | |

| **AG77** | **NHPs Transfer** | | **-1d pre-challenge*** | | **7d post-challenge** | |
| --- | --- | --- | --- | --- | --- | --- |
| **Erythrocytes** | 6.21 millions/μl | | 5.09 millions/mm^3^ | | 5.80 millions/mm^3^ | |
| Hemoglobin | 14.2 g/dL | | 11.50 g/dL | | 11.40 g/dL | |
| Globular Volume | 43.4 % | |  | |  | |
| Hematocrit (HCT) |  | | 37.10 % | | 37.60 % | |
| Mean Corpuscular Volume (MCV) | 69.9 fl | | 72.90 fm^3^ | | 74 fm^3^ | |
| Mean Corpuscular Hemoglobin (MCH) |  | | 22.60 pg | | 22.40 pg | |
| Mean Corpuscular Hemoglobin Concentration (MCHC) | 32.7 % | | 31 g/dL | | 30.30 g/dL | |
| Note:  **^*^**Normocytic and normochromic erythrocytes. | | | | | | |
| **Leukocytes** | 8.2 x 10^3^/μl | | 8.70 x 10^3^/mm^3^ | | 5.40 x 10^3^/mm^3^ | |
|  | Relative (%) | Absolute (μL) | Relative (%) | Absolute (mil/mm^3^) | Relative (%) | Absolute (mil/mm^3^) |
| Basophils | 0 | 0 | 0 | 0 | 1 | 0.05 |
| Eosinophils | 2 | 164 | 2 | 0.17 | 0 | 0 |
| Myelocytes | 0 | 0 |  |  |  |  |
| Metamyelocytes | 0 | 0 |  |  |  |  |
| Neutrophils (Bands) | 0 | 0 | 0 | 0 | 1 | 0.05 |
| Neutrophils (Segmented) | 63 | 5166 | 49 | 4.26 | 41 | 2.21 |
| Lymphocytes | 33 | 2706 | 49 | 4.26 | 46 | 2.48 |
| Monocytes | 2 | 164 | 0 | 0 | 11 | 0.59 |
| **Platelets** | 500 x 10^3^/μl | | 333 x 10^3^/mm^3^ | | 323 x 10^3^/mm^3^ | |
| **ALT** | 28 UI/L  (0 a 82 UI/L) | | 24 U/L  (18 a 129 U/L) | | 36 U/L  (18 a 129 U/L) | |
| **AST** | 37 UI/L  (13 a 37 UI/L) | | 38 U/L  (23 a 70 U/L) | | 61 U/L  (23 a 70 U/L) | |
| **Creatinine** | 0.8 mg/dL  (0.8 a 2.32 mg/dL) | | 0.6 mg/dL  (0.30 a 1.30 mg/dL) | | 0.7 mg/dL  (0.30 a 1.30 mg/dL) | |
| **GGT** | 78 U/L  (47.7 a 86 UI/L) | | 102 U/L  (48 a 130 U/L) | | 79 U/L  (48 a 130 U/L) | |

| **AG117** | **NHPs Transfer*** | | **-1d pre-challenge^#^** | | **7d post-challenge** | |
| --- | --- | --- | --- | --- | --- | --- |
| **Erythrocytes** | 5.82 millions/μl | | 5.10 millions/mm^3^ | | 4.18 millions/mm^3^ | |
| Hemoglobin | 13.4 g/dL | | 11.50 g/dL | | 9.60 g/dL | |
| Globular Volume | 39.6 % | |  | |  | |
| Hematocrit (HCT) |  | | 37 % | | 31.70 % | |
| Mean Corpuscular Volume (MCV) | 68.0 fl | | 72.50 fm^3^ | | 75.80 fm^3^ | |
| Mean Corpuscular Hemoglobin (MCH) |  | | 22.50 pg | | 23 pg | |
| Mean Corpuscular Hemoglobin Concentration (MCHC) | 33.8 % | | 31.10 g/dL | | 30.30 g/dL | |
| Note:  **^#^**Normocytic and normochromic erythrocytes. | | | | | | |
| **Leukocytes** | 10.8 x 10^3^/μl | | 10.40 x 10^3^/mm^3^ | | 4.30 x 10^3^/mm^3^ | |
|  | Relative (%) | Absolute (μL) | Relative (%) | Absolute (mil/mm^3^) | Relative (%) | Absolute (mil/mm^3^) |
| Basophils | 0 | 0 | 0 | 0 | 0 | 0 |
| Eosinophils | 0 | 0 | 1 | 0.01 | 0 | 0 |
| Myelocytes | 0 | 0 |  |  |  |  |
| Metamyelocytes | 0 | 0 |  |  |  |  |
| Neutrophils (Bands) | 0 | 0 | 0 | 0 | 1 | 0.04 |
| Neutrophils (Segmented) | 55 | 5940 | 79 | 0.79 | 65 | 2.80 |
| Lymphocytes | 45 | 4860 | 19 | 0.19 | 33 | 1.42 |
| Monocytes | 0 | 0 | 1 | 0.01 | 1 | 0.04 |
| Morpholy and Note:  Some reative lymphocytes. | | | | | | |
| **Platelets** | 460 x 10^3^/μl | | 347 x 10^3^/mm^3^ | | 275 x 10^3^/mm^3^ | |
| **ALT** | 31 UI/L  (0 a 82 UI/L) | | 58 U/L  (18 a 129 U/L) | | 62 U/L  (18 a 129 U/L) | |
| **AST** | 40 UI/L  (13 a 37 UI/L) | | 48 U/L  (23 a 70 U/L) | | 92 U/L  (23 a 70 U/L) | |
| **Creatinine** | 0.8 mg/dL  (0.8 a 2.32 mg/dL) | | 0.7 mg/dL  (0.30 a 1.30 mg/dL) | | 0.9 mg/dL  (0.30 a 1.30 mg/dL) | |
| **GGT** | 88 U/L  (47.7 a 86 UI/L) | | 105 U/L  (48 a 130 U/L) | | 74 U/L  (48 a 130 U/L) | |

| **AH44** | **NHPs Transfer** | | **-1d pre-challenge*** | | **7d post-challenge** | |
| --- | --- | --- | --- | --- | --- | --- |
| **Erythrocytes** | 6.19 millions/μl | | 5.58 millions/mm^3^ | | 5.42 millions/mm^3^ | |
| Hemoglobin | 14.5 g/dL | | 12.80 g/dL | | 12.20 g/dL | |
| Globular Volume | 43.0 % | |  | |  | |
| Hematocrit (HCT) |  | | 41.90 % | | 40.70 % | |
| Mean Corpuscular Volume (MCV) | 69.5 fl | | 75.10 fm^3^ | | 75.10 fm^3^ | |
| Mean Corpuscular Hemoglobin (MCH) |  | | 22.90 pg | | 22.50 pg | |
| Mean Corpuscular Hemoglobin Concentration (MCHC) | 33.7 % | | 30.50 g/dL | | 30 g/dL | |
| Note:  **^*^**Normocytic and normochromic erythrocytes. | | | | | | |
| **Leukocytes** | 11.0 x 10^3^/μl | | 5.20 x 10^3^/mm^3^ | | 3.80 x 10^3^/mm^3^ | |
|  | Relative (%) | Absolute (μL) | Relative (%) | Absolute (mil/mm^3^) | Relative (%) | Absolute (mil/mm^3^) |
| Basophils | 0 | 0 | 0 | 0 | 1 | 0.04 |
| Eosinophils | 0 | 0 | 1 | 0.01 | 0 | 0 |
| Myelocytes | 0 | 0 |  |  |  |  |
| Metamyelocytes | 0 | 0 |  |  |  |  |
| Neutrophils (Bands) | 0 | 0 | 0 | 0 | 0 | 0 |
| Neutrophils (Segmented) | 82 | 9020 | 39 | 0.39 | 54 | 2.05 |
| Lymphocytes | 18 | 1980 | 55 | 0.55 | 37 | 1.41 |
| Monocytes | 0 | 0 | 5 | 0.05 | 8 | 0.30 |
| **Platelets** | 446 x 10^3^/μl | | 318 mil/mm3 | | 379 mil/mm3 | |
| **ALT** | 32 UI/L  (0 a 82 UI/L) | | 13 U/L  (18 a 129 U/L) | | 46 U/L  (18 a 129 U/L) | |
| **AST** | 33 UI/L  (13 a 37 UI/L) | | 48 U/L  (23 a 70 U/L) | | 72 U/L  (23 a 70 U/L) | |
| **Creatinine** | 0.7 mg/dL  (0.8 a 2.32 mg/dL) | | 0.7 mg/dL  (0.30 a 1.30 mg/dL) | | 0.8 mg/dL  (0.30 a 1.30 mg/dL) | |
| **GGT** | 69 U/L  (47.7 a 86 UI/L) | | 60 U/L  (48 a 130 U/L) | | 59 U/L  (48 a 130 U/L) | |

| **AH69** | **NHPs Transfer*** | | **-1d pre-challenge^#^** | | **7d post-challenge** | |
| --- | --- | --- | --- | --- | --- | --- |
| **Erythrocytes** | 6.51 millions/μl | | 5.58 millions/mm^3^ | | 4.86 millions/mm^3^ | |
| Hemoglobin | 14.5 g/dL | | 12.10 g/dL | | 10.80 g/dL | |
| Globular Volume | 43.7 % | |  | |  | |
| Hematocrit (HCT) |  | | 40 % | | 35.60 % | |
| Mean Corpuscular Volume (MCV) | 67.1 fl | | 71.70 fm^3^ | | 73.30 fm^3^ | |
| Mean Corpuscular Hemoglobin (MCH) |  | | 21.70 pg | | 22.20 pg | |
| Mean Corpuscular Hemoglobin Concentration (MCHC) | 33.1 % | | 30.30 g/dL | | 30.30 g/dL | |
| Note:  **^#^**Normocytic and normochromic erythrocytes. | | | | | | |
| **Leukocytes** | 9.2 x 10^3^/μl | | 7.50 x 10^3^/mm^3^ | | 3.80 x 10^3^/mm^3^ | |
|  | Relative (%) | Absolute (μL) | Relative (%) | Absolute (mil/mm^3^) | Relative (%) | Absolute (mil/mm^3^) |
| Basophils | 0 | 0 | 2 | 0.02 | 1 | 0.04 |
| Eosinophils | 0 | 0 | 1 | 0.01 | 0 | 0 |
| Myelocytes | 0 | 0 |  |  |  |  |
| Metamyelocytes | 0 | 0 |  |  |  |  |
| Neutrophils (Bands) | 0 | 0 | 0 | 0 | 1 | 0.04 |
| Neutrophils (Segmented) | 54 | 4968 | 45 | 0.45 | 50 | 1.90 |
| Lymphocytes | 46 | 4232 | 50 | 0.50 | 38 | 1.44 |
| Monocytes | 0 | 0 | 2 | 0.02 | 10 | 0.38 |
| **Platelets** | 469 x 10^3^/μl | | 286 x 10^3^/mm^3^ | | 230 x 10^3^/mm^3^ | |
| Note:  *Some platelet aggregates. | | | | | | |
| **ALT** | 31 UI/L  (0 a 82 UI/L) | | 15 U/L  (18 a 129 U/L) | | 42 U/L  (18 a 129 U/L) | |
| **AST** | 40 UI/L  (13 a 37 UI/L) | | 44 U/L  (23 a 70 U/L) | | 82 U/L  (23 a 70 U/L) | |
| **Creatinine** | 0.8 mg/dL  (0.8 a 2.32 mg/dL) | | 0.8 mg/dL  (0.30 a 1.30 mg/dL) | | 0.8 mg/dL  (0.30 a 1.30 mg/dL) | |
| **GGT** | 62 U/L  (47.7 a 86 UI/L) | | 70 U/L  (48 a 130 U/L) | | 61 U/L  (48 a 130 U/L) | |

**Group 3**

| **AG122** | **NHPs Transfer** | | **-1d pre-challenge** | | **7d post-challenge** | |
| --- | --- | --- | --- | --- | --- | --- |
| **Erythrocytes** | 4.90 millions/mm^3^ | | 5.04 millions/mm^3^ | | 4.45 millions/mm^3^ | |
| Hemoglobin | 10.30 g/dL | | 11.10 g/dL | | 8.63 g/dL | |
| Hematocrit (HCT) | 32.30 % | | 32.80 % | | 27.30 % | |
| Mean Corpuscular Volume (MCV) | 65.90 fm^3^ | | 65.07 fm^3^ | | 61.34 fm^3^ | |
| Mean Corpuscular Hemoglobin (MCH) | 21 pg | | 22.02 pg | | 19.39 pg | |
| Mean Corpuscular Hemoglobin Concentration (MCHC) | 31.90 g/dL | | 33.84 g/dL | | 31.61 g/dL | |
| **Leukocytes** | 10.90 x 10^3^/mm^3^ | | 8.70 x 10^3^/mm^3^ | | 4.73 x 10^3^/mm^3^ | |
|  | Relative (%) | Absolute (10^3^/mm^3^) | Relative (%) | Absolute (10^3^/mm^3^) | Relative (%) | Absolute (10^3^/mm^3^) |
| Basophils | 0 | 0 |  |  | 1 | 0.05 |
| Eosinophils | 0 | 0 | 1 | 0.09 | 2 | 0.10 |
| Neutrophils (Bands) | 1 | 0.11 |  |  |  |  |
| Neutrophils (Segmented) | 34 | 3.71 | 52 | 4.52 | 64 | 3.03 |
| Lymphocytes | 60 | 6.54 | 44 | 3.83 | 28 | 1.32 |
| Monocytes | 5 | 0.54 | 2 | 0.17 | 4 | 0.19 |
| **Platelets** | 563 x 10^3^/mm^3^ | | 510 x 10^3^/mm^3^ | | 447 x 10^3^/mm^3^ | |
| **ALT** | 33 U/L  (18 a 129 U/L) | | 26.83 U/L  (18 a 129 U/L) | | 68.26 U/L  (18 a 129 U/L) | |
| **AST** | 47 U/L  (23 a 70 U/L) | | 39.69 U/L  (23 a 70 U/L) | | 51.95 U/L  (23 a 70 U/L) | |
| **Creatinine** | 6.0 mg/dL  (0.30 a 1.30 mg/dL) | | 0.56 mg/dL  (0.30 a 1.30 mg/dL) | | 0.75 mg/dL  (0.30 a 1.30 mg/dL) | |
| **GGT** | 42 U/L  (48 a 130 U/L) | | 61.87 U/L  (48 a 130 U/L) | | 51.19 U/L  (48 a 130 U/L) | |

| **AG10** | **NHPs Transfer** | | **-1d pre-challenge** | | **7d post-challenge** | |
| --- | --- | --- | --- | --- | --- | --- |
| **Erythrocytes** | 4.90 millions/mm^3^ | | 4.44 millions/mm^3^ | | 4.34 millions/mm^3^ | |
| Hemoglobin | 10.90 g/dL | | 7.90 g/dL | | 6.81 g/dL | |
| Hematocrit (HCT) | 34.30 % | | 24.90 % | | 22.50 % | |
| Mean Corpuscular Volume (MCV) | 70 fm^3^ | | 56.08 fm^3^ | | 51.84 fm^3^ | |
| Mean Corpuscular Hemoglobin (MCH) | 22.20 pg | | 17.79 pg | | 15.69 pg | |
| Mean Corpuscular Hemoglobin Concentration (MCHC) | 31.80 g/dL | | 31.72 g/dL | | 30.26 g/dL | |
| **Leukocytes** | 7.40 x 10^3^/mm^3^ | | 5.80 x 10^3^/mm^3^ | | 4.22 x 10^3^/mm^3^ | |
|  | Relative (%) | Absolute (10^3^/mm^3^) | Relative (%) | Absolute (10^3^/mm^3^) | Relative (%) | Absolute (10^3^/mm^3^) |
| Basophils | 0 | 0 |  |  |  |  |
| Eosinophils | 0 | 0 | 0 | 0 | 2 | 0.08 |
| Neutrophils (Bands) | 2 | 0.15 |  |  |  |  |
| Neutrophils (Segmented) | 45 | 3.33 | 53 | 3.07 | 64 | 2.70 |
| Lymphocytes | 46 | 3.40 | 38 | 2.20 | 28 | 1.18 |
| Monocytes | 7 | 0.52 | 9 | 0.52 | 6 | 0.25 |
| **Platelets** | 504 x 10^3^/mm^3^ | | 666 x 10^3^/mm^3^ | | 462 x 10^3^/mm^3^ | |
| **ALT** | 28 U/L  (18 a 129 U/L) | | 14.55 U/L  (18 a 129 U/L) | | 41.83 U/L  (18 a 129 U/L) | |
| **AST** | 46 U/L  (23 a 70 U/L) | | 48.63 U/L  (23 a 70 U/L) | | 40.90 U/L  (23 a 70 U/L) | |
| **Creatinine** | 0.5 mg/dL  (0.30 a 1.30 mg/dL) | | 0.42 mg/dL  (0.30 a 1.30 mg/dL) | | 0.63 mg/dL  (0.30 a 1.30 mg/dL) | |
| **GGT** | 46 U/L  (48 a 130 U/L) | | 48.56 U/L  (48 a 130 U/L) | | 29.20 U/L  (48 a 130 U/L) | |

| **AH7** | **NHPs Transfer** | | **-1d pre-challenge** | | **7d post-challenge** | |
| --- | --- | --- | --- | --- | --- | --- |
| **Erythrocytes** | 6.21 millions/mm^3^ | | 4.04 millions/mm^3^ | | 5.03 millions/mm^3^ | |
| Hemoglobin | 12.10 g/dL | | 6.70 g/dL | | 6.69 g/dL | |
| Hematocrit (HCT) | 37.30 % | | 21.30 % | | 22.20 % | |
| Mean Corpuscular Volume (MCV) | 60.10 fm^3^ | | 52.72 fm^3^ | | 44.13 fm^3^ | |
| Mean Corpuscular Hemoglobin (MCH) | 19.50 pg | | 16.58 pg | | 13.30 pg | |
| Mean Corpuscular Hemoglobin Concentration (MCHC) | 32.40 g/dL | | 31.45 g/dL | | 30.13 g/dL | |
| **Leukocytes** | 12.30 x 10^3^/mm^3^ | | 10.0 x 10^3^/mm^3^ | | 8.73 x 10^3^/mm^3^ | |
|  | Relative (%) | Absolute (10^3^/mm^3^) | Relative (%) | Absolute (10^3^/mm^3^) | Relative (%) | Absolute (10^3^/mm^3^) |
| Basophils | 0 | 0 |  |  |  |  |
| Eosinophils | 0 | 0 | 2 | 0.20 | 0 | 0 |
| Neutrophils (Bands) | 0 | 0 |  |  |  |  |
| Neutrophils (Segmented) | 59 | 7.26 | 73 | 7.30 | 87 | 7.60 |
| Lymphocytes | 38 | 4.67 | 23 | 2.30 | 10 | 0.87 |
| Monocytes | 3 | 0.37 | 2 | 0.20 | 3 | 261 |
| **Platelets** | 485 x 10^3^/mm^3^ | | 588 x 10^3^/mm^3^ | | 502 x 10^3^/mm^3^ | |
| **ALT** | 54 U/L  (18 a 129 U/L) | | 30.99 U/L  (18 a 129 U/L) | | 75.03 U/L  (18 a 129 U/L) | |
| **AST** | 59 U/L  (23 a 70 U/L) | | 39.87 U/L  (23 a 70 U/L) | | 71.39 U/L  (23 a 70 U/L) | |
| **Creatinine** | 0.9 mg/dL  (0.30 a 1.30 mg/dL) | | 0.98 mg/dL  (0.30 a 1.30 mg/dL) | | 1.20 mg/dL  (0.30 a 1.30 mg/dL) | |
| **GGT** | 57 U/L  (48 a 130 U/L) | | 70.72 U/L  (48 a 130 U/L) | | 44.42 U/L  (48 a 130 U/L) | |

| **AG163** | **NHPs Transfer** | | **-1d pre-challenge^#^** | | **7d post-challenge** | |
| --- | --- | --- | --- | --- | --- | --- |
| **Erythrocytes** | 4.11 millions/mm^3^ | | 5.78 millions/mm^3^ | | 4.59 millions/mm^3^ | |
| Hemoglobin | 8.20 g/dL | | 12.10 g/dL | | 10.30 g/dL | |
| Hematocrit (HCT) | 26.20 % | | 38.60 % | | 30.90 % | |
| Mean Corpuscular Volume (MCV) | 63.70 fm^3^ | | 66.78 fm^3^ | | 67.32 fm^3^ | |
| Mean Corpuscular Hemoglobin (MCH) | 20 pg | | 20.93 pg | | 22.44 pg | |
| Mean Corpuscular Hemoglobin Concentration (MCHC) | 31.30 g/dL | | 31.34 g/dL | | 33.33 g/dL | |
| **Leukocytes** | 8.50 x 10^3^/mm^3^ | | 8.80 x 10^3^/mm^3^ | | 4.39 x 10^3^/mm^3^ | |
|  | Relative (%) | Absolute (10^3^/mm^3^) | Relative (%) | Absolute (10^3^/mm^3^) | Relative (%) | Absolute (10^3^/mm^3^) |
| Basophils | 0 | 0 |  |  |  |  |
| Eosinophils | 1 | 0.08 | 0 | 0 | 3 | 0.13 |
| Neutrophils (Bands) | 3 | 0.26 |  |  |  |  |
| Neutrophils (Segmented) | 72 | 6.12 | 69 | 6.07 | 73 | 3.21 |
| Lymphocytes | 24 | 2.04 | 28 | 2.46 | 20 | 0.88 |
| Monocytes | 0 | 0 | 1 | 0.09 | 4 | 0.18 |
| **Platelets** | 411 x 10^3^/mm^3^ | | 384 x 10^3^/mm^3^ | | 367 x 10^3^/mm^3^ | |
| **ALT** | 26 U/L  (18 a 129 U/L) | | 39.66 U/L  (18 a 129 U/L) | | 81.65 U/L  (18 a 129 U/L) | |
| **AST** | 37 U/L  (23 a 70 U/L) | | 39.55 U/L  (23 a 70 U/L) | | 67.24 U/L  (23 a 70 U/L) | |
| **Creatinine** | 0.7 mg/dL  (0.30 a 1.30 mg/dL) | | 0.48 mg/dL  (0.30 a 1.30 mg/dL) | | 0.87 mg/dL  (0.30 a 1.30 mg/dL) | |
| **GGT** | 51 U/L  (48 a 130 U/L) | | 114.40 U/L  (48 a 130 U/L) | | 56.13 U/L  (48 a 130 U/L) | |

| **AG167** | **NHPs Transfer** | | **-1d pre-challenge** | | **7d post-challenge** | |
| --- | --- | --- | --- | --- | --- | --- |
| **Erythrocytes** | 5.80 millions/mm^3^ | | 6.42 millions/mm^3^ | | 5.25 millions/mm^3^ | |
| Hemoglobin | 12.70 g/dL | | 14.10 g/dL | | 11.90 g/dL | |
| Hematocrit (HCT) | 39.50 % | | 42.00 % | | 36.30 % | |
| Mean Corpuscular Volume (MCV) | 68.10 fm^3^ | | 65.42 fm^3^ | | 69.14 fm^3^ | |
| Mean Corpuscular Hemoglobin (MCH) | 21.90 pg | | 21.96 pg | | 22.66 pg | |
| Mean Corpuscular Hemoglobin Concentration (MCHC) | 32.20 g/dL | | 33.57 g/dL | | 32.78 g/dL | |
| **Leukocytes** | 6.50 x 10^3^/mm^3^ | | 7.70 x 10^3^/mm^3^ | | 4.41 x 10^3^/mm^3^ | |
|  | Relative (%) | Absolute (10^3^/mm^3^) | Relative (%) | Absolute (10^3^/mm^3^) | Relative (%) | Absolute (10^3^/mm^3^) |
| Basophils | 0 | 0 |  |  | 1 | 0.04 |
| Eosinophils | 0 | 0 | 7 | 0.54 | 1 | 0.04 |
| Neutrophils (Bands) | 1 | 0.06 |  |  |  |  |
| Neutrophils (Segmented) | 69 | 4.48 | 58 | 4.47 | 75 | 3.31 |
| Lymphocytes | 29 | 1.88 | 31 | 2.39 | 16 | 0.71 |
| Monocytes | 1 | 0.06 | 4 | 0.31 | 6 | 0.26 |
| **Platelets** | 549 x 10^3^/mm^3^ | | 383 x 10^3^/mm^3^ | | 374 x 10^3^/mm^3^ | |
| **ALT** | 37 U/L  (18 a 129 U/L) | | 25.74 U/L  (18 a 129 U/L) | | 112.50 U/L  (18 a 129 U/L) | |
| **AST** | 57 U/L  (23 a 70 U/L) | | 47.26 U/L  (23 a 70 U/L) | | 85.04 U/L  (23 a 70 U/L) | |
| **Creatinine** | 0.8 mg/dL  (0.30 a 1.30 mg/dL) | | 1.01 mg/dL  (0.30 a 1.30 mg/dL) | | 1.13 mg/dL  (0.30 a 1.30 mg/dL) | |
| **GGT** | 58 U/L  (48 a 130 U/L) | | 78.70 U/L  (48 a 130 U/L) | | 43.76 U/L  (48 a 130 U/L) | |

| **AG179** | **NHPs Transfer** | | **-1d pre-challenge** | | **7d post-challenge** | |
| --- | --- | --- | --- | --- | --- | --- |
| **Erythrocytes** | 5.81 millions/mm^3^ | | 6.30 millions/mm^3^ | | 4.91 millions/mm^3^ | |
| Hemoglobin | 11.60 g/dL | | 13.40 g/dL | | 11.20 g/dL | |
| Hematocrit (HCT) | 38.90 % | | 40.00 % | | 33.10 % | |
| Mean Corpuscular Volume (MCV) | 67 fm^3^ | | 63.49 fm^3^ | | 67.41 fm^3^ | |
| Mean Corpuscular Hemoglobin (MCH) | 20 pg | | 21.26 pg | | 22.81 pg | |
| Mean Corpuscular Hemoglobin Concentration (MCHC) | 29.80 g/dL | | 33.50 g/dL | | 33.83g/dL | |
| **Leukocytes** | 12.50 x 10^3^/mm^3^ | | 8.20 x 10^3^/mm^3^ | | 4.61 x 10^3^/mm^3^ | |
|  | Relative (%) | Absolute (10^3^/mm^3^) | Relative (%) | Absolute (10^3^/mm^3^) | Relative (%) | Absolute (10^3^/mm^3^) |
| Basophils | 0 | 0 |  |  |  |  |
| Eosinophils | 1 | 0.12 | 2 | 0.16 | 6 | 0.28 |
| Neutrophils (Bands) | 0 | 0 |  |  |  |  |
| Neutrophils (Segmented) | 75 | 9.38 | 33 | 2.71 | 55 | 2.54 |
| Lymphocytes | 20 | 2.50 | 63 | 5.166 | 37 | 1.71 |
| Monocytes | 4 | 0.50 | 1 | 0.08 | 2 | 0.09 |
| **Platelets** | 554 x 10^3^/mm^3^ | | 481 x 10^3^/mm^3^ | | 404 x 10^3^/mm^3^ | |
| **ALT** | 28 U/L  (18 a 129 U/L) | | 30.05 U/L  (18 a 129 U/L) | | 88.17 U/L  (18 a 129 U/L) | |
| **AST** | 53 U/L  (23 a 70 U/L) | | 44.14 U/L  (23 a 70 U/L) | | 82.69 U/L  (23 a 70 U/L) | |
| **Creatinine** | 0.5 mg/dL  (0.30 a 1.30 mg/dL) | | 0.44 mg/dL  (0.30 a 1.30 mg/dL) | | 0.84 mg/dL  (0.30 a 1.30 mg/dL) | |
| **GGT** | 60 U/L  (48 a 130 U/L) | | 89.19 U/L  (48 a 130 U/L) | | 39.38 U/L  (48 a 130 U/L) | |

| **AH71** | **NHPs Transfer** | | **-1d pre-challenge^#^** | | **7d post-challenge** | |
| --- | --- | --- | --- | --- | --- | --- |
| **Erythrocytes** | 5.87 millions/mm^3^ | | 6.14 millions/mm^3^ | | 5.19 millions/mm^3^ | |
| Hemoglobin | 12.90 g/dL | | 14.40 g/dL | | 12.40 g/dL | |
| Hematocrit (HCT) | 41.20 % | | 40.50 % | | 38.00 % | |
| Mean Corpuscular Volume (MCV) | 70.20 fm^3^ | | 65.96 fm^3^ | | 73.21 fm^3^ | |
| Mean Corpuscular Hemoglobin (MCH) | 22 pg | | 23.45 pg | | 23.89 pg | |
| Mean Corpuscular Hemoglobin Concentration (MCHC) | 31.30 g/dL | | 35.55 g/dL | | 32.63 g/dL | |
| **Leukocytes** | 11.20 x 10^3^/mm^3^ | | 11.90 x 10^3^/mm^3^ | | 7.46 x 10^3^/mm^3^ | |
|  | Relative (%) | Absolute (10^3^/mm^3^) | Relative (%) | Absolute (10^3^/mm^3^) | Relative (%) | Absolute (10^3^/mm^3^) |
| Basophils | 0 | 0 |  |  |  |  |
| Eosinophils | 0 | 0 | 0 | 0 | 0 | 0 |
| Neutrophils (Bands) | 0 | 0 |  |  |  |  |
| Neutrophils (Segmented) | 67 | 7.50 | 57 | 6.78 | 71 | 5.30 |
| Lymphocytes | 29 | 3.25 | 39 | 4.64 | 25 | 1.87 |
| Monocytes | 4 | 0.45 | 2 | 0.24 | 4 | 0.30 |
| **Platelets** | 598 x 10^3^/mm^3^ | | 516 x 10^3^/mm^3^ | | 367 x 10^3^/mm^3^ | |
| **ALT** | 30 U/L  (18 a 129 U/L) | | 26.69 U/L  (18 a 129 U/L) | | 62.58 U/L  (18 a 129 U/L) | |
| **AST** | 46 U/L  (23 a 70 U/L) | | 39.54 U/L  (23 a 70 U/L) | | 51.11 U/L  (23 a 70 U/L) | |
| **Creatinine** | 0.8 mg/dL  (0.30 a 1.30 mg/dL) | | 0.71 mg/dL  (0.30 a 1.30 mg/dL) | | 0.92 mg/dL  (0.30 a 1.30 mg/dL) | |
| **GGT** | 59 U/L  (48 a 130 U/L) | | 80.21 U/L  (48 a 130 U/L) | | 42.82 U/L  (48 a 130 U/L) | |

| **AG78** | **NHPs Transfer** | | **-1d pre-challenge*** | | **7d post-challenge** | |
| --- | --- | --- | --- | --- | --- | --- |
| **Erythrocytes** | 4.84 millions/mm^3^ | | 5.14 millions/mm^3^ | | 3.84 millions/mm^3^ | |
| Hemoglobin | 10 g/dL | | 11.80 g/dL | | 8.67 g/dL | |
| Hematocrit (HCT) | 32.10 % | | 35.30 % | | 26.30 % | |
| Mean Corpuscular Volume (MCV) | 66.30 fm^3^ | | 68.67 fm^3^ | | 68.48 fm^3^ | |
| Mean Corpuscular Hemoglobin (MCH) | 20.70 pg | | 22.95 pg | | 22.57 pg | |
| Mean Corpuscular Hemoglobin Concentration (MCHC) | 31.20 g/dL | | 33.42 g/dL | | 32.96 g/dL | |
| **Leukocytes** | 5.70 x 10^3^/mm^3^ | | 10.40 x 10^3^/mm^3^ | | 3.05 x 10^3^/mm^3^ | |
|  | Relative (%) | Absolute (10^3^/mm^3^) | Relative (%) | Absolute (10^3^/mm^3^) | Relative (%) | Absolute (10^3^/mm^3^) |
| Basophils | 0 | 0 |  |  |  |  |
| Eosinophils | 1 | 0.06 | 0 | 0 | 0 | 0 |
| Neutrophils (Bands) | 2 | 0.11 |  |  |  |  |
| Neutrophils (Segmented) | 41 | 2.34 | 77 | 8.01 | 75 | 2.29 |
| Lymphocytes | 50 | 2.85 | 16 | 1.66 | 16 | 0.49 |
| Monocytes | 6 | 0.34 | 7 | 0.73 | 9 | 0.27 |
| **Platelets** | 557 x 10^3^/mm^3^ | | 510 x 10^3^/mm^3^ | | 359 x 10^3^/mm^3^ | |
| **ALT** | 32 U/L  (18 a 129 U/L) | | 41.76 U/L  (18 a 129 U/L) | | 73.30 U/L  (18 a 129 U/L) | |
| **AST** | 36 U/L  (23 a 70 U/L) | | 36.41 U/L  (23 a 70 U/L) | | 88.92 U/L  (23 a 70 U/L) | |
| **Creatinine** | 0.5 mg/dL  (0.30 a 1.30 mg/dL) | | 0.49 mg/dL  (0.30 a 1.30 mg/dL) | | 0.65 mg/dL  (0.30 a 1.30 mg/dL) | |
| **GGT** | 58 U/L  (48 a 130 U/L) | | 81.45 U/L  (48 a 130 U/L) | | 67.64 U/L  (48 a 130 U/L) | |

| **AH85** | **NHPs Transfer** | | **-1d pre-challenge^#^** | | **7d post-challenge** | |
| --- | --- | --- | --- | --- | --- | --- |
| **Erythrocytes** | 5.51 millions/mm^3^ | | 6.60 millions/mm^3^ | | 4.86 millions/mm^3^ | |
| Hemoglobin | 12.30 g/dL | | 14.70 g/dL | | 11.30 g/dL | |
| Hematocrit (HCT) | 38.60 % | | 42.00 % | | 34.60 % | |
| Mean Corpuscular Volume (MCV) | 70.10 fm^3^ | | 63.63 fm^3^ | | 71.19 fm^3^ | |
| Mean Corpuscular Hemoglobin (MCH) | 22.30 pg | | 22.27 pg | | 23.25 pg | |
| Mean Corpuscular Hemoglobin Concentration (MCHC) | 31.90 g/dL | | 35.00 g/dL | | 32.65 g/dL | |
| **Leukocytes** | 10 x 10^3^/mm^3^ | | 11.80 x 10^3^/mm^3^ | | 22.80 x 10^3^/mm^3^ | |
|  | Relative (%) | Absolute (10^3^/mm^3^) | Relative (%) | Absolute (10^3^/mm^3^) | Relative (%) | Absolute (10^3^/mm^3^) |
| Basophils | 0 | 0 |  |  | 2 | 0.46 |
| Eosinophils | 1 | 0.10 | 0 | 0 | 3 | 0.68 |
| Neutrophils (Bands) | 1 | 0.10 |  |  |  |  |
| Neutrophils (Segmented) | 73 | 7.30 | 67 | 7.91 | 49 | 11.17 |
| Lymphocytes | 24 | 2.40 | 25 | 2.95 | 39 | 8.89 |
| Monocytes | 1 | 0.10 | 8 | 0.94 | 7 | 1.60 |
| **Platelets** | 381 x 10^3^/mm^3^ | | 427 x 10^3^/mm^3^ | | 389 x 10^3^/mm^3^ | |
| **ALT** | 28 U/L  (18 a 129 U/L) | | 35.94 U/L  (18 a 129 U/L) | | 76.06 U/L  (18 a 129 U/L) | |
| **AST** | 48 U/L  (23 a 70 U/L) | | 39.89 U/L  (23 a 70 U/L) | | 57.73 U/L  (23 a 70 U/L) | |
| **Creatinine** | 0.7 mg/dL  (0.30 a 1.30 mg/dL) | | 0.54 mg/dL  (0.30 a 1.30 mg/dL) | | 0.78 mg/dL  (0.30 a 1.30 mg/dL) | |
| **GGT** | 62 U/L  (48 a 130 U/L) | | 105.10 U/L  (48 a 130 U/L) | | 50.30 U/L  (48 a 130 U/L) | |

| **AG110†** | **NHPs Transfer** | | **-1d pre-challenge** | | **7d post-challenge** | |
| --- | --- | --- | --- | --- | --- | --- |
| **Erythrocytes** | 5.07 millions/mm^3^ | |  | |  | |
| Hemoglobin | 11.30 g/dL | |  | |  | |
| Hematocrit (HCT) | 36 % | |  | |  | |
| Mean Corpuscular Volume (MCV) | 71 fm^3^ | |  | |  | |
| Mean Corpuscular Hemoglobin (MCH) | 22.30 pg | |  | |  | |
| Mean Corpuscular Hemoglobin Concentration (MCHC) | 31.40 g/dL | |  | |  | |
| **Leukocytes** | 2.90 x 10^3^/mm^3^ | |  | |  | |
|  | Relative (%) | Absolute (10^3^/mm^3^) | Relative (%) | Absolute (10^3^/mm^3^) | Relative (%) | Absolute (10^3^/mm^3^) |
| Basophils | 0 | 0 |  |  |  |  |
| Eosinophils | 1 | 0.03 |  |  |  |  |
| Neutrophils (Bands) | 0 | 0 |  |  |  |  |
| Neutrophils (Segmented) | 49 | 1.42 |  |  |  |  |
| Lymphocytes | 50 | 1.45 |  |  |  |  |
| Monocytes | 0 | 0 |  |  |  |  |
| **Platelets** | 643 x 10^3^/mm^3^ | |  | |  | |
| **ALT** | 34 U/L  (18 a 129 U/L) | |  | |  | |
| **AST** | 45 U/L  (23 a 70 U/L) | |  | |  | |
| **Creatinine** | 0.6 mg/dL  (0.30 a 1.30 mg/dL) | |  | |  | |
| **GGT** | 42 U/L  (48 a 130 U/L) | |  | |  | |

**Group 4**

| **AH57** | **NHPs Transfer*** | | **-1d pre-challenge*** | | **7d post-challenge** | |
| --- | --- | --- | --- | --- | --- | --- |
| **Erythrocytes** | 5.64 millions/mm^3^ | | 5.42 millions/mm^3^ | | 4.83 millions/mm^3^ | |
| Hemoglobin | 12.8 g/dL | | 12.40 g/dL | | 11.20 g/dL | |
| Globular Volume |  | |  | |  | |
| Hematocrit (HCT) | 41.7 % | | 40.70 % | | 36.60 % | |
| Mean Corpuscular Volume (MCV) | 73.9 fm^3^ | | 75.10 fm^3^ | | 75.80 fm^3^ | |
| Mean Corpuscular Hemoglobin (MCH) | 22.7 pg | | 22.90 pg | | 23.20 pg | |
| Mean Corpuscular Hemoglobin Concentration (MCHC) | 30.7 g/dL | | 30.50 g/dL | | 30.60 g/dL | |
| Note:  *****Normocytic and normochromic erythrocytes. | | | | | | |
| **Leukocytes** | 11.4 x 10^3^/mm^3^ | | 9.60 x 10^3^/mm^3^ | | 5.50 x 10^3^/mm^3^ | |
|  | Relative (%) | Absolute (10^3^/mm^3^) | Relative (%) | Absolute (10^3^/mm^3^) | Relative (%) | Absolute (10^3^/mm^3^) |
| Basophils | 0 |  | 0 | 0 | 0 | 0 |
| Eosinophils | 0 |  | 1 | 0.01 | 0 | 0 |
| Myelocytes |  |  |  |  |  |  |
| Metamyelocytes |  |  |  |  |  |  |
| Neutrophils (Bands) | 0 |  | 0 | 0 | 0 | 0 |
| Neutrophils (Segmented) | 57 |  | 39 | 0.39 | 32 | 1.76 |
| Lymphocytes | 42 |  | 55 | 0.55 | 63 | 3.46 |
| Monocytes | 1 |  | 5 | 0.05 | 5 | 0.28 |
| **Platelets** | 373 x 10^3^/mm^3^ | | 363 x 10^3^/mm^3^ | | 350 x 10^3^/mm^3^ | |
| **ALT** | 32 U/L  (18 a 129 U/L) | | 36 U/L  (18 a 129 U/L) | | 78 U/L  (18 a 129 U/L) | |
| **AST** | 49 U/L  (23 a 70 U/L) | | 64 U/L  (23 a 70 U/L) | | 93 U/L  (23 a 70 U/L) | |
| **Creatinine** | 0.8 mg/dL  (0.30 a 1.30 mg/dL) | | 0.8 mg/dL  (0.30 a 1.30 mg/dL) | | 0.7 mg/dL  (0.30 a 1.30 mg/dL) | |
| **GGT** | 63 U/L  (48 a 130 U/L) | | 64 U/L  (48 a 130 U/L) | | 61 U/L  (48 a 130 U/L) | |

| **AH63** | **NHPs Transfer*** | | **-1d pre-challenge*** | | **7d post-challenge** | |
| --- | --- | --- | --- | --- | --- | --- |
| **Erythrocytes** | 4.78 millions/mm^3^ | | 5.72 millions/mm^3^ | | 5.03 millions/mm^3^ | |
| Hemoglobin | 10.3 g/dL | | 12.50 g/dL | | 11.20 g/dL | |
| Globular Volume |  | |  | |  | |
| Hematocrit (HCT) | 34.9 % | | 42.60 % | | 37.40 % | |
| Mean Corpuscular Volume (MCV) | 73.0 fm^3^ | | 74.50 fm^3^ | | 74.40 fm^3^ | |
| Mean Corpuscular Hemoglobin (MCH) | 21.5 pg | | 21.90 pg | | 22.30 pg | |
| Mean Corpuscular Hemoglobin Concentration (MCHC) | 29.5 g/dL | | 29.30 g/dL | | 29.90 g/dL | |
| Note:  *****Normocytic and normochromic erythrocytes. | | | | | | |
| **Leukocytes** | 13.6 x 10^3^/mm^3^ | | 7.90 x 10^3^/mm^3^ | | 3.30 x 10^3^/mm^3^ | |
|  | Relative (%) | Absolute (10^3^/mm^3^) | Relative (%) | Absolute (10^3^/mm^3^) | Relative (%) | Absolute (10^3^/mm^3^) |
| Basophils | 0 |  | 0 | 0 | 1 | 0.03 |
| Eosinophils | 1 |  | 0 | 0 | 0 | 0 |
| Myelocytes |  |  |  |  |  |  |
| Metamyelocytes |  |  |  |  |  |  |
| Neutrophils (Bands) | 0 |  | 0 | 0 | 1 | 0.03 |
| Neutrophils (Segmented) | 76 |  | 49 | 0.49 | 38 | 1.25 |
| Lymphocytes | 21 |  | 51 | 0.51 | 50 | 1.65 |
| Monocytes | 2 |  | 0 | 0 | 10 | 0.33 |
| **Platelets** | 644 x 10^3^/mm^3^ | | 396 x 10^3^/mm^3^ | | 355 x 10^3^/mm^3^ | |
| **ALT** | 18 U/L  (18 a 129 U/L) | | 16 U/L  (18 a 129 U/L) | | 37 U/L  (18 a 129 U/L) | |
| **AST** | 44 U/L  (23 a 70 U/L) | | 47 U/L  (23 a 70 U/L) | | 79 U/L  (23 a 70 U/L) | |
| **Creatinine** | 0.7 mg/dL  (0.30 a 1.30 mg/dL) | | 0.7 mg/dL  (0.30 a 1.30 mg/dL) | | 0.6 mg/dL  (0.30 a 1.30 mg/dL) | |
| **GGT** | 57 U/L  (48 a 130 U/L) | | 68 U/L  (48 a 130 U/L) | | 63 U/L  (48 a 130 U/L) | |

| **AF92** | **NHPs Transfer*** | | **-1d pre-challenge^#^** | | **7d post-challenge** | |
| --- | --- | --- | --- | --- | --- | --- |
| **Erythrocytes** |  | | 4.88 millions/mm^3^ | | 4.05 millions/mm^3^ | |
| Hemoglobin |  | | 10.50 g/dL | | 8.60 g/dL | |
| Globular Volume |  | |  | |  | |
| Hematocrit (HCT) |  | | 35.30 % | | 29.10 % | |
| Mean Corpuscular Volume (MCV) |  | | 72.30 fm^3^ | | 71.90 fm^3^ | |
| Mean Corpuscular Hemoglobin (MCH) |  | | 21.50 pg | | 21.20 pg | |
| Mean Corpuscular Hemoglobin Concentration (MCHC) |  | | 29.70 g/dL | | 29.60 g/dL | |
| Note:  **^#^**Normocytic and normochromic erythrocytes. | | | | | | |
| **Leukocytes** |  | | 10.90 x 10^3^/mm^3^ | | 8 x 10^3^/mm^3^ | |
|  | Relative (%) | Absolute (10^3^/mm^3^) | Relative (%) | Absolute (10^3^/mm^3^) | Relative (%) | Absolute (10^3^/mm^3^) |
| Basophils |  |  | 0 | 0 | 0 | 0 |
| Eosinophils |  |  | 0 | 0 | 0 | 0 |
| Myelocytes |  |  |  |  |  |  |
| Metamyelocytes |  |  |  |  |  |  |
| Neutrophils (Bands) |  |  | 0 | 0 | 0 | 0 |
| Neutrophils (Segmented) |  |  | 64 | 0.64 | 61 | 4.88 |
| Lymphocytes |  |  | 34 | 0.34 | 37 | 2.96 |
| Monocytes |  |  | 2 | 0.02 | 2 | 0.16 |
| **Platelets** |  | | 258 x 10^3^/mm^3^ | | 351 x 10^3^/mm^3^ | |
| Note:  *****Hematoly results not sent by laboratory. | | | | | | |
| **ALT** | 27 U/L  (18 a 129 U/L) | | 15 U/L  (18 a 129 U/L) | | 39 U/L  (18 a 129 U/L) | |
| **AST** | 41 U/L  (23 a 70 U/L) | | 36 U/L  (23 a 70 U/L) | | 43 U/L  (23 a 70 U/L) | |
| **Creatinine** | 0.7 mg/dL  (0.30 a 1.30 mg/dL) | | 0.6 mg/dL  (0.30 a 1.30 mg/dL) | | 0.6 mg/dL  (0.30 a 1.30 mg/dL) | |
| **GGT** | 67 U/L  (48 a 130 U/L) | | 70 U/L  (48 a 130 U/L) | | 61 U/L  (48 a 130 U/L) | |

| **AG48** | **NHPs Transfer*** | | **-1d pre-challenge** | | **7d post-challenge** | |
| --- | --- | --- | --- | --- | --- | --- |
| **Erythrocytes** | 5.77 millions/mm3 | | 5.16 millions/mm^3^ | | 4.27 millions/mm^3^ | |
| Hemoglobin | 12.70 g/dL | | 11.30 g/dL | | 9.20 g/dL | |
| Globular Volume |  | |  | |  | |
| Hematocrit (HCT) | 42.20 % | | 38.10 % | | 31.10 % | |
| Mean Corpuscular Volume (MCV) | 73.10 fm3 | | 73.80 fm^3^ | | 72.80 fm^3^ | |
| Mean Corpuscular Hemoglobin (MCH) | 22 pg | | 21.90 pg | | 21.50 pg | |
| Mean Corpuscular Hemoglobin Concentration (MCHC) | 30.10 g/dL | | 29.70 g/dL | | 29.60 g/dL | |
| Note:  *****Normocytic and normochromic erythrocytes. | | | | | | |
| **Leukocytes** | 6.90 x 10^3^/mm^3^ | | 7.90 x 10^3^/mm^3^ | | 5.50 x 10^3^/mm^3^ | |
|  | Relative (%) | Absolute (10^3^/mm^3^) | Relative (%) | Absolute (10^3^/mm^3^) | Relative (%) | Absolute (10^3^/mm^3^) |
| Basophils | 0 | 0 | 0 | 0 | 0 | 0 |
| Eosinophils | 0 | 0 | 0 | 0 | 2 | 0.11 |
| Myelocytes |  |  |  |  |  |  |
| Metamyelocytes |  |  |  |  |  |  |
| Neutrophils (Bands) | 0 | 0 | 0 | 0 | 1 | 0.06 |
| Neutrophils (Segmented) | 30 | 0.3 | 48 | 3.79 | 56 | 3.08 |
| Lymphocytes | 68 | 4.69 | 49 | 3.87 | 36 | 1.98 |
| Monocytes | 2 | 0.14 | 3 | 0.24 | 5 | 0.28 |
| **Platelets** | 409 x 10^3^/mm^3^ | | 463 x 10^3^/mm^3^ | | 374 x 10^3^/mm^3^ | |
| **ALT** | 24 U/L  (18 a 129 U/L) | | 27 U/L  (18 a 129 U/L) | | 24 U/L  (18 a 129 U/L) | |
| **AST** | 35 U/L  (23 a 70 U/L) | | 59 U/L  (23 a 70 U/L) | | 47 U/L  (23 a 70 U/L) | |
| **Creatinine** | 0.6 mg/dL  (0.30 a 1.30 mg/dL) | | 0.7 mg/dL  (0.30 a 1.30 mg/dL) | | 0.7 mg/dL  (0.30 a 1.30 mg/dL) | |
| **GGT** | 51 U/L  (48 a 130 U/L) | | 50 U/L  (48 a 130 U/L) | | 47 U/L  (48 a 130 U/L) | |

| **AG57** | **NHPs Transfer*** | | **-1d pre-challenge*** | | **7d post-challenge** | |
| --- | --- | --- | --- | --- | --- | --- |
| **Erythrocytes** | 5.86 millions/mm^3^ | | 5.46 millions/mm^3^ | | 4.84 millions/mm^3^ | |
| Hemoglobin | 11.10 g/dL | | 10.60 g/dL | | 9.40 g/dL | |
| Globular Volume |  | |  | |  | |
| Hematocrit (HCT) | 37.90 % | | 35.10 % | | 31.20 % | |
| Mean Corpuscular Volume (MCV) | 64.70 fm^3^ | | 64.30 fm^3^ | | 64.50 fm^3^ | |
| Mean Corpuscular Hemoglobin (MCH) | 18.90 pg | | 19.40 pg | | 19.40 pg | |
| Mean Corpuscular Hemoglobin Concentration (MCHC) | 29.30 g/dL | | 30.20 g/dL | | 30.10 g/dL | |
| Note:  *****Normocytic and normochromic erythrocytes. | | | | | | |
| **Leukocytes** | 12.60 x 10^3^/mm^3^ | | 13.40 x 10^3^/mm^3^ | | 8 x 10^3^/mm^3^ | |
|  | Relative (%) | Absolute (10^3^/mm^3^) | Relative (%) | Absolute (10^3^/mm^3^) | Relative (%) | Absolute (10^3^/mm^3^) |
| Basophils | 0 | 0 | 0 | 0 | 0 | 0 |
| Eosinophils | 1 | 0.13 | 1 | 0.13 | 2 | 0.16 |
| Myelocytes |  |  |  |  |  |  |
| Metamyelocytes |  |  |  |  |  |  |
| Neutrophils (Bands) | 0 | 0 | 1 | 0.13 | 2 | 0.16 |
| Neutrophils (Segmented) | 39 | 0.39 | 29 | 3.89 | 24 | 1.92 |
| Lymphocytes | 52 | 6.55 | 67 | 8.98 | 67 | 5.36 |
| Monocytes | 8 | 1.01 | 2 | 0.27 | 5 | 0.40 |
| **Platelets** | 326 x 10^3^/mm^3^ | | 515 x 10^3^/mm^3^ | | 491 x 10^3^/mm^3^ | |
| **ALT** | 13 U/L  (18 a 129 U/L) | | 10 U/L  (18 a 129 U/L) | | 67 U/L  (18 a 129 U/L) | |
| **AST** | 35 U/L  (23 a 70 U/L) | | 36 U/L  (23 a 70 U/L) | | 91 U/L  (23 a 70 U/L) | |
| **Creatinine** | 0.7 mg/dL  (0.30 a 1.30 mg/dL) | | 0.6 mg/dL  (0.30 a 1.30 mg/dL) | | 0.8 mg/dL  (0.30 a 1.30 mg/dL) | |
| **GGT** | 46 U/L  (48 a 130 U/L) | | 46 U/L  (48 a 130 U/L) | | 67 U/L  (48 a 130 U/L) | |

| **AG71** | **NHPs Transfer*** | | **-1d pre-challenge*** | | **7d post-challenge** | |
| --- | --- | --- | --- | --- | --- | --- |
| **Erythrocytes** | 6.26 millions/mm^3^ | | 5.56 millions/mm^3^ | | 4.83 millions/mm^3^ | |
| Hemoglobin | 13.40 g/dL | | 11.60 g/dL | | 10.20 g/dL | |
| Globular Volume |  | |  | |  | |
| Hematocrit (HCT) | 43.60 % | | 38.10 % | | 33.30 % | |
| Mean Corpuscular Volume (MCV) | 69.60 fm^3^ | | 68.50 fm^3^ | | 68.90 fm^3^ | |
| Mean Corpuscular Hemoglobin (MCH) | 21.40 pg | | 20.90 pg | | 21.10 pg | |
| Mean Corpuscular Hemoglobin Concentration (MCHC) | 30.70 g/dL | | 30.40 g/dL | | 30.60 g/dL | |
| Note:  *Normocytic and normochromic erythrocytes. | | | | | | |
| **Leukocytes** | 5.20 x 10^3^/mm^3^ | | 5.50 x 10^3^/mm^3^ | | 4.10 x 10^3^/mm^3^ | |
|  | Relative (%) | Absolute (10^3^/mm^3^) | Relative (%) | Absolute (10^3^/mm^3^) | Relative (%) | Absolute (10^3^/mm^3^) |
| Basophils | 0 | 0 | 0 | 0 | 0 | 0 |
| Eosinophils | 1 | 0.05 | 0 | 0 | 0 | 0 |
| Myelocytes |  |  |  |  |  |  |
| Metamyelocytes |  |  |  |  |  |  |
| Neutrophils (Bands) | 0 | 0 | 0 | 0 | 2 | 0.08 |
| Neutrophils (Segmented) | 51 | 0.51 | 36 | 1.98 | 53 | 2.17 |
| Lymphocytes | 43 | 2.24 | 60 | 3.30 | 39 | 1.60 |
| Monocytes | 5 | 0.26 | 4 | 0.22 | 6 | 0.25 |
| **Platelets** | 378 x 10^3^/mm^3^ | | 344 x 10^3^/mm^3^ | | 368 x 10^3^/mm^3^ | |
| **ALT** | 42 U/L  (18 a 129 U/L) | | 19 U/L  (18 a 129 U/L) | | 32 U/L  (18 a 129 U/L) | |
| **AST** | 42 U/L  (23 a 70 U/L) | | 52 U/L  (23 a 70 U/L) | | 66 U/L  (23 a 70 U/L) | |
| **Creatinine** | 1.0 mg/dL  (0.30 a 1.30 mg/dL) | | 0.9 mg/dL  (0.30 a 1.30 mg/dL) | | 0.6 mg/dL  (0.30 a 1.30 mg/dL) | |
| **GGT** | 61 U/L  (48 a 130 U/L) | | 71 U/L  (48 a 130 U/L) | | 95 U/L  (48 a 130 U/L) | |

| **AG105** | **NHPs Transfer*** | | **-1d pre-challenge*** | | **7d post-challenge** | |
| --- | --- | --- | --- | --- | --- | --- |
| **Erythrocytes** | 5.48 millions/mm^3^ | | 4.73 millions/mm^3^ | | 4.48 millions/mm^3^ | |
| Hemoglobin | 12.60 g/dL | | 10.60 g/dL | | 10 g/dL | |
| Globular Volume |  | |  | |  | |
| Hematocrit (HCT) | 41.50 % | | 36.30 % | | 33.50 % | |
| Mean Corpuscular Volume (MCV) | 75.70 fm^3^ | | 76.70 fm^3^ | | 74.80 fm^3^ | |
| Mean Corpuscular Hemoglobin (MCH) | 23 pg | | 22.40 pg | | 22.30 pg | |
| Mean Corpuscular Hemoglobin Concentration (MCHC) | 30.40 g/dL | | 29.20 g/dL | | 29.90 g/dL | |
| Note:  *****Normocytic and normochromic erythrocytes. | | | | | | |
| **Leukocytes** | 12.40 x 10^3^/mm^3^ | | 9.70 x 10^3^/mm^3^ | | 4.70 x 10^3^/mm^3^ | |
|  | Relative (%) | Absolute (10^3^/mm^3^) | Relative (%) | Absolute (10^3^/mm^3^) | Relative (%) | Absolute (10^3^/mm^3^) |
| Basophils | 0 | 0 | 0 | 0 | 0 | 0 |
| Eosinophils | 2 | 0.25 | 1 | 0.01 | 0 | 0 |
| Myelocytes |  |  |  |  |  |  |
| Metamyelocytes |  |  |  |  |  |  |
| Neutrophils (Bands) | 1 | 0.12 | 0 | 0 | 1 | 0.05 |
| Neutrophils (Segmented) | 72 | 8.93 | 57 | 0.57 | 67 | 3.15 |
| Lymphocytes | 20 | 0.20 | 41 | 0.41 | 27 | 1.27 |
| Monocytes | 5 | 0.05 | 1 | 0.01 | 5 | 0.24 |
| **Platelets** | 646 x 10^3^/mm^3^ | | 548 x 10^3^/mm^3^ | | 663 x 10^3^/mm^3^ | |
| **ALT** | 23 U/L  (18 a 129 U/L) | | 18 U/L  (18 a 129 U/L) | | 69 U/L  (18 a 129 U/L) | |
| **AST** | 45 U/L  (23 a 70 U/L) | | 58 U/L  (23 a 70 U/L) | | 68 U/L  (23 a 70 U/L) | |
| **Creatinine** | 0.7 mg/dL  (0.30 a 1.30 mg/dL) | | 0.7 mg/dL  (0.30 a 1.30 mg/dL) | | 0.7 mg/dL  (0.30 a 1.30 mg/dL) | |
| **GGT** | 56 U/L  (48 a 130 U/L) | | 60 U/L  (48 a 130 U/L) | | 84 U/L  (48 a 130 U/L) | |

| **AG148** | **NHPs Transfer** | | **-1d pre-challenge*** | | **7d post-challenge** | |
| --- | --- | --- | --- | --- | --- | --- |
| **Erythrocytes** | 4.74 millions/mm^3^ | | 5.20 millions/mm^3^ | | 4.39 millions/mm^3^ | |
| Hemoglobin | 10.60 g/dL | | 11.60 g/dL | | 9.70 g/dL | |
| Globular Volume |  | |  | |  | |
| Hematocrit (HCT) | 36 % | | 39.20 % | | 33.10 % | |
| Mean Corpuscular Volume (MCV) | 75.90 fm^3^ | | 75.40 fm^3^ | | 75.40 fm^3^ | |
| Mean Corpuscular Hemoglobin (MCH) | 22.40 pg | | 22.30 pg | | 22.10 pg | |
| Mean Corpuscular Hemoglobin Concentration (MCHC) | 29.40 g/dL | | 29.60 g/dL | | 29.30 g/dL | |
| Note:  *****Normocytic and normochromic erythrocytes. | | | | | | |
| **Leukocytes** | 8.50 x 10^3^/mm^3^ | | 8.90 x 10^3^/mm^3^ | | 2.80 x 10^3^/mm^3^ | |
|  | Relative (%) | Absolute (10^3^/mm^3^) | Relative (%) | Absolute (10^3^/mm^3^) | Relative (%) | Absolute (10^3^/mm^3^) |
| Basophils | 0 | 0 | 0 | 0 | 0 | 0 |
| Eosinophils | 0 | 0 | 0 | 0 | 1 | 0.03 |
| Myelocytes |  |  |  |  |  |  |
| Metamyelocytes |  |  |  |  |  |  |
| Neutrophils (Bands) | 0 | 0 | 0 | 0 | 1 | 0.03 |
| Neutrophils (Segmented) | 45 | 3.82 | 37 | 0.37 | 56 | 1.57 |
| Lymphocytes | 50 | 4.25 | 58 | 0.58 | 37 | 1.04 |
| Monocytes | 5 | 0.42 | 5 | 0.05 | 4 | 0.11 |
| **Platelets** | 324 x 10^3^/mm^3^ | | 475 x 10^3^/mm^3^ | | 276 x 10^3^/mm^3^ | |
| **ALT** | 22 U/L  (18 a 129 U/L) | | 14 U/L  (18 a 129 U/L) | | 54 U/L  (18 a 129 U/L) | |
| **AST** | 34 U/L  (23 a 70 U/L) | | 42 U/L  (23 a 70 U/L) | | 69 U/L  (23 a 70 U/L) | |
| **Creatinine** | 0.4 mg/dL  (0.30 a 1.30 mg/dL) | | 0.5 mg/dL  (0.30 a 1.30 mg/dL) | | 0.8 mg/dL  (0.30 a 1.30 mg/dL) | |
| **GGT** | 46 U/L  (48 a 130 U/L) | | 48U/L  (48 a 130 U/L) | | 85 U/L  (48 a 130 U/L) | |

| **AH43** | **NHPs Transfer** | | **-1d pre-challenge*** | | **7d post-challenge** | |
| --- | --- | --- | --- | --- | --- | --- |
| **Erythrocytes** | 7.70 millions/mm^3^ | | 5.26 millions/mm^3^ | | 5.14 millions/mm^3^ | |
| Hemoglobin | 6.01 g/dL | | 11.40 g/dL | | 11.30 g/dL | |
| Globular Volume |  | |  | |  | |
| Hematocrit (HCT) | 13.30 % | | 36.80 % | | 36.30 % | |
| Mean Corpuscular Volume (MCV) | 41.70 fm^3^ | | 70 fm^3^ | | 70.60 fm^3^ | |
| Mean Corpuscular Hemoglobin (MCH) | 69.40 pg | | 21.70 pg | | 22 pg | |
| Mean Corpuscular Hemoglobin Concentration (MCHC) | 22.10 g/dL | | 31 g/dL | | 31.10 g/dL | |
| Note:  *****Normocytic and normochromic erythrocytes. | | | | | | |
| **Leukocytes** | 31.90 x 10^3^/mm^3^ | | 8.20 x 10^3^/mm^3^ | | 5.70 x 10^3^/mm^3^ | |
|  | Relative (%) | Absolute (10^3^/mm^3^) | Relative (%) | Absolute (10^3^/mm^3^) | Relative (%) | Absolute (10^3^/mm^3^) |
| Basophils | 0 | 0 | 0 | 0 | 1 | 0.06 |
| Eosinophils | 0 | 0 | 0 | 0 | 0 | 0 |
| Myelocytes |  |  |  |  |  |  |
| Metamyelocytes |  |  |  |  |  |  |
| Neutrophils (Bands) | 0 | 0 | 0 | 0 | 0 | 0 |
| Neutrophils (Segmented) | 52 | 16.59 | 44 | 0.44 | 57 | 3.25 |
| Lymphocytes | 47 | 14.99 | 51 | 0.51 | 40 | 2.28 |
| Monocytes | 1 | 0.32 | 5 | 0.05 | 2 | 0.11 |
| **Platelets** | 448 x 10^3^/mm^3^ | | 567 x 10^3^/mm^3^ | | 424 x 10^3^/mm^3^ | |
| **ALT** | 47 U/L  (18 a 129 U/L) | | 41 U/L  (18 a 129 U/L) | | 61 U/L  (18 a 129 U/L) | |
| **AST** | 44 U/L  (23 a 70 U/L) | | 56 U/L  (23 a 70 U/L) | | 87 U/L  (23 a 70 U/L) | |
| **Creatinine** | 0.7 mg/dL  (0.30 a 1.30 mg/dL) | | 0.6 mg/dL  (0.30 a 1.30 mg/dL) | | 0.8 mg/dL  (0.30 a 1.30 mg/dL) | |
| **GGT** | 68 U/L  (48 a 130 U/L) | | 67 U/L  (48 a 130 U/L) | | 70 U/L  (48 a 130 U/L) | |

| **AH73** | **NHPs Transfer*** | | **-1d pre-challenge*** | | **7d post-challenge** | |
| --- | --- | --- | --- | --- | --- | --- |
| **Erythrocytes** | 6.21 millions/mm^3^ | | 5.76 millions/mm^3^ | | 4.75 millions/mm^3^ | |
| Hemoglobin | 13.30 g/dL | | 12.30 g/dL | | 10.30 g/dL | |
| Globular Volume |  | |  | |  | |
| Hematocrit (HCT) | 43.90 % | | 41 % | | 33.90 % | |
| Mean Corpuscular Volume (MCV) | 70.70 fm^3^ | | 71.20 fm^3^ | | 71.40 fm^3^ | |
| Mean Corpuscular Hemoglobin (MCH) | 21.40 pg | | 21.40 pg | | 21.70 pg | |
| Mean Corpuscular Hemoglobin Concentration (MCHC) | 30.30 g/dL | | 30 g/dL | | 30.40 g/dL | |
| Note:  *****Normocytic and normochromic erythrocytes. | | | | | | |
| **Leukocytes** | 8.90 x 10^3^/mm^3^ | | 9.70 x 10^3^/mm^3^ | | 6.30 x 10^3^/mm^3^ | |
|  | Relative (%) | Absolute (10^3^/mm^3^) | Relative (%) | Absolute (10^3^/mm^3^) | Relative (%) | Absolute (10^3^/mm^3^) |
| Basophils | 0 | 0 | 0 | 0 | 0 | 0 |
| Eosinophils | 0 | 0 | 0 | 0 | 0 | 0 |
| Myelocytes |  |  |  |  |  |  |
| Metamyelocytes |  |  |  |  |  |  |
| Neutrophils (Bands) | 0 | 0 | 0 | 0 | 3 | 0.19 |
| Neutrophils (Segmented) | 56 | 0.56 | 58 | 0.58 | 40 | 2.52 |
| Lymphocytes | 43 | 3.83 | 42 | 0.42 | 56 | 3.53 |
| Monocytes | 1 | 0.09 | 0 | 0 | 1 | 0.06 |
| **Platelets** | 405 x 10^3^/mm^3^ | | 256 x 10^3^/mm^3^ | | 318 x 10^3^/mm^3^ | |
| **ALT** | 31 U/L  (18 a 129 U/L) | | 22 U/L  (18 a 129 U/L) | | 55 U/L  (18 a 129 U/L) | |
| **AST** | 32 U/L  (23 a 70 U/L) | | 45 U/L  (23 a 70 U/L) | | 76 U/L  (23 a 70 U/L) | |
| **Creatinine** | 0.0 mg/dL  (0.30 a 1.30 mg/dL) | | 0.7 mg/dL  (0.30 a 1.30 mg/dL) | | 0.8 mg/dL  (0.30 a 1.30 mg/dL) | |
| **GGT** | 90 U/L  (48 a 130 U/L) | | 0.0 U/L  (48 a 130 U/L) | | 72 U/L  (48 a 130 U/L) | |

**Group 5**

| **AG36** | **-1d pre-challenge*** | | **7d post-challenge** | |
| --- | --- | --- | --- | --- |
| **Erythrocytes** | 5.07 millions/mm^3^ | | 4.19 millions/mm^3^ | |
| Hemoglobin | 10.80 g/dL | | 9.58 g/dL | |
| Hematocrit (HCT) | 34.20 % | | 29.90 % | |
| Mean Corpuscular Volume (MCV) | 67.45 fm^3^ | | 71.36 fm^3^ | |
| Mean Corpuscular Hemoglobin (MCH) | 21.30 pg | | 22.86 pg | |
| Mean Corpuscular Hemoglobin Concentration (MCHC) | 31.57 g/dL | | 32.04 g/dL | |
| **Leukocytes** | 9.90 x 10^3^/mm^3^ | | 6.46 x 10^3^/mm^3^ | |
|  | Relative (%) | Absolute (10^3^/mm^3^) | Relative (%) | Absolute (10^3^/mm^3^) |
| Basophils |  |  |  |  |
| Eosinophils | 1 | 0.01 | 0 | 0 |
| Neutrophils (Bands) |  |  |  |  |
| Neutrophils (Segmented) | 72 | 7.13 | 71 | 4.59 |
| Lymphocytes | 19 | 1.88 | 22 | 1.42 |
| Monocytes | 8 | 0.79 | 7 | 0.45 |
| **Platelets** | 677 x 10^3^/mm^3^ | | 393 x 10^3^/mm^3^ | |
| **ALT** | 24.54 U/L  (18 a 129 U/L) | | 68.12 U/L  (18 a 129 U/L) | |
| **AST** | 28.92 U/L  (23 a 70 U/L) | | 43.66 U/L  (23 a 70 U/L) | |
| **Creatinine** | 0.53 mg/dL  (0.30 a 1.30 mg/dL) | | 0.73 mg/dL  (0.30 a 1.30 mg/dL) | |
| **GGT** | 39.47 U/L  (48 a 130 U/L) | | 33.86 U/L  (48 a 130 U/L) | |

| **AE6** | **-1d pre-challenge** | | **7d post-challenge** | |
| --- | --- | --- | --- | --- |
| **Erythrocytes** | 6.06 millions/mm^3^ | | 5.24 millions/mm^3^ | |
| Hemoglobin | 12.70 g/dL | | 11.60 g/dL | |
| Hematocrit (HCT) | 40.20 % | | 35.50 % | |
| Mean Corpuscular Volume (MCV) | 66.33 fm^3^ | | 67.74 fm^3^ | |
| Mean Corpuscular Hemoglobin (MCH) | 20.95 pg | | 22.13 pg | |
| Mean Corpuscular Hemoglobin Concentration (MCHC) | 31.59 g/dL | | 32.67 g/dL | |
| **Leukocytes** | 6.80 x 10^3^/mm^3^ | | 3.29 x 10^3^/mm^3^ | |
|  | Relative (%) | Absolute (10^3^/mm^3^) | Relative (%) | Absolute (10^3^/mm^3^) |
| Basophils |  |  |  |  |
| Eosinophils | 3 | 0.20 | 1 | 0.03 |
| Neutrophils (Bands) |  |  |  |  |
| Neutrophils (Segmented) | 65 | 4.42 | 71 | 2.34 |
| Lymphocytes | 30 | 2.04 | 25 | 0.82 |
| Monocytes | 2 | 0.14 | 3 | 0.10 |
| **Platelets** | 455 x 10^3^/mm^3^ | | 351 x 10^3^/mm^3^ | |
| **ALT** | 22.87 U/L  (18 a 129 U/L) | | 69.07 U/L  (18 a 129 U/L) | |
| **AST** | 32.18 U/L  (23 a 70 U/L) | | 41.16 U/L  (23 a 70 U/L) | |
| **Creatinine** | 0.52 mg/dL  (0.30 a 1.30 mg/dL) | | 0.69 mg/dL  (0.30 a 1.30 mg/dL) | |
| **GGT** | 43.29 U/L  (48 a 130 U/L) | | 32.14 U/L  (48 a 130 U/L) | |

| **AG45** | **-1d pre-challenge*** | | **7d post-challenge** | |
| --- | --- | --- | --- | --- |
| **Erythrocytes** | 5.72 millions/mm^3^ | | 5.04 millions/mm^3^ | |
| Hemoglobin | 12.40 g/dL | | 11.50 g/dL | |
| Hematocrit (HCT) | 37.20 % | | 35.40 % | |
| Mean Corpuscular Volume (MCV) | 65.03 fm^3^ | | 70.23 fm^3^ | |
| Mean Corpuscular Hemoglobin (MCH) | 21.67 pg | | 22.81 pg | |
| Mean Corpuscular Hemoglobin Concentration (MCHC) | 33.33 g/dL | | 32.48 g/dL | |
| **Leukocytes** | 7.70 x 10^3^/mm^3^ | | 4.31 x 10^3^/mm^3^ | |
|  | Relative (%) | Absolute (10^3^/mm^3^) | Relative (%) | Absolute (10^3^/mm^3^) |
| Basophils |  |  |  |  |
| Eosinophils | 0 | 0 | 0 | 0 |
| Neutrophils (Bands) |  |  |  |  |
| Neutrophils (Segmented) | 69 | 5.31 | 73 | 3.15 |
| Lymphocytes | 21 | 1.62 | 22 | 0.95 |
| Monocytes | 10 | 0.77 | 5 | 0.22 |
| **Platelets** | 387 x 10^3^/mm^3^ | | 363 x 10^3^/mm^3^ | |
| **ALT** | 15.97 U/L  (18 a 129 U/L) | | 61.33 U/L  (18 a 129 U/L) | |
| **AST** | 23.69 U/L  (23 a 70 U/L) | | 53.82 U/L  (23 a 70 U/L) | |
| **Creatinine** | 0.51 mg/dL  (0.30 a 1.30 mg/dL) | | 0.65 mg/dL  (0.30 a 1.30 mg/dL) | |
| **GGT** | 104.80 U/L  (48 a 130 U/L) | | 50.60 U/L  (48 a 130 U/L) | |

| **AH37** | **-1d pre-challenge*** | | **7d post-challenge** | |
| --- | --- | --- | --- | --- |
| **Erythrocytes** | 5.54 millions/mm^3^ | | 4.80 millions/mm^3^ | |
| Hemoglobin | 11.90 g/dL | | 11.00 g/dL | |
| Hematocrit (HCT) | 38.00 % | | 33.10 % | |
| Mean Corpuscular Volume (MCV) | 68.59 fm^3^ | | 68.95 fm^3^ | |
| Mean Corpuscular Hemoglobin (MCH) | 21.48 pg | | 22.91 pg | |
| Mean Corpuscular Hemoglobin Concentration (MCHC) | 31.31 g/dL | | 33.23 g/dL | |
| **Leukocytes** | 9.20 x 10^3^/mm^3^ | | 6.02 x 10^3^/mm^3^ | |
|  | Relative (%) | Absolute (10^3^/mm^3^) | Relative (%) | Absolute (10^3^/mm^3^) |
| Basophils |  |  |  |  |
| Eosinophils | 0 | 0 | 2 | 0.12 |
| Neutrophils (Bands) |  |  |  |  |
| Neutrophils (Segmented) | 68 | 6.26 | 70 | 4.21 |
| Lymphocytes | 31 | 2.85 | 22 | 1.32 |
| Monocytes | 1 | 0.09 | 6 | 0.36 |
| **Platelets** | 369 x 10^3^/mm^3^ | | 364 x 10^3^/mm^3^ | |
| **ALT** | 20.15 U/L  (18 a 129 U/L) | | 57.05 U/L  (18 a 129 U/L) | |
| **AST** | 18.04 U/L  (23 a 70 U/L) | | 32.83 U/L  (23 a 70 U/L) | |
| **Creatinine** | 0.70 mg/dL  (0.30 a 1.30 mg/dL) | | 0.84 mg/dL  (0.30 a 1.30 mg/dL) | |
| **GGT** | 55.42 U/L  (48 a 130 U/L) | | 39.72 U/L  (48 a 130 U/L) | |

| **AC41** | **-1d pre-challenge^#^** | | **7d post-challenge** | |
| --- | --- | --- | --- | --- |
| **Erythrocytes** | 6.39 millions/mm^3^ | | 5.62 millions/mm^3^ | |
| Hemoglobin | 12.60 g/dL | | 11.60 g/dL | |
| Hematocrit (HCT) | 40.10 % | | 35.40 % | |
| Mean Corpuscular Volume (MCV) | 62.75 fm^3^ | | 62.98 fm^3^ | |
| Mean Corpuscular Hemoglobin (MCH) | 19.71 pg | | 20.64 pg | |
| Mean Corpuscular Hemoglobin Concentration (MCHC) | 31.42 g/dL | | 32.76 g/dL | |
| **Leukocytes** | 8.30 x 10^3^/mm^3^ | | 6.0 x 10^3^/mm^3^ | |
|  | Relative (%) | Absolute (10^3^/mm^3^) | Relative (%) | Absolute (10^3^/mm^3^) |
| Basophils |  |  |  |  |
| Eosinophils | 0 | 0 | 0 | 0 |
| Neutrophils (Bands) |  |  |  |  |
| Neutrophils (Segmented) | 77 | 6.39 | 75 | 4.50 |
| Lymphocytes | 20 | 1.66 | 19 | 1.14 |
| Monocytes | 3 | 0.25 | 5 | 0.30 |
| **Platelets** | 400 x 10^3^/mm^3^ | | 351 x 10^3^/mm^3^ | |
| **ALT** | 26.18 U/L  (18 a 129 U/L) | | 54.61 U/L  (18 a 129 U/L) | |
| **AST** | 33.28 U/L  (23 a 70 U/L) | | 41.95 U/L  (23 a 70 U/L) | |
| **Creatinine** | 0.70 mg/dL  (0.30 a 1.30 mg/dL) | | 1.05 mg/dL  (0.30 a 1.30 mg/dL) | |
| **GGT** | 84.64 U/L  (48 a 130 U/L) | | 51.96 U/L  (48 a 130 U/L) | |

| **AG46** | **-1d pre-challenge** | | **7d post-challenge** | |
| --- | --- | --- | --- | --- |
| **Erythrocytes** | 5.47 millions/mm^3^ | | 4.88 millions/mm^3^ | |
| Hemoglobin | 12.80 g/dL | | 11.00 g/dL | |
| Hematocrit (HCT) | 36.80 % | | 34.20 % | |
| Mean Corpuscular Volume (MCV) | 67.27 fm^3^ | | 70.08 fm^3^ | |
| Mean Corpuscular Hemoglobin (MCH) | 23.40 pg | | 22.54 pg | |
| Mean Corpuscular Hemoglobin Concentration (MCHC) | 34.78 g/dL | | 32.16 g/dL | |
| **Leukocytes** | 5.53 x 10^3^/mm^3^ | | 3.53 x 10^3^/mm^3^ | |
|  | Relative (%) | Absolute (10^3^/mm^3^) | Relative (%) | Absolute (10^3^/mm^3^) |
| Basophils |  |  | 1 | 0.04 |
| Eosinophils | 5 | 0.28 | 4 | 0.14 |
| Neutrophils (Bands) |  |  |  |  |
| Neutrophils (Segmented) | 65 | 3.60 | 75 | 2.65 |
| Lymphocytes | 24 | 1.33 | 19 | 0.67 |
| Monocytes | 6 | 0.33 | 1 | 0.04 |
| **Platelets** | 297 x 10^3^/mm^3^ | | 399 x 10^3^/mm^3^ | |
| **ALT** | 23.35 U/L  (18 a 129 U/L) | | 57.11 U/L  (18 a 129 U/L) | |
| **AST** | 37.62 U/L  (23 a 70 U/L) | | 47.01 U/L  (23 a 70 U/L) | |
| **Creatinine** | 0.60 mg/dL  (0.30 a 1.30 mg/dL) | | 0.83 mg/dL  (0.30 a 1.30 mg/dL) | |
| **GGT** | 39.60 U/L  (48 a 130 U/L) | | 24.43 U/L  (48 a 130 U/L) | |

| **AG128** | **-1d pre-challenge*** | | **7d post-challenge** | |
| --- | --- | --- | --- | --- |
| **Erythrocytes** | 5.45 millions/mm^3^ | | 4.62 millions/mm^3^ | |
| Hemoglobin | 11.30 g/dL | | 10.40 g/dL | |
| Hematocrit (HCT) | 33.40 % | | 31.20 % | |
| Mean Corpuscular Volume (MCV) | 61.28 fm^3^ | | 67.53 fm^3^ | |
| Mean Corpuscular Hemoglobin (MCH) | 20.73 pg | | 22.51 pg | |
| Mean Corpuscular Hemoglobin Concentration (MCHC) | 33.83 g/dL | | 33.33 g/dL | |
| **Leukocytes** | 7.70 x 10^3^/mm^3^ | | 4.78 x 10^3^/mm^3^ | |
|  | Relative (%) | Absolute (10^3^/mm^3^) | Relative (%) | Absolute (10^3^/mm^3^) |
| Basophils |  |  |  |  |
| Eosinophils | 0 | 0 | 1 | 0.05 |
| Neutrophils (Bands) | 0 | 0 |  |  |
| Neutrophils (Segmented) | 77 | 5.93 | 72 | 3.44 |
| Lymphocytes | 19 | 1.46 | 23 | 1.10 |
| Monocytes | 4 | 0.31 | 4 | 0.19 |
| **Platelets** | 464 x 10^3^/mm^3^ | | 360 x 10^3^/mm^3^ | |
| **ALT** | 31.90 U/L  (18 a 129 U/L) | | 90.48 U/L  (18 a 129 U/L) | |
| **AST** | 21.10 U/L  (23 a 70 U/L) | | 54.35 U/L  (23 a 70 U/L) | |
| **Creatinine** | 0.57 mg/dL  (0.30 a 1.30 mg/dL) | | 0.65 mg/dL  (0.30 a 1.30 mg/dL) | |
| **GGT** | 77.87 U/L  (48 a 130 U/L) | | 38.30 U/L  (48 a 130 U/L) | |

| **AG115** | **-1d pre-challenge** | | **7d post-challenge** | |
| --- | --- | --- | --- | --- |
| **Erythrocytes** | 5.35 millions/mm^3^ | | 4.68 millions/mm^3^ | |
| Hemoglobin | 12.10 g/dL | | 11.50 g/dL | |
| Hematocrit (HCT) | 35.40 % | | 35.40 % | |
| Mean Corpuscular Volume (MCV) | 66.16 fm^3^ | | 75.64 fm^3^ | |
| Mean Corpuscular Hemoglobin (MCH) | 22.61 pg | | 24.57 pg | |
| Mean Corpuscular Hemoglobin Concentration (MCHC) | 34.18 g/dL | | 32.48 g/dL | |
| **Leukocytes** | 6.0 x 10^3^/mm^3^ | | 3.35 x 10^3^/mm^3^ | |
|  | Relative (%) | Absolute (10^3^/mm^3^) | Relative (%) | Absolute (10^3^/mm^3^) |
| Basophils |  |  |  |  |
| Eosinophils | 1 | 0.06 | 2 | 0.07 |
| Neutrophils (Bands) |  |  |  |  |
| Neutrophils (Segmented) | 67 | 4.02 | 76 | 2.55 |
| Lymphocytes | 29 | 1.74 | 19 | 0.64 |
| Monocytes | 3 | 0.18 | 3 | 0.10 |
| **Platelets** | 361 x 10^3^/mm^3^ | | 387 x 10^3^/mm^3^ | |
| **ALT** | 28.34 U/L  (18 a 129 U/L) | | 92.66 U/L  (18 a 129 U/L) | |
| **AST** | 25.07 U/L  (23 a 70 U/L) | | 89.62 U/L  (23 a 70 U/L) | |
| **Creatinine** | 0.57 mg/dL  (0.30 a 1.30 mg/dL) | | 0.81 mg/dL  (0.30 a 1.30 mg/dL) | |
| **GGT** | 75.14 U/L  (48 a 130 U/L) | | 33.07 U/L  (48 a 130 U/L) | |

| **AG33** | **-1d pre-challenge** | | **7d post-challenge** | |
| --- | --- | --- | --- | --- |
| **Erythrocytes** | 5.25 millions/mm^3^ | | 4.49 millions/mm^3^ | |
| Hemoglobin | 11.70 g/dL | | 10.70 g/dL | |
| Hematocrit (HCT) | 35.60 % | | 32.60 % | |
| Mean Corpuscular Volume (MCV) | 67.80 fm^3^ | | 72.60 fm^3^ | |
| Mean Corpuscular Hemoglobin (MCH) | 22.28 pg | | 23.83 pg | |
| Mean Corpuscular Hemoglobin Concentration (MCHC) | 32.86 g/dL | | 32.82 g/dL | |
| **Leukocytes** | 8.40 x 10^3^/mm^3^ | | 4.48 x 10^3^/mm^3^ | |
|  | Relative (%) | Absolute (10^3^/mm^3^) | Relative (%) | Absolute (10^3^/mm^3^) |
| Basophils | 1 | 0.08 |  |  |
| Eosinophils | 1 | 0.08 | 0 | 0 |
| Neutrophils (Bands) |  |  |  |  |
| Neutrophils (Segmented) | 80 | 6.72 | 85 | 3.81 |
| Lymphocytes | 14 | 1.18 | 11 | 0.49 |
| Monocytes | 4 | 0.34 | 4 | 0.18 |
| **Platelets** | 341 x 10^3^/mm^3^ | | 385 x 10^3^/mm^3^ | |
| **ALT** | 44.89 U/L  (18 a 129 U/L) | | 60.33 U/L  (18 a 129 U/L) | |
| **AST** | 30.49 U/L  (23 a 70 U/L) | | 52.95 U/L  (23 a 70 U/L) | |
| **Creatinine** | 0.64 mg/dL  (0.30 a 1.30 mg/dL) | | 0.66 mg/dL  (0.30 a 1.30 mg/dL) | |
| **GGT** | 51.82 U/L  (48 a 130 U/L) | | 35.40 U/L  (48 a 130 U/L) | |

| **AB79** | **-1d pre-challenge^#^** | | **7d post-challenge** | |
| --- | --- | --- | --- | --- |
| **Erythrocytes** | 5.15 millions/mm^3^ | | 4.42 millions/mm^3^ | |
| Hemoglobin | 12.10 g/dL | | 10.70 g/dL | |
| Hematocrit (HCT) | 34.90 % | | 32.60 % | |
| Mean Corpuscular Volume (MCV) | 67.76 fm^3^ | | 73.75 fm^3^ | |
| Mean Corpuscular Hemoglobin (MCH) | 23.49 pg | | 24.20 pg | |
| Mean Corpuscular Hemoglobin Concentration (MCHC) | 34.67 g/dL | | 32.82 g/dL | |
| **Leukocytes** | 8.70 x 10^3^/mm^3^ | | 5.33 x 10^3^/mm^3^ | |
|  | Relative (%) | Absolute (10^3^/mm^3^) | Relative (%) | Absolute (10^3^/mm^3^) |
| Basophils |  |  |  |  |
| Eosinophils | 2 | 0.17 | 0 | 0 |
| Neutrophils (Bands) |  |  |  |  |
| Neutrophils (Segmented) | 67 | 5.83 | 72 | 3.83 |
| Lymphocytes | 24 | 2.09 | 25 | 1.33 |
| Monocytes | 7 | 0.61 | 3 | 0.16 |
| **Platelets** | 378 x 10^3^/mm^3^ | | 385 x 10^3^/mm^3^ | |
| **ALT** | 22.96 U/L  (18 a 129 U/L) | | 106.70 U/L  (18 a 129 U/L) | |
| **AST** | 22.60 U/L  (23 a 70 U/L) | | 64.34 U/L  (23 a 70 U/L) | |
| **Creatinine** | 0.74 mg/dL  (0.30 a 1.30 mg/dL) | | 1.00 mg/dL  (0.30 a 1.30 mg/dL) | |
| **GGT** | 77.46 U/L  (48 a 130 U/L) | | 42.30 U/L  (48 a 130 U/L) | |
